# A systematic comparison of statistical methods to detect interactions in exposome-health associations

**DOI:** 10.1186/s12940-017-0277-6

**Published:** 2017-07-14

**Authors:** Jose Barrera-Gómez, Lydiane Agier, Lützen Portengen, Marc Chadeau-Hyam, Lise Giorgis-Allemand, Valérie Siroux, Oliver Robinson, Jelle Vlaanderen, Juan R. González, Mark Nieuwenhuijsen, Paolo Vineis, Martine Vrijheid, Roel Vermeulen, Rémy Slama, Xavier Basagaña

**Affiliations:** 1 ISGlobal, Centre for Research in Environmental Epidemiology (CREAL), Dr. Aiguader, 88, Barcelona, 08003 Spain; 2 Universitat Pompeu Fabra (UPF), Plaça de la Merçè, 10-12, Barcelona, 08002 Spain; 3 CIBER Epidemiología y Salud Pública (CIBERESP), Av. Monforte de Lemos, 3-5 Pabellón 11. Planta 0, Madrid, 28029 Spain; 4 Team of Environmental Epidemiology applied to Reproduction and Respiratory Health, Inserm and University Grenoble Alpes, U823 Joint Research Center, Grenoble, France; 5 Institute for Risk Assessment Sciences, Utrecht University, Utrecht, Netherlands; 6 Department of Epidemiology and Biostatistics, MRC-PHE Centre for Environment and Health, School of Public Health, Imperial College London, Norfolk Place, W2 1PG London, UK; 7 MRC-PHE Centre for Environment and Health, School of Public Health, Imperial College London, London, UK

**Keywords:** Exposome, Interactions, Variable selection

## Abstract

**Background:**

There is growing interest in examining the simultaneous effects of multiple exposures and, more generally, the effects of mixtures of exposures, as part of the exposome concept (being defined as the totality of human environmental exposures from conception onwards). Uncovering such combined effects is challenging owing to the large number of exposures, several of them being highly correlated. We performed a simulation study in an exposome context to compare the performance of several statistical methods that have been proposed to detect statistical interactions.

**Methods:**

Simulations were based on an exposome including 237 exposures with a realistic correlation structure. We considered several statistical regression-based methods, including two-step Environment-Wide Association Study (EWAS_2_), the Deletion/Substitution/Addition (DSA) algorithm, the Least Absolute Shrinkage and Selection Operator (LASSO), Group-Lasso INTERaction-NET (GLINTERNET), a three-step method based on regression trees and finally Boosted Regression Trees (BRT). We assessed the performance of each method in terms of model size, predictive ability, sensitivity and false discovery rate.

**Results:**

GLINTERNET and DSA had better overall performance than the other methods, with GLINTERNET having better properties in terms of selecting the true predictors (sensitivity) and of predictive ability, while DSA had a lower number of false positives. In terms of ability to capture interaction terms, GLINTERNET and DSA had again the best performances, with the same trade-off between sensitivity and false discovery proportion. When GLINTERNET and DSA failed to select an exposure truly associated with the outcome, they tended to select a highly correlated one. When interactions were not present in the data, using variable selection methods that allowed for interactions had only slight costs in performance compared to methods that only searched for main effects.

**Conclusions:**

GLINTERNET and DSA provided better performance in detecting two-way interactions, compared to other existing methods.

**Electronic supplementary material:**

The online version of this article (doi:10.1186/s12940-017-0277-6) contains supplementary material, which is available to authorized users.

## Background

Many environmental exposures have been linked to health effects [1]. The fact that human biomonitoring and epidemiological studies are now able to measure a large number of environmental exposures in the same participants has led to the development of the exposome paradigm. The exposome is defined as the totality of human environmental exposures from conception onwards [2, 3]. As in genome studies, most exposome studies rely on holistic data-driven approaches to discover associations between the exposome and a health outcome. Environmental exposures can have independent effects on health outcomes, but a promising feature of exposome research lies in the promise to examine potentially interacting exposures or, more generally, the effects of mixtures of exposures [4–7]. Two- or three-way interactions between environmental exposures have been described in the literature and statistical methods to uncover interactions among a large set of exposures have been suggested [8–12].

In a recent paper, Agier et al. [13] studied the performance of several variable selection algorithms in an exposome context. In particular, they considered the Environment-Wide Association Study (EWAS) and a two-step version of EWAS based on multiple linear regression (EWAS-MLR), Elastic net (ENET), sparse partial least squares regression (sPLS), Graphical Unit Evolutionary Stochastic Search (GUESS) and the Deletion/Substitution/Addition (DSA) algorithm. Their results showed the limitations of all methods to select the right exposures when those exposures are correlated, although they showed that GUESS and DSA provided a marginally better balance between sensitivity and specificity than the other methods. However, their simulations did not consider the presence of interactions and most of the methods tested could not accommodate a search for interaction terms.

In this paper, we want to extend their work by considering scenarios with statistical interactions and by providing a systematic comparison of methods that have been recommended to search for interactions. We are also interested in the performance of those methods in the absence of real interactions. This is of interest, as when analysing real data one never knows whether such interactions exist or not. We will restrict our analyses to linear models with main effects of exposures and two-way interactions, as they are the most commonly reported in the literature and because they are easily parameterized in regression equations, hence facilitating the comparison between methods. Specifically, we considered two-step Environment-Wide Association Study (EWAS_2_), the Deletion/Substitution/Addition (DSA) algorithm, the Least Absolute Shrinkage and Selection Operator (LASSO), Group-Lasso INTERaction-NET (GLINTERNET), a three-step method based on regression trees and finally Boosted Regression Trees (BRT). Besides, we do not consider confounding by other covariates. The main focus of our analysis will be on variable selection, as we want to focus on the ability of the methods to correctly detect true associations. Other metrics such as bias or coverage of effect estimates will not be addressed, although we note that they will depend critically on the performance of variable selection.

The existing literature provides only a limited number of comparisons between the methods examined in this study and other alternatives. For instance, GLINTERNET was shown to perform comparably to the R package hierNet, with some advantages in computing time [8]. GLINTERNET performed better than boosting in terms of false discovery probability [8]. Under simulations, the DSA algorithm seemed to be competitive with Logic Regression, which only handles binary variables [14]. Sun et al. [10] compared Bayesian Model Averaging, DSA, LASSO, Partial Least-Squares Regression and Supervised Principal Component Analysis under simulations with up to 20 variables. They also considered a two-step modelling strategy in which variables were screened for inclusion in the second step using Classification and Regression Trees (CART). There was no uniform dominance of one method across all examined simulation scenarios. However, to the best of our knowledge, the methods considered in our study have never been systematically compared under the characteristics of the exposome context, i.e. variable selection and interactions detection with a high number of potentially correlated exposures.

## Methods

### Simulating data

The exposome data were simulated based on an existing dataset with the observation of 237 exposures on 655 individuals from the INMA (INfancia y Medio Ambiente) mother-child cohort [15]. In particular, we computed the correlation matrix of the exposures, ***Σ***. In such matrix, 81% of absolute pairwise correlations were lower than 0.2 while 64% were lower than 0.1. The median absolute value was 0.06 and the absolute percentiles 2.5^th^, 25^th^, 75^th^ and 97.5^th^ were 0.003, 0.03, 0.15 and 0.61, respectively. 78% of exposures were correlated at absolute level higher than 0.6 with at least one other exposure (see Additional file 1: Section A). Then, this matrix was used to simulate the exposures *E* using a multivariate normal distribution with mean ***0***,

1 $$  E \sim \mathrm{N}(\boldsymbol{0}, \boldsymbol{\Sigma}).  $$

The number of participants was set to *N*=1200. Subsequently, the outcome variable *Y* was simulated as

2 $$  Y = F(E) + \epsilon, \quad \epsilon \sim N(0, \sigma),  $$

where *F*(*E*) is a function of the exposome *E*, hereafter called the true model, and *σ* is the residual standard deviation. We considered three scenarios, displayed in Table 1, all of them involving five exposures, hereafter called true predictors as they are the ones generating the outcome. Scenarios are characterized by the relationship between the true predictors and the outcome, *F*(*E*). Thus, scenario 1 corresponds to a case with no interactions, scenario 2 corresponds to a case with one 2-way interaction, and scenario 3 corresponds to a case with two 2-way interactions. For each of the three scenarios, we built subscenarios according to the coefficient of determination (*R* ^2^) of the model (set at either 0.1 or 0.3); the pairwise correlation between the true predictors (either mixed: any exposure can be selected as a true predictor regardless of correlation; or high: exposures are chosen so that all their pairwise correlations are above 0.6); the size of interaction effects (either strong: same size as the main effects; or moderate: half the size of the main effects) (Additional file 1: Section B); and the direction of the interaction effect (either + : same direction than the main effects; or −: opposite direction to the main effects). Subscenarios are summarized also in Table 1. In each scenario, the size of both the main and the interaction effects, and the value of *σ* were tuned so that they yielded the desired *R* ^2^ and a sensitivity of around 0.9 or as close as possible when a model including only the true terms was fitted and their significance was assessed (sensitivity was the proportion of statistically significant terms over repeated simulations, see details on sensitivity tuning in an enlarged version of Table 1 in Additional file 1: Section C).

**Table 1:** Scenarios used to generate the data ^a^

Subscenario	Adjusted *R* ^2^	Pairwise corr. ^b^	Interaction size (and sign)	Parameters ^c^	
Scenario 1. True model: *F*(*E*)=*β*_0_+*β*_1_ *X*_1_+*β*_2_ *X*_2_+*β*_3_ *X*_3_+*β*_4_ *X*_4_+*β*_5_ *X*_5_ (Model size = 5; No interactions)					
1a	0.10 (0.07, 0.16)	Mixed			*σ*=7.5
1b	0.30 (0.23, 0.39)	Mixed			*σ*=3.8
1c	0.11 (0.09, 0.12)	High			*σ*=13
1d	0.27 (0.25, 0.28)	High			*σ*=7.5
Scenario 2. True model: *F*(*E*)=*β*_0_+*β*_1_ *X*_1_+*β*_2_ *X*_2_+*β*_3_ *X*_3_+*β*_4_ *X*_4_+*β*_5_ *X*_5_+*γ*_12_ *X*_1_ *X*_2_ (Model size = 6; Only one 2-way interaction)					
2a	0.09 (0.07, 0.14)	Mixed	Strong (+)	*γ*_12_=1	*σ*=8.3
2b	0.09 (0.06, 0.15)	Mixed	Strong (−)	*γ*_12_=−1	*σ*=8.3
2c	0.10 (0.06, 0.15)	Mixed	Moderate (+)	*γ*_12_=0.5	*σ*=7.8
2d	0.10 (0.07, 0.14)	Mixed	Moderate (−)	*γ*_12_=−0.5	*σ*=7.8
2e	0.13 (0.11, 0.14)	High	Strong (+)	*γ*_12_=1	*σ*=12
2f	0.13 (0.11, 0.15)	High	Strong (−)	*γ*_12_=−1	*σ*=12
2g	0.30 (0.28, 0.32)	High	Moderate (+)	*γ*_12_=0.5	*σ*=7
2h	0.30 (0.28, 0.32)	High	Moderate (−)	*γ*_12_=−0.5	*σ*=7
Scenario 3. True model: *F*(*E*) = *β*_0_+*β*_1_ *X*_1_+*β*_2_ *X*_2_+*β*_3_ *X*_3_+*β*_4_ *X*_4_+*β*_5_ *X*_5_+*γ*_12_ *X*_1_ *X*_2_+*γ*_13_ *X*_1_ *X*_3_ (Model size = 7; *X*_1_ involved in two 2-way interactions)					
3a	0.11 (0.08, 0.15)	Mixed	Strong (+)	*γ*_12_=*γ*_13_=1	*σ*=8.3
3b	0.10 (0.08, 0.16)	Mixed	Strong (−)	*γ*_12_=*γ*_13_=−1	*σ*=8.3
3c	0.10 (0.06, 0.14)	Mixed	Moderate (+)	*γ*_12_=*γ*_13_=0.5	*σ*=7.8
3d	0.10 (0.07, 0.14)	Mixed	Moderate (−)	*γ*_12_=*γ*_13_=−0.5	*σ*=7.8
3e	0.29 (0.27, 0.32)	High	Strong (+)	*γ*_12_=*γ*_13_=1	*σ*=8
3f	0.29 (0.27, 0.31)	High	Strong (−)	*γ*_12_=*γ*_13_=−1	*σ*=8
3g	0.31 (0.29, 0.33)	High	Moderate (+)	*γ*_12_=*γ*_13_=0.5	*σ*=7
3h	0.31 (0.28, 0.33)	High	Moderate (−)	*γ*_12_=*γ*_13_=−0.5	*σ*=7

^a^In each of the three scenarios, the outcome *Y* was generated as *Y*=*F*(*E*)+*ε*, where *F*(*E*) is a function of the predictors *X*_1_,…,*X*_5_, and *ε*∼*N*(0,*σ*). In each scenario, subscenarios were considered according to the pairwise correlation of the predictors (“Mixed”, when selecting the predictors among the whole exposome, in which case the absolute pairwise correlation ranged from 0.0000 to 1.0000; or “High”, when selecting the predictors among the subset of the 13 variables in the exposome for which all absolute pairwise correlations were 0.62 or higher); the size of the interaction terms (“Strong”, corresponding to equal size than the main effects size; or “Moderate”, corresponding to size 1/2 of the “Strong”), and the sign of the interaction terms (+ or −). Values for the adjusted *R* ^2^ correspond to the mean and percentiles 2.5^th^ and 97.5^th^ as a result of fitting the model to 100 simulated datasets. ^b^The median of the mean pairwise correlation between the true predictors was 0.12 (percentiles 2.5^th^ and 97.5^th^: (0.05, 0.25)) for “Mixed”, and 0.78 (percentiles 2.5^th^ and 97.5^th^: (0.72, 0.87)) for “High”. The median of the mean pairwise correlation between the true predictors and the other exposures was 0.13 (percentiles 2.5^th^ and 97.5^th^: (0.09, 0.16)) for “Mixed”, and 0.18 (percentiles 2.5^th^ and 97.5^th^: (0.17, 0.19)) for “High”. ^c^In all scenarios, *β*_0_=*β*_1_=⋯=*β*_5_=1

For each of the subscenarios in Table 1, we simulated 100 training datasets (*N*=1200 individuals) in which the statistical methods described below were applied. In each of the simulated datasets, true predictors were randomly selected from the available set of exposures. Likewise, we generated the same number of validation datasets (*N*=10000 individuals each) for the assessment of the out-of-sample prediction.

### Statistical methods

We considered several statistical methods previously recommended in the literature to detect interactions. Specifically, we focused on methods that implemented a variable selection approach to detect statistical interactions in the form of a product of two variables in a linear model. An exception was the inclusion of Boosted Regression Trees (BRT), which does not have an explicit regression equation. Besides, we restricted the simulation to methods that were or could easily be implemented in the R [16] software. The R code to reproduce this study is shown in Additional file 2.

#### Two-steps EWAS (EWAS_2_)

The Environment-Wide Association Study (EWAS) is a method analogous to genome-wide association studies but considering environmental factors instead of loci [17]. Thus, a univariate regression model is fitted for each exposure, and *p*-values are corrected for multiple comparisons. As an extension to search for two-way interactions, we used a two-step EWAS (EWAS_2_) as suggested by Kooperberg [11]. First, we fitted a simple linear regression model to test each exposure marginally at significance level *α*=0.05 and applying the Benjamini and Yekutieli correction [18] for multiple comparisons. All the significant exposures entered the second step of the process, in which we fitted a linear regression model with a pair of exposures and the corresponding two-way interaction term, and repeated the process for all possible pairs. *p*-values were corrected again for multiple comparisons using the Benjamini and Yekutieli correction and *α*=0.05. In the end, the selected terms in EWAS_2_ were all the main effects retained in the first step and all the two-way interaction terms that were significant in the second step. Note that this procedure does not provide a single model but just a set of exposures and a set of two-way interaction terms marginally associated with the outcome. However, such sets were used to assess the performance of EWAS_2_ through some of the measures defined later.

#### DSA algorithm

The Deletion/Substitution/Addition (DSA) algorithm [9] is an iterative process that starts with an empty model and uses deletion (removing a variable from the model), substitution (replacing a variable in the model by another not in the model) or addition (adding a variable in the model) moves to find the final model (further details are shown in Additional file 1: Section D). The final model is selected by minimizing the residual mean squared error (RMSE) using 5-fold cross-validation. We fitted two versions of DSA, one that only searches for main effects (DSA_1_) and another that also searches for 2-way interactions (DSA_2_). The way the DSA software is implemented, the version that searches for 2-way interactions also searches for quadratic terms. In all cases, we set the maximum model size to 10, which was never reached in the simulations. We used the R package DSA. It is noteworthy that this package, and the required package modelUtils, although working properly, are not included in the CRAN repository [19].

#### Sun 3-step method (Sun3step)

A 3-step method similar to that suggested by Sun et al. [10] was implemented (Sun3step). In the first step, we performed a correlation analysis to assess the collinearity within each group of exposures. In our data, there were 15 groups of exposures containing from 1 to 51 exposures each (see Additional file 1: Section A). When several exposures in the same group were highly correlated (Pearson correlation coefficient above 0.60), only the one with the smallest *p*-value in the single-exposure regression model was retained. In the second step, the selected exposures entered a Classification And Regression Tree (CART), which was subsequently pruned, with the criteria of minimizing the cross-validated error. The R package rpart was used. The variables selected in the construction of the regression tree entered the third step, which consisted of applying the DSA algorithm (allowing for 2-way interactions and quadratic terms), hence providing the final model.

#### Least absolute shrinkage and selection operator (LASSO)

LASSO is a method of estimation in linear models which penalizes large model sizes. Specifically, the method minimizes the residual sum of squares penalized by the sum of the absolute value of the regression coefficients, which tends to produce some coefficients being exactly zero and hence providing a variable selection procedure [20]. The LASSO method is not specifically designed to find interactions, but it was used to compare its performance with GLINTERNET, an extension of the LASSO method designed to look for interactions described below. Thus, no interaction terms were allowed in the LASSO method. We used 3-fold cross-validation for the sake of comparability with GLINTERNET. The R package glmnet was used.

#### Group-Lasso INTERaction-NET (GLINTERNET)

GLINTERNET is a variable selection algorithm that fits linear pairwise-interaction models that satisfy strong hierarchy: if an interaction coefficient is estimated to be nonzero, then its two associated main effects also have nonzero estimated coefficients [8]. It is based on the overlapped group-lasso [21], which considers the linear predictor as a linear combination of groups of terms (including main effects and two-way interactions). The particular case in which each group consists of only one variable corresponds to LASSO. Groups of variables are allowed to overlap, in the sense that one variable can be present in more than one group (e.g. the same variable can be present in two or more groups corresponding to different two-way interaction terms). In such cases, the final coefficient for a given variable is the sum of the coefficients of the groups in which the variable is present. A cross-validation using 3-folds (for computational reasons) was performed using the R package glinternet.

#### Boosted Regression Trees (BRT)

Lampa et al. [12] recommended the Boosted Regression Trees (BRT) as a tool to identify complex interactions. BRT combines the strengths of two algorithms: regression trees (models that relate a response to its predictors by recursive binary splits) and boosting (an adaptive method for combining many simple models to give improved predictive performance). The final BRT model can be understood as an additive regression model in which individual terms are simple trees, fitted in a forward, stage-wise fashion [22]. Hence, unlike the previously described techniques, BRT does not provide a simple regression equation. In regression trees, data are partitioned into a set of disjoint regions, and each region is assigned a constant value of the outcome variable. The splits of the trees can capture nonlinear effects and complex interactions. We set the maximum number of trees to 5000 and the depth parameter, which can be thought of as the maximum order of interactions, to 4. We modified the BRT method by incorporating a variable selection procedure described in Díaz-Uriarte [23]. With this addition, besides measures of prediction, the technique can be compared to the other methods in terms of its performance in variable selection. Briefly, the variable selection algorithm works as follows. We proceed iteratively by fitting BRT and eliminating at each iteration a fraction *f* (we set *f*=50*%*) of variables with the smallest importance. The importance of a given variable is based on the number of times it is selected for splitting, weighted by the squared improvement to the model as a result of each split, and averaged over all trees. Then, the final set of variables is chosen as the smallest set of variables which minimizes the out-of-sample error rate. Computations were performed using the R packages gbm and dismo.

### Measures of performance

To assess the performance of each method in each scenario, we estimated, among the simulated datasets, the mean value of the measures defined below. Such measures, analogous to those used by Agier et al. [13], are based on the comparison of the fitted models when using the assessed method and when using the model that already generated the data. Note that some measures are defined according to the terms in the model, while others are based on the variables involved in the model. For example, a model including *X*_1_, *X*_2_, $X_{1}^{2}$ and *X*_1_ *X*_2_ contains two variables and four terms.

- Relative model size (RMS): ratio of the fitted model size to the true model size. The model size is the number of terms in the model, excluding the intercept.
- Relative number of variables (RNV): ratio of the number of variables involved in the fitted model to the number of true predictors (i.e., variables involved in the true model).
- Relative out-of-sample *R* ^2^ ($R^{2}_{\text {rel}}$): ratio between the out-of-sample *R* ^2^ of the fitted model (numerator) and the out-of-sample *R* ^2^ of the model that includes only the terms used to generate the data (denominator), using a simulated test dataset (*N*=10000).
- Sensitivity (Sens): Proportion of terms in the true model correctly detected by the fitted model.
- Alternative sensitivity (AltSens)[13]: The average highest correlation between a true predictor and any variable involved in the fitted model,
  $$\text{AltSens} = \frac{1}{n_{A}} \sum\limits_{i \in A}\max_{j \in B}\left\{\text{corr}(X_{i}, X_{j})\right\}, $$

where *A* is the subset of true predictors, *n*_*A*_ is the number of true predictors, and *B* is the subset of variables in the fitted model. If all the true predictors were detected, both Sens and AltSens would take the value 1. If none of the true predictors were detected, but instead a set of variables having each a correlation of 0.9 with one of the true predictors were selected by the fitted model, AltSens would take the value 0.9 while Sens would take the value 0.

- Sensitivity for variables (Sensvar): proportion of true predictors involved in any term of the fitted model.
- False discovery proportion (FDP): Proportion of terms in the fitted model that are not in the true model.
- Alternative false discovery proportion (AltFDP)[13]: One minus the average highest correlation between a variable selected by the fitted model and any true predictor,
  $$\text{AltFDP} = 1 - \frac{1}{n_{B}} \sum\limits_{i \in B}\max_{j \in A}\left\{\text{corr}(X_{i}, X_{j})\right\}, $$

where *B* is the subset of variables involved in the fitted model and *n*_*B*_ is the size of *B*. If no false predictors were selected, both AltFDP and FDP would take the value 0. If none of the selected variables by the fitted model were a true predictor but each of them had a correlation of 0.9 with a true predictor, Alt FDP would take the value 0.1 but FDP would take the value 0.

- False discovery proportion for variables (FDPvar): Proportion of variables selected by the fitted model that are not true predictors.

Regarding the detection of interaction terms, we considered the following measures:

- Sensitivity for interaction terms (Sens_2_): Proportion of true interaction terms correctly detected by the fitted model.
- Alternative sensitivity for interaction terms (AltSens_2_), analogous to AltSens: the average of the highest correlation between a true predictor involved in an interaction term and a variable involved in an interaction term in the fitted model,
  $$\text{AltSens}_{2} = \frac{1}{n_{A_{2}}} \sum\limits_{i \in A_{2}}\max_{j \in B_{2}}\left\{\text{corr}(X_{i}, X_{j})\right\}, $$
  where *A*_2_ is the subset of true predictors involved in interaction terms, $n_{A_{2}}$ is the size of *A*_2_, and *B*_2_ the subset of the variables involved in interaction terms in the fitted model.
- False discovery proportion of interaction terms (FDP_2_): Proportion of interactions terms in the fitted model that are not in the true model.
- Alternative false discovery proportion of interaction terms (AltFDP_2_), analogous to AltFDP: The average of the highest correlation between a variable involved in an interaction term in the fitted model and any variable involved in an interaction term in the true model.
  $$\text{AltFDP}_{2} = 1 - \frac{1}{n_{B_{2}}} \sum\limits_{i \in B_{2}}\max_{j \in A_{2}}\left\{\text{corr}(X_{i}, X_{j})\right\}, $$
  where $n_{B_{2}}$ is the size of *B*_2_.

Note that some of the measures of performance cannot be computed for some models. Table 2 shows the features of each model according to both the availability of the measures of performance and the model structure.

**Table 2:** Characteristics and performance measures available for each method

Feature	EWAS_2_	DSA_1_	DSA_2_	Sun3step	LASSO	GLINTERNET	BRT
	Model structure						
Provides regression coefficients		✓	✓	✓	✓	✓	
Able to include interaction terms	✓		✓	✓		✓	✓
Able to include confounder covariates	✓	✓	✓	✓	✓		
Able to capture non-linear associations			✓	✓			✓
	Measures of performance						
RMS	✓	✓	✓	✓	✓	✓	
RNV	✓	✓	✓	✓	✓	✓	✓
$R^{2}_{\text {rel}}$		✓	✓	✓	✓	✓	✓
Sens	✓	✓	✓	✓	✓	✓	
AltSens	✓	✓	✓	✓	✓	✓	✓
Sensvar	✓	✓	✓	✓	✓	✓	✓
Sens_2_	✓		✓	✓		✓	
AltSens_2_	✓		✓	✓		✓	
FDP	✓	✓	✓	✓	✓	✓	
AltFDP	✓	✓	✓	✓	✓	✓	✓
FDPvar	✓	✓	✓	✓	✓	✓	✓
FDP_2_	✓		✓	✓		✓	
AltFDP_2_	✓		✓	✓		✓	

## Results

### Model size

**Fig. 1:**
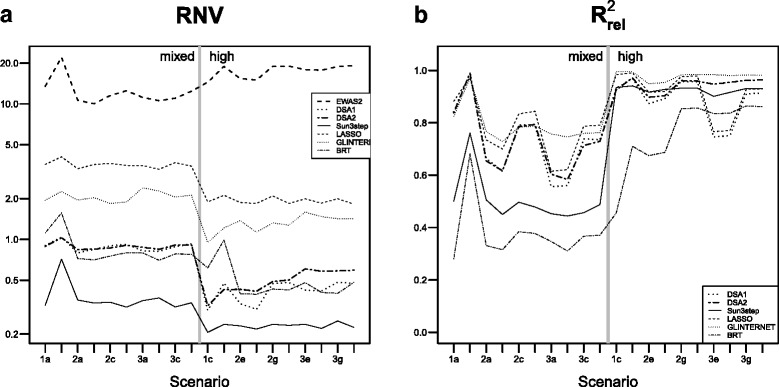
Performance of the compared methods in terms of number of variables in the fitted model and predictive ability. **a** Relative number of variables (RNV), in log scale, and **b** Relative out-of-sample *R* ^2^ ($R^{2}_{\text {rel}}$). Both measures are relative such that the true model corresponds to the value 1. Mean values based on 100 simulations. The vertical line separates scenarios according to the pairwise correlation between the true predictors as “Mixed” (any exposure can be selected as a true predictor regardless of correlation), or “High” (exposures are chosen so that all their pairwise correlations are above 0.6). Scenarios 1, 2 and 3 involve no interactions, one two-way interaction, and two two-way interactions, respectively

Figure 1(a) shows the number of variables in the model. Despite the application of a false discovery correction, EWAS_2_ selected between 10 and 20 times the number of true predictors. LASSO and GLINTERNET also selected more variables than in the true model, but to a much lower extent. Specifically, LASSO selected models with between 2 and 4 times the number of variables in the true model, while for GLINTERNET, this ratio ranged from 1 to 2, and was close to one in scenarios with high correlation between the true predictors. In contrast, DSA_1_, DSA_2_ and Sun3step selected fewer variables than the true model. DSA_2_ was the method closest to the right number of variables, with ratios of around 0.5 and 1, in scenarios with “Mixed” and “High” correlation, respectively. Sun3step was the most restrictive model, with a number of variables of around one third of that in the true model. Similar results were found when assessing the number of terms (as opposed to variables) included in the model (Additional file 1: Section E).

### Predictive ability

In terms of predictive ability, all methods achieved $R^{2}_{\text {rel}}$ between 0.3 and 1, i.e. *R* ^2^ lower than the model that includes only the terms used to generate the data (Fig. 1(b)). GLINTERNET was the method with the highest $R^{2}_{\text {rel}}$, being higher than 0.7 in all scenarios and very close to 1 in scenarios with high correlation between true predictors. Despite not considering interaction terms, LASSO achieved good values of $R^{2}_{\text {rel}}$, close to those of GLINTERNET, and similar or better than those of other methods except in scenarios with two strong interaction terms (scenarios 3e and 3f). DSA_2_ provided good values of $R^{2}_{\text {rel}}$, but lower than those of GLINTERNET, especially in cases with strong interactions and low correlations between predictors (scenarios 2a, 2b, 3a, and 3b). Sun3step and especially BRT provided the lowest values of $R^{2}_{\text {rel}}$, which in some scenarios were lower than 0.5.

### Sensitivity

**Fig. 2:**
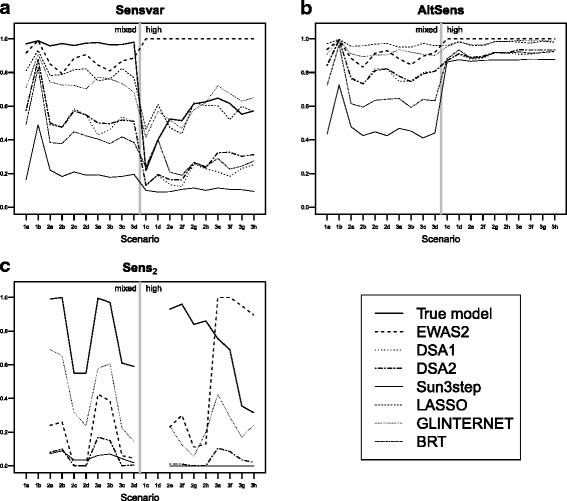
Performance of the compared methods in terms of sensitivity. **a** Sensitivity for variables (Sensvar), **b** Alternative sensitivity (AltSens), and **c** Sensitivity for interactions terms (Sens_2_). Mean values based on 100 simulations. The vertical line separates scenarios according to the pairwise correlation between the true predictors as “Mixed” (any exposure can be selected as a true predictor regardless of correlation), or “High” (exposures are chosen so that all their pairwise correlations are above 0.6). Scenarios 1, 2 and 3 involve no interactions, one two-way interaction, and two two-way interactions, respectively

EWAS_2_ was the method with the highest sensitivity for variables (Sensvar, Fig. 2(a)). In many cases, EWAS_2_ showed higher sensitivity than when fitting the model that included only the terms used to generate the data. This was the case because some of those terms were selected as significant in EWAS_2_ (i.e. in models including a single exposure at a time), but they were not significant when all terms were included in the same model. LASSO and GLINTERNET had values of sensitivity similar to each other, which were in turn very close to those of the model that included only the terms used to generate the data in all scenarios, especially in those with true interactions and high pairwise correlation among the predictors. DSA_1_ and DSA_2_ had similar sensitivity for variables, and they were about half of those of LASSO and GLINTERNET. BRT performed similarly to the DSA but with slightly smaller values. Sun3step had the worst sensitivity for variables among all methods. Results when assessing sensitivity for terms (as opposed to variables) showed the same patterns (Additional file 1: Section F). EWAS_2_, LASSO and GLINTERNET had values of AltSens (Fig. 2(b)) between 0.9 and 1, indicating that when they did not select a true predictor they selected a highly correlated exposure. Those values ranged from 0.7 to 0.9 for the DSA algorithms, and were lower for BRT and Sun3step.

In terms of the sensitivity for interaction terms (Sens_2_, Fig. 2(c)), GLINTERNET achieved substantially higher values than DSA_2_, Sun3step and EWAS_2_, except in scenarios with two interaction terms and high pairwise correlation (i.e. 3e, 3f, 3g and 3h), where EWAS_2_ was the best method. Results on AltSens_2_ are shown in the Additional file 1 (sections H and I). The values of this alternative measure of sensitivity were much higher than Sens_2_ for GLINTERNET, DSA_2_ and Sun3step, indicating that when a true interaction term was not selected an interaction term involving a highly correlated exposure was selected. For EWAS_2_, AltSens_2_ was much lower than for the other methods (see Additional file 1: Section I).

### False discovery proportion

**Fig. 3:**
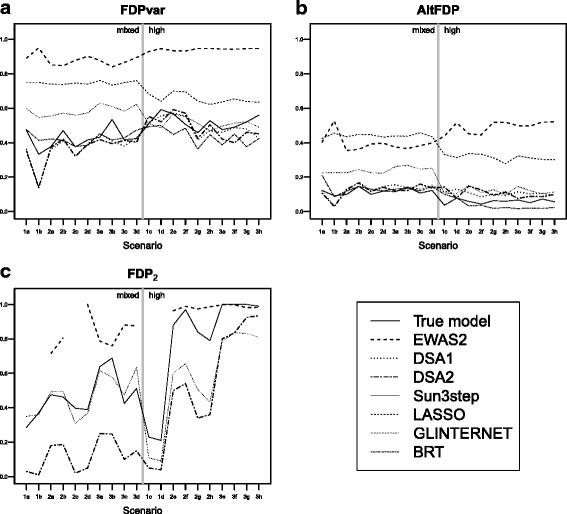
Performance of the compared methods in terms of specificity. **a** False discovery proportion for variables (FDPvar), **b** Alternative false discovery proportion (AltFDP), and **c** False discovery proportion for interaction terms (FDP_2_). Mean values based on 100 simulations. The vertical line separates scenarios according to the pairwise correlation between the true predictors as “Mixed” (any exposure can be selected as a true predictor regardless of correlation), or “High” (exposures are chosen so that all their pairwise correlations are above 0.6). Scenarios 1, 2 and 3 involve no interactions, one two-way interaction, and two two-way interactions, respectively

Regarding the proportion of wrongly selected exposures (FDPvar, Fig. 3(a)), all methods had values greater than 0.4. EWAS_2_ had values of around 0.9 for all scenarios. LASSO also had high values, greater than 0.7. GLINTERNET had values between 0.5 and 0.6. DSA_1_, DSA_2_, BRT and Sun3step tended to produce the lowest values. Similar results were obtained for the proportion of wrongly selected terms (as opposed to variables) associated with the outcome (FDP, Additional file 1: Section G). When looking at the alternative measure of false discovery (AltFDP, Fig. 3(a)), DSA_1_, DSA_2_, BRT and Sun3step tended to produce values lower than 0.15, indicating that when wrongly selecting an exposure, they tended to select one that was highly correlated to a true predictor. GLINTERNET produced slightly higher values, while EWAS_2_ and LASSO had values between 0.4 and 0.5.

In terms of false discovery for interaction terms (FDP_2_, Fig. 3(d)), EWAS_2_ performed worst, with values close to 1 in scenarios with high pairwise correlation among the true predictors and of around 0.9 in the other scenarios. DSA_2_ provided the lowest values, except in cases with two interaction terms and high correlation between true predictors (scenarios 3e to 3h), in which cases GLINTERNET provided better results. GLINTERNET tended to perform better than Sun3step. The alternative measure for false discovery proportion for interactions (AltFDP_2_, Additional file 1: Sections H and J) showed much lower values than FDP_2_. In particular, DSA_2_ had values below 0.1, indicating that when DSA_2_ wrongly selected an interaction term, the exposures involved in the selected interaction were highly correlated to the true ones. The other methods provided higher values.

The usual trade-off between sensitivity and false discoveries for both main effects and interaction terms was systematically observed under the different methods, i.e. no method maximized both (see Additional file 1: Section K).

### Impact of correlation between exposures

The pairwise correlation among the true predictors showed an important impact on method performance. In general, model size was reduced in the high correlation scenarios for all studied methods except EWAS_2_. $R^{2}_{\text {rel}}$ was mostly above 0.8 for high correlation while it ranged from 0.3 to 0.9 for mixed correlation. Regarding sensitivity, higher correlation was associated with a reduction in both Sens and Sens_2_ (while there was no clear pattern for Sensvar) but with an increase in AltSens (always above 0.8 for high correlation scenarios) to the point to achieve higher $R^{2}_{\text {rel}}$ (mostly above 0.8 for high correlation while it ranged from 0.3 to 0.9 for mixed correlation). In terms of false discoveries, almost no changes were observed, except an increase in FDP_2_ in high correlation scenarios.

In addition, we performed a sensitivity analysis (Additional file 1: Section L) for the impact of a low pairwise correlation among the true predictors on the performance of the analysed methods. Specifically, we created the new scenario 2i, which was tuned to be similar to scenarios 2a and 2e, but differing in the pairwise correlation among the true predictor. In scenario 2i, the true predictors are selected among the subset of 13 exposures for which all pairwise correlations are 0.1 or lower, while in scenarios 2a and 2e such correlations were “Mixed” and “High”, respectively. Results showed almost no changes regarding model size and $R^{2}_{\text {rel}}$. Sensitivity decreased around 40% and FDP increased around 30% for almost all methods when changing from “low” to “High” pairwise correlation, although the alternative measures (i.e. AltSens and AltFDP) remained in general invariant.

### Scenarios with no interaction

Both DSA_2_ and GLINTERNET are able to look for interaction terms. DSA_1_ and LASSO can be seen, respectively, as particular cases of those methods, restricted to look for main effects only. Table 3 shows the relative performance of these two pairs of methods regarding sensitivity and FDP in scenarios with no real interaction (1a to 1d). For DSA, looking for interactions when they do not exist had almost no cost in terms of sensitivity (variation between –4 and 2%). The difference in FDP ranged from –6 to 7%. When comparing GLINTERNET with LASSO, looking for interactions reduced the sensitivity by 3 to 12%, but led to a reduction in FDP between 19 and 25%. That is, GLINTERNET detected fewer true predictors but it also detected fewer false predictors than LASSO.

**Table 3:** Cost of testing for interactions in cases where they do not exist ^a^

	Scenario 1a		Scenario 1b		Scenario 1c		Scenario 1d	
Ratio of measures	Sens	FDP	Sens	FDP	Sens	FDP	Sens	FDP
DSA_2_ to DSA_1_	1.00	0.98	1.00	0.96	0.96	1.07	1.02	0.94
GLINTERNET to LASSO	0.88	0.81	0.97	0.75	0.89	0.75	0.93	0.77

^a^Restricted to methods having a version for main effects only and a version for main effects and interactions. Figures in the table represent the ratio of performance measure between the version looking only for main effects (denominator) and the version looking also for interaction terms (numerator)

## Discussion

We conducted a simulation study in an exposome context comparing the performance of several statistical methods that have been recommended to detect interactions. In addition, two methods that are not able to detect interactions (LASSO and DSA_1_) were also considered for comparison purposes. Of the tested methods, GLINTERNET and DSA_2_ showed the best overall performance, with GLINTERNET having better properties in terms of sensitivity and predictive ability, and DSA_2_ giving lower values of false discovery measures. GLINTERNET and DSA_2_ also performed best when capturing interaction terms, with the same trade-off between sensitivity and false discovery proportion. When interactions were not present in the data, using variable selection methods that allow for interactions had almost no cost in sensitivity and only a slight reduction in false discovery rate, compared to methods that only search for main effects.

Both GLINTERNET and DSA_2_ have some specific features. GLINTERNET forces the main effects in the model when an interaction term is detected, as it is commonly done in practice, although this is not the case for DSA_2_. The DSA algorithm allows for including interactions of higher orders and, when the order of interactions is set to 2, the model also looks for quadratic effects. Interestingly, both GLINTERNET and DSA_2_ can be considered generalizations of variable selection methods that only search for main effects. In our simulations, when using the the DSA method, looking for interactions when they do not exist had a small effect on sensitivity and produced also a small reduction of FDP, of up to 7%. Given these numbers, researchers may decide if it is worth the cost including the search for interactions in their analyses. The comparison between LASSO and its generalization GLINTERNET was less clear. Looking for interactions implied a reduction in sensitivity, but FDP was actually improved up to 25%. This may be explained by the fact that the two algorithms are not exactly comparable, as the penalty in GLINTERNET affects groups of coefficients.

EWAS_2_, i.e. a two-step method that searches for interactions without including all variables in the same model, offered a poor performance, with a very high percentage of false positives despite the multiple comparison correction. This is in agreement with the poor performance of the EWAS method in a similar simulation study that did not consider interactions [13]. EWAS_2_ had the highest sensitivity because many exposures, including the true predictors, were selected. This result did not extend to the detection of interactions terms (e.g. GLINTERNET had better sensitivity than EWAS_2_). This may be due to the high number of interaction terms that are tested in the second stage as a result of the high number of exposures selected in the first stage, and the multiple comparison correction.

Sun et al. [10] recommended a three-step method, in which in the first step only one exposure per family is retained. Thus, by definition, this method will miss some true predictors if there are more than one true predictor in the same family. We repeated the analysis excluding this first step, and the performance of the Sun3step method only changed minimally (data not shown). This approach achieved the lowest sensitivity, an $R^{2}_{\text {rel}}$ substantially lower and a FDR higher than for other methods. Similar performances were observed when looking at the interaction terms only.

BRT is a method that differs from the rest in that it has no regression equation for the final model and that it does not formally perform variable selection. In this study, we embedded a variable selection procedure to BRT. It is possible that such variable selection may have reduced the performance of the method, although it was implemented to minimize the out-of-sample error. In fact, BRT had one of the lowest sensitivities, although FDP was low and comparable to DSA_2_. Despite BRT is mainly seen as a predictive method, it produced the lowest $R^{2}_{\text {rel}}$. This can be partly explained by the way the data was simulated. The true model has a linear equation form, hence regression methods may be more suited to capture those effects. Thus, it is possible that BRT had better performances in more complex scenarios.

The pairwise correlation among the true predictors revealed as one of the main drivers of method performance, in some cases being even more important than the presence of interaction terms. Specifically, when such correlations were high, selected models tended to be smaller and it was more difficult to select true terms, as correlated exposures were selected instead. In terms of prediction, such models where a correlated exposure was selected instead of a true one could result in higher $R^{2}_{\text {rel}}$. However, epidemiological studies are usually not focused on prediction but on identifying causal associations. The latter task becomes more difficult in settings with highly correlated exposures.

We have performed sensitivity analyses to assess the impact of the tuning of the main parameters for DSA_2_, LASSO, GLINTERNET, BRT and Sun3step. Results showed only some slight, almost always non significant changes in the performance of the methods, which did not change the conclusions of the study (Additional file 1: Section M).

Statistical interactions are scale dependent, so our results depend on the assumed underlying model. Researchers interested in the causal interpretation of interactions should refer to the methods described in VanderWeele [24], although most of them are developed for binary exposures and outcomes. In this paper we only considered two-way interactions. It is likely that more complex interactions between environmental exposures exist. Yet, higher order interactions are complex to interpret [25] and are usually not investigated. Although some papers, usually with predefined hypotheses, have reported 3rd and higher order interactions, the fact that studies often have low power to detect them precludes their examination [26, 27]. Nevertheless, future comparison of methods in a context of higher order interactions would be of interest. The problem of the effects of mixtures of pollutants is of high interest, and alternative methods have been suggested to address that problem. For example, Bobb et al. [28] suggest a method based on Bayesian kernel machine regression that incorporates variable selection and even a hierarchical variable selection procedure that accounts for structure in the exposures (e.g. families of highly correlated exposures). This method, implemented in the bkmr R package, can capture complex exposure-response functions of mixtures of exposures. Another example is the Bayesian Profile regression method [29], implemented in the PReMiuM R package, which aims at finding clusters of subjects sharing similar exposure profiles that at the same time show differences in the outcome. The inclusion of these two techniques in our simulation setting was not computationally feasible. However, the use of either of those techniques is not computationally problematic for the analysis of a single dataset of a similar size as the ones used here, so they remain as two attractive techniques to be considered in practice. These two methods can also capture complex non-linear associations. Our simulations did not consider non-linear effects, but some of the techniques used, such as DSA, Sun3step and BRT would be able to capture them (Table 2).

The present study considered a limited number of scenarios. In particular, we only considered linear regression models and we did not consider issues such as non-linear main effects or the effect of confounders that are not in the set of exposures of interest. Even in that restricted setting, the number of scenarios considered was small, as many other combinations of parameters could be used. This is an issue in all simulation studies, but the presence of interaction terms adds another layer to the number of potential scenarios to be tested. We based our simulation on realistic scenarios using existing data, and included scenarios with different degrees of correlation between true predictors, different number of interaction terms with different strengths and directions, and different levels of *R* ^2^ of the models. Although many more scenarios could have been investigated, we believe we covered a large range of realistic scenarios and included extreme situations acting as stress test simulations for the methods assessment.

In practice, epidemiological studies have a set of confounders that need to be included in the model to obtain unbiased estimates of the effects of exposures (e.g. socio-demographic variables or seasonal trends). We did not consider that situation on our simulations, but we expect that considering confounders would only change the initial conditions of the scenarios (e.g. some exposures would not have true effects after confounder adjustment, and the residual variation of the model may be reduced). However, in practice it is important that models allow for the possibility to force confounders into the model. All of the methods analysed except GLINTERNET and BRT allow for this possibility (Table 2). For the other methods, one would need to use other approaches to deal with confounding, such as fitting an initial regression model with just the confounders, and performing the variable selection of exposures in a second stage using the residuals of that model.

## Conclusions

This study confirms that exposome-health studies are likely, in the context of limited sample sizes of about 1000 individuals and of the agnostic regression-based statistical methods we considered, to suffer from a high rate of false positive signals. This weakness could be explained by the presence of correlation between exposures. However, considering interactions did not imply a very high additional cost in terms of sensitivity or false discovery proportion with the approaches we considered. Specifically, our results showed that GLINTERNET and DSA_2_ are two techniques that can be used to search for two-way statistical interactions in the exposome context, if one can assume linearity of effects. Although model selection is a hard task when a large number of potential predictors are available, these two techniques provided better performance than other methods that have been previously suggested for interaction detection.

## Additional files

Additional file 1 Supplementary results (tables and figures). (PDF 1220 kb)

Additional file 2 R code to reproduce this study (script). (R 147 kb)

## Abbreviations

AltSens: Alternative sensitivity
AltFDP: Alternative false discovery proportion
AltSens_2_: Alternative sensitivity for interaction terms
AltFDP_2_: Alternative false discovery proportion of interaction terms
BRT: Boosted Regression Trees
CART: Classification And Regression Trees
DSA: Deletion/Substitution/Addition
EWAS_2_: Two-steps environment-wide association study
FDP: False discovery proportion
FDPvar: False discovery proportion for variables
FDP_2_: False discovery proportion of interaction terms
GLINTERNET: Group-Lasso INTERaction-NET
INMA: INfancia y Medio Ambiente
LASSO: Least absolute shrinkage and selection operator
RMSE: Residual mean squared error
RMS: Relative model size
RNV: Relative number of variables
*R*^2^: Coefficient of determination
$R^{2}_{ ext {rel}}$: Relative out-of-sample *R* ^2^
Sun3step: Sun 3-step method
Sens: Sensitivity
Sensvar: Sensitivity for variables
Sens_2_: Sensitivity for interaction terms
