# Supplementary material for: A systematic comparison of statistical methods to detect interactions in exposome-health associations
Source: Environ Health. 2017 Jul 14;16:74. doi: 10.1186/s12940-017-0277-6 (PMC5513197; doi:10.1186/s12940-017-0277-6)
Supplement: Supplementary file 1 — Supplementary results (tables and figures). (PDF 1220 kb) [file 12940_2017_277_MOESM1_ESM.pdf]

Additional file 1. Supplementary results for

"A systematic comparison of statistical methods to detect interactions in exposome-health associations"

by J. Barrera-Gómez *et al.*

Contents

A Correlation matrix of the exposures and families of exposures 3

B Tuning the size of the interaction terms 4

C Scenarios used to generate the data 6

D Description of the DSA algorithm 6

E Performance in terms of relative model size (RMS) 6

F Performance in sensitivity for terms (Sens) 10

G Performance in false discevory proportion of terms (FDP) 10

H Numerical results on the performance of the models in all scenarios 10

I Performance in $\mathbf{AltSens_2}$ 12

J Performance in $\mathbf{AltFDP_2}$ 12

K Trade-off between sensitivity and specificity 12

L Sensitivity analyses for the impact of low pairwise correlation among the true predictors 55

M Sensitivity analyses on the main tuning parameters 59
- M.1 DSA_2 . . . 59
- M.2 LASSO . . . 59
- M.3 GLINTERNET . . . 59
- M.4 BRT . . . 60
- M.5 Sun3step . . . 61
  - M.5.1 Step 1: correlation analysis . . . 61
  - M.5.2 Step 2: CART screening . . . 62
  - M.5.3 Step 3: DSA_2 fitting . . . 63
- M.6 Summary of results . . . 65

A Correlation matrix of the exposures and families of exposures

The exposome were organized in 15 families of exposures. The number and names of exposures in each family were the following:

```
## $`Air pollutants`
##  [1] "NO2_T1"     "NO2_T2"     "NO2_T3"     "NO2_preg"   "Ben_T1"     "Ben_T2"     "Ben_T3"
##  [8] "Ben_preg"   "NO2_T_E"    "NO_T"       "PM25_T"     "PM10_T"     "PMcoarse_T" "AbsPM25_T"
## [15] "PM25CU_T"   "PM25FE_T"   "PM25K_T"    "PM25NI_T"   "PM25S_T"    "PM25SI_T"   "PM25V_T"
## [22] "PM25ZN_T"   "PM10CU_T"   "PM10FE_T"   "PM10K_T"    "PM10NI_T"   "PM10S_T"    "PM10SI_T"
## [29] "PM10V_T"    "PM10ZN_T"
##
## $`Built environment`
## [1] "BDENS100" "BDENS300" "CONN100"  "CONN300"  "T_WAVG"
##
## $Cotinine
## [1] "lcotinine"
##
## $`Green Space`
## [1] "NDVI100" "NDVI250" "NDVI500"
##
## $`Indoor air`
## [1] "gascooking"        "ETS"               "cleaning_products" "home_pesticides"
## [5] "garden_pesticides"
##
## $Metals
##  [1] "loghg"    "logpb"    "lcrCo_T1" "lcrNi_T1" "lcrCu_T1" "lcrZn_T1" "lcrAs_T1" "lcrSe_T1"
##  [9] "lcrMo_T1" "lcrCd_T1" "lcrSb_T1" "lcrCs_T1" "lcrTl_T1" "lcrPb_T1" "lcrCo_T3" "lcrNi_T3"
## [17] "lcrCu_T3" "lcrZn_T3" "lcrAs_T3" "lcrSe_T3" "lcrMo_T3" "lcrCd_T3" "lcrSb_T3" "lcrCs_T3"
## [25] "lcrTl_T3" "lcrPb_T3"
##
## $Noise
## [1] "TVLDIA" "TVLVES" "TVLNIT" "TVLDEN"
##
## $Nutrients
##  [1] "CALS"                "PROT"                "CARB"                "FAT_T"
##  [5] "FAT_S"               "FAT_M"               "FAT_P"               "OMEGA3"
##  [9] "OMEGA6"              "CHOL"                "FIBER"               "VITA"
## [13] "RETINOL"             "CAROT"               "B6"                  "B12"
## [17] "FOLA"                "VITC"                "VITD"                "VITE"
## [21] "CALC"                "IRON"                "MAGN"                "POTAS"
## [25] "SOD"                 "ZINC"                "CMLPX"               "SUGAR"
## [29] "ALCO"                "CAFF"                "H2O"                 "Ag16_0_Palmitico"
## [33] "Ag18_0_Estearico"    "Ag18_1_Oleico"       "Ag18_2_Linoleico"    "Ag18_3_Linolenico"
## [37] "Ag20_4_Araquid_nico" "Ag20_5_EPA"          "Ag22_6_DHA"          "FIBI"
## [41] "YODO"                "YODADA"              "FIBS"                "TRANS"
## [45] "A_CAR"               "B_CAR"               "CRYPTO"              "LUT_ZE"
## [49] "LYCO"                "SugarA"              "AHEI"
##
## $Organochlorines
## [1] "ldde_lip"    "lddt_lip"    "lhcb_lip"    "lbhch_lip"   "lpcb118_lip" "lpcb153_lip"
## [7] "lpcb180_lip" "lpcb138_lip"
##
## $PBDEs
##  [1] "lbde17_lip"  "lbde28_lip"  "lbde71_lip"  "lbde47_lip"  "lbde66_lip"  "lbde100_lip"
##  [7] "lbde99_lip"  "lbde85_lip"  "lbde154_lip" "lbde153_lip" "lbde138_lip" "lbde183_lip"
```

```
## [13] "lbde190_lip" "lbde209_lip"
##
## $PFAS
## [1] "logcbPFHxS" "logcbPFOA"  "logcbPFOS"  "logcbPFNA"  "logmPFHxS"  "logmPFOA"   "logmPFOS"
## [8] "logmPFNA"
##
## $Phenols
## [1] "lcrbpa_av"   "l_24_dcp_T3" "l_25_dcp_T3" "lb_pb_T3"    "le_pb_T3"    "lm_pb_T3"
## [7] "lp_pb_T3"    "lbp_3_T3"    "ltcs_T3"
##
## $Phthalates
##  [1] "lcrMEHP_av"      "lcrMEHHP_av"     "lcr_5cxMEPP_av"  "lcr_2cxMMHP_av"  "lcrMEP_av"
##  [6] "lcrMiBP_av"      "lcrMnBP_av"      "lcr_7OHMMeOP_av" "lcrMBzP_av"      "lcrMEOHP_av"
##
## $`w32 Nutrients`
##  [1] "w32_CALS"                 "w32_PROT"                "w32_CARB"
##  [4] "w32_FAT_T"                "w32_FAT_S"               "w32_FAT_M"
##  [7] "w32_FAT_P"                "w32_OMEGA3"              "w32_OMEGA6"
## [10] "w32_CHOL"                 "w32_FIBER"               "w32_VITA"
## [13] "w32_RETINOL"              "w32_CAROT"               "w32_B6"
## [16] "w32_B12"                  "w32_FOLA"                "w32_VITC"
## [19] "w32_VITD"                 "w32_VITE"                "w32_CALC"
## [22] "w32_IRON"                 "w32_MAGN"                "w32_POTAS"
## [25] "w32_SOD"                  "w32_ZINC"                "w32_CMLPX"
## [28] "w32_SUGAR"                "w32_ALCO"                "w32_CAFF"
## [31] "w32_H2O"                  "w32_Ag16_0_Palmitico"    "w32_Ag18_0_Estearico"
## [34] "w32_Ag18_1_Oleico"        "w32_Ag18_2_Linoleico"    "w32_Ag18_3_Linolenico"
## [37] "w32_Ag20_4_Araquid_nico" "w32_Ag20_5_EPA"          "w32_Ag22_6_DHA"
## [40] "w32_FIBI"                 "w32_YODO"                "w32_YODADA"
## [43] "w32_FIBS"                 "w32_TRANS"               "w32_A_CAR"
## [46] "w32_B_CAR"                "w32_CRYPTO"              "w32_LUT_ZE"
## [49] "w32_LYCO"                 "w32_SugarA"              "w32_AHEI"
##
## $`Water DBPs`
##  [1] "lTHM_preg"   "lTHM_T1"     "lTHM_T2"     "lTHM_T3"     "lCHCL3_preg" "lCHCL3_T1"
##  [7] "lCHCL3_T2"   "lCHCL3_T3"   "lBROM_preg"  "lBROM_T1"    "lBROM_T2"    "lBROM_T3"
```

The correlation matrix of the exposures is shown in Figure 1.

B Tuning the size of the interaction terms

We checked examples of size of interactions in the literature, which were difficult to find, as most papers reporting an interaction do not report the interaction coefficient but instead perform some kind of categorization of the exposures to illustrate the results. In one of the papers reporting interactions coefficients, they were half the size of the main effects but it is difficult to interpret, since variables in the model were log transformed.[1] Nevertheless, it is hard to define a "strong" or "moderate" interation, since the ratio between α_{ij} and β_i dramatically depends on the units of the variables X_i and X_j. For instance, suppose we fit the two following models to the same dataset:

$$\begin{aligned} \text{Model 1} &: \; Y = \beta_0 + \beta_1 X_1 + \beta_2 X_2 + \alpha_{12} X_1 X_2 \\ \text{Model 2} &: \; Y = \tilde{\beta}_0 + \tilde{\beta}_1 Z_1 + \tilde{\beta}_2 Z_2 + \tilde{\alpha}_{12} Z_1 Z_2, \quad Z_i := \tfrac{X_i - m_i}{s_i}, \end{aligned}$$

where m_i and s_i are the sample mean and standard deviation of X_i, respectively. Both models describe exactly the same relationship between Y and the predictors X_1 and X_2 but in different units.

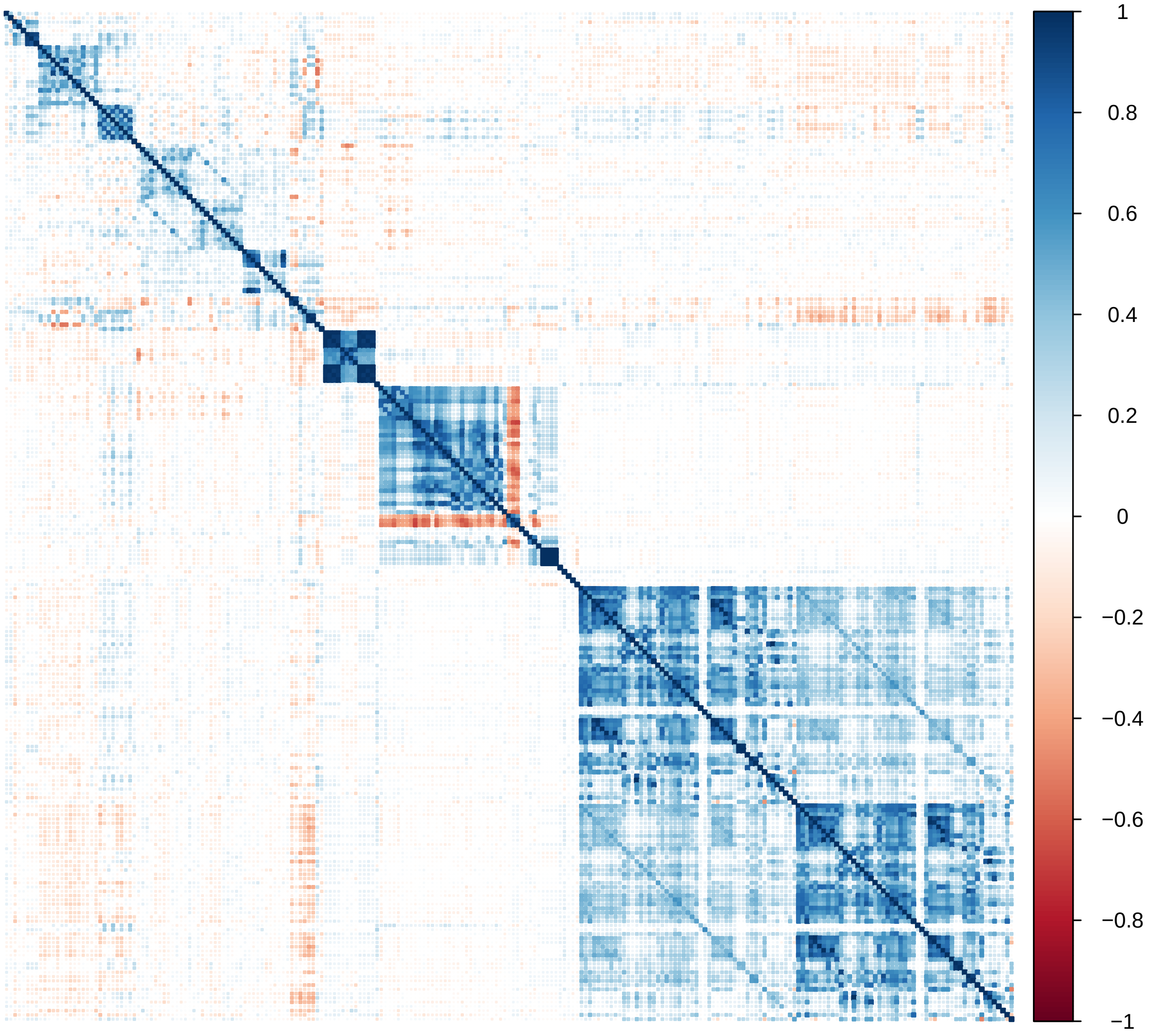

Figure 1: Correlation matrix of the exposures used for data generation.

However, the relative sizes of effects α_{12}/β_i and $\tilde{\alpha}_{12}/\tilde{\beta}_i$ can be numerically incomparable depending on the sample mean and deviation of the X_i, and also on the sizes of the effects. Specifically,

$$\frac{\tilde{\alpha}_{12}/\tilde{\beta}_i}{\alpha_{12}/\beta_i} = \frac{\beta_i\, s_{3-i}}{\beta_i + \alpha_{12}\, m_{3-i}}.$$

Then, we decided to tune the value of the α_{ij} by computing the change in the effect of X_1 on the outcome Y when varying the value of X_2. In Model 1, the effect on the outcome Y due to increasing c units in X_1 for a given value of $X_2 = x_2$ is

$$\Delta\mathbb{E}(Y|\Delta X_1 = c, X_2 = x_2) = (\beta_1 + \alpha_{12}\, x_2),$$

so the relative change of such effect when varying X_2 from the percentiles p to $100-p$, $X_2 = q_p$ and $X_2 = q_{100-p}$, respectively, is

$$r := \frac{1 + (\alpha_{12}/\beta_1)q_{100-p}}{1 + (\alpha_{12}/\beta_1)q_p}.$$

It can be easily shown that r does not depend on the units of the variables (i.e. it is the same under Model 1 and under Model 2). If $X_2 \sim \mathrm{N}(0,1)$ as in our case, r simplifies to

$$r = \frac{1 + (\alpha_{12}/\beta_1)q_{100-p}}{1 - (\alpha_{12}/\beta_1)q_{100-p}},$$

which is graphically represented in Figure 2. According to results in Figure 2, we decided a tuning based on the red line was reasonable. In that case, changing X_2 from the first to the third quartile implies a 2-fold ("moderate") in the effect of X_1 on Y for $\alpha_{12}/\beta 1 = 0.5$, and a 5-fold ("strong") for $\alpha_{12}/\beta 1 = 1$.

C Scenarios used to generate the data

See Table 1.

D Description of the DSA algorithm

The Deletion/Substitution/Addition (DSA) algorithm[2] is an iterative process that starts with an empty model and uses deletion (removing a variable from the model), substitution (replacing a variable in the model by another not in the model) or addition (adding a variable in the model) moves to find the final model. The acceptation of moves is based on the loss function, which is L2.

Given a model of size p, the first step consists of considering the p models of size $p-1$ resulting of deleting one of each of the p variables. If the minimum of the p associated values of the loss function is lower than that for the p-size model, the corresponding deletion is accepted and the deletion process iterates. Otherwise, the substituion moves start, subtituting variables one by one in the same way than the deletion moves. Finally, the addition moves are considered similarly, adding variables one by one. The final model among the optimal models for each possible model size is then selected by cross-validation.

E Performance in terms of relative model size (RMS)

See Figure 3.

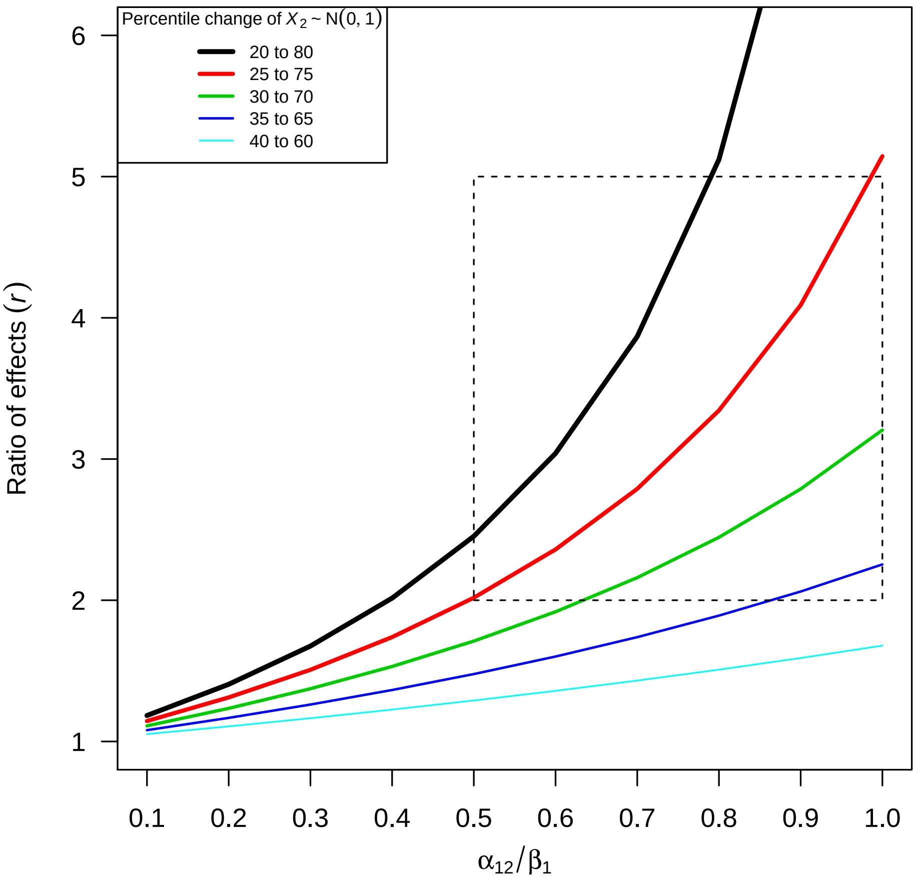

Figure 2: Ratio of effects of X_1 on Y when varying $X_2 \sim \mathrm{N}(0,1)$ from percentile p to percentile $100-p$, $r = \frac{\Delta\mathbb{E}(Y|\Delta X_1=c, X_2=q_{1-p})}{\Delta\mathbb{E}(Y|\Delta X_1=c, X_2=q_p)} = \frac{1+(\alpha_{12}/\beta_1)q_{100-p}}{1-(\alpha_{12}/\beta_1)q_{100-p}}$.

Table 1: Scenarios used to generate the data. In each of the three scenarios, the outcome Y was generated as $Y = F(E) + \epsilon$, where $F(E)$ is a function of the predictors $X_1, \ldots, X_5$, and $\epsilon \sim N(0, \sigma)$. In each scenario, subscenarios were considered according to the pairwise correlation of the predictors ("mixed", when selecting the predictors among the whole exposome, in which case the absolute pairwise correlation ranged from 0.0000 to 1.0000; or "high", when selecting the predictors among the subset of the 13 variables in the exposome for which all absolute pairwise correlations were 0.62 or higher); the size of the interaction terms ("strong", corresponding to equal size than the main effects size; or "moderate", corresponding to size 1/2 of the "strong"), and the sign of the interaction terms (+ or −). Values for the adjusted R^2 and sensitivities correspond to the mean and percentiles 2.5 and 97.5 as a result of fitting the model to 100 simulated datasets.

Subscenario	Adjusted R^2	Pairwise corr.	Interaction size (and sign)	Parameters			Sensitivity[a]	$\text{Sensitivity}_2^{\text{b}}$
Scenario 1. True model: $F(E) = \beta_0 + \beta_1 X_1 + \beta_2 X_2 + \beta_3 X_3 + \beta_4 X_4 + \beta_5 X_5$ (Size = 5; No interactions)								
1a	0.10 (0.07, 0.16)	mixed		$\beta_0 = \beta_1 = \cdots = \beta_5 = 1$		$\sigma = 7.5$	0.97 (0.70, 1.00)	
1b	0.30 (0.23, 0.39)	mixed		$\beta_0 = \beta_1 = \cdots = \beta_5 = 1$		$\sigma = 3.8$	0.99 (0.80, 1.00)	
1c	0.11 (0.09, 0.12)	high		$\beta_0 = \beta_1 = \cdots = \beta_5 = 1$		$\sigma = 13$	0.22 (0.00, 0.60)	
1d	0.27 (0.25, 0.28)	high		$\beta_0 = \beta_1 = \cdots = \beta_5 = 1$		$\sigma = 7.5$	0.40 (0.00, 0.80)	
Scenario 2. True model: $F(E) = \beta_0 + \beta_1 X_1 + \beta_2 X_2 + \beta_3 X_3 + \beta_4 X_4 + \beta_5 X_5 + \gamma_{12} X_1 X_2$ (Size = 6; Only one 2-way interaction)								
2a	0.09 (0.07, 0.14)	mixed	strong (+)	$\beta_0 = \beta_1 = \cdots = \beta_5 = 1$	$\gamma_{12} = 1$	$\sigma = 8.3$	0.96 (0.83, 1.00)	0.99 (1.00, 1.00)
2b	0.09 (0.06, 0.15)	mixed	strong (−)	$\beta_0 = \beta_1 = \cdots = \beta_5 = 1$	$\gamma_{12} = -1$	$\sigma = 8.3$	0.98 (0.83, 1.00)	1.00 (1.00, 1.00)
2c	0.10 (0.06, 0.15)	mixed	moderate (+)	$\beta_0 = \beta_1 = \cdots = \beta_5 = 1$	$\gamma_{12} = 0.5$	$\sigma = 7.8$	0.89 (0.67, 1.00)	0.55 (0.00, 1.00)
2d	0.10 (0.07, 0.14)	mixed	moderate (−)	$\beta_0 = \beta_1 = \cdots = \beta_5 = 1$	$\gamma_{12} = -0.5$	$\sigma = 7.8$	0.90 (0.67, 1.00)	0.55 (0.00, 1.00)
2e	0.13 (0.11, 0.14)	high	strong (+)	$\beta_0 = \beta_1 = \cdots = \beta_5 = 1$	$\gamma_{12} = 1$	$\sigma = 12$	0.59 (0.17, 0.83)	0.93 (0.00, 1.00)
2f	0.13 (0.11, 0.15)	high	strong (−)	$\beta_0 = \beta_1 = \cdots = \beta_5 = 1$	$\gamma_{12} = -1$	$\sigma = 12$	0.59 (0.17, 0.83)	0.96 (0.00, 1.00)
2g	0.30 (0.28, 0.32)	high	moderate (+)	$\beta_0 = \beta_1 = \cdots = \beta_5 = 1$	$\gamma_{12} = 0.5$	$\sigma = 7$	0.65 (0.17, 1.00)	0.84 (0.00, 1.00)
2h	0.30 (0.28, 0.32)	high	moderate (−)	$\beta_0 = \beta_1 = \cdots = \beta_5 = 1$	$\gamma_{12} = -0.5$	$\sigma = 7$	0.67 (0.00, 0.92)	0.86 (0.00, 1.00)
Scenario 3. True model: $F(E) = \beta_0 + \beta_1 X_1 + \beta_2 X_2 + \beta_3 X_3 + \beta_4 X_4 + \beta_5 X_5 + \gamma_{12} X_1 X_2 + \gamma_{13} X_1 X_3$ (Size = 7; X_1 involved in two 2-way interactions)								
3a	0.11 (0.08, 0.15)	mixed	strong (+)	$\beta_0 = \beta_1 = \cdots \beta_5 = 1$	$\gamma_{12} = \gamma_{13} = 1$	$\sigma = 8.3$	0.98 (0.86, 1.00)	0.99 (1.00, 1.00)
3b	0.10 (0.08, 0.16)	mixed	strong (−)	$\beta_0 = \beta_1 = \cdots = \beta_5 = 1$	$\gamma_{12} = \gamma_{13} = -1$	$\sigma = 8.3$	0.97 (0.86, 1.00)	0.97 (0.50, 1.00)
3c	0.10 (0.06, 0.14)	mixed	moderate (+)	$\beta_0 = \beta_1 = \cdots = \beta_5 = 1$	$\gamma_{12} = \gamma_{13} = 0.5$	$\sigma = 7.8$	0.86 (0.57, 1.00)	0.61 (0.00, 1.00)
3d	0.10 (0.07, 0.14)	mixed	moderate (−)	$\beta_0 = \beta_1 = \cdots = \beta_5 = 1$	$\gamma_{12} = \gamma_{13} = -0.5$	$\sigma = 7.8$	0.87 (0.64, 1.00)	0.59 (0.00, 1.00)
3e	0.29 (0.27, 0.32)	high	strong (+)	$\beta_0 = \beta_1 = \cdots = \beta_5 = 1$	$\gamma_{12} = \gamma_{13} = 1$	$\sigma = 8$	0.68 (0.21, 1.00)	0.76 (0.00, 1.00)
3f	0.29 (0.27, 0.31)	high	strong (−)	$\beta_0 = \beta_1 = \cdots = \beta_5 = 1$	$\gamma_{12} = \gamma_{13} = -1$	$\sigma = 8$	0.64 (0.14, 1.00)	0.69 (0.00, 1.00)
3g	0.31 (0.29, 0.33)	high	moderate (+)	$\beta_0 = \beta_1 = \cdots = \beta_5 = 1$	$\gamma_{12} = \gamma_{13} = 0.5$	$\sigma = 7$	0.50 (0.07, 0.86)	0.35 (0.00, 1.00)
3h	0.31 (0.28, 0.33)	high	moderate (−)	$\beta_0 = \beta_1 = \cdots = \beta_5 = 1$	$\gamma_{12} = \gamma_{13} = -0.5$	$\sigma = 7$	0.50 (0.14, 0.93)	0.32 (0.00, 1.00)

a: Proportion of true terms detected by the fitted model. In models with interaction terms, the main effects of variables involved in true interactions detected are assumed to be also detected.
b: Proportion of true interaction terms detected by the fitted model.

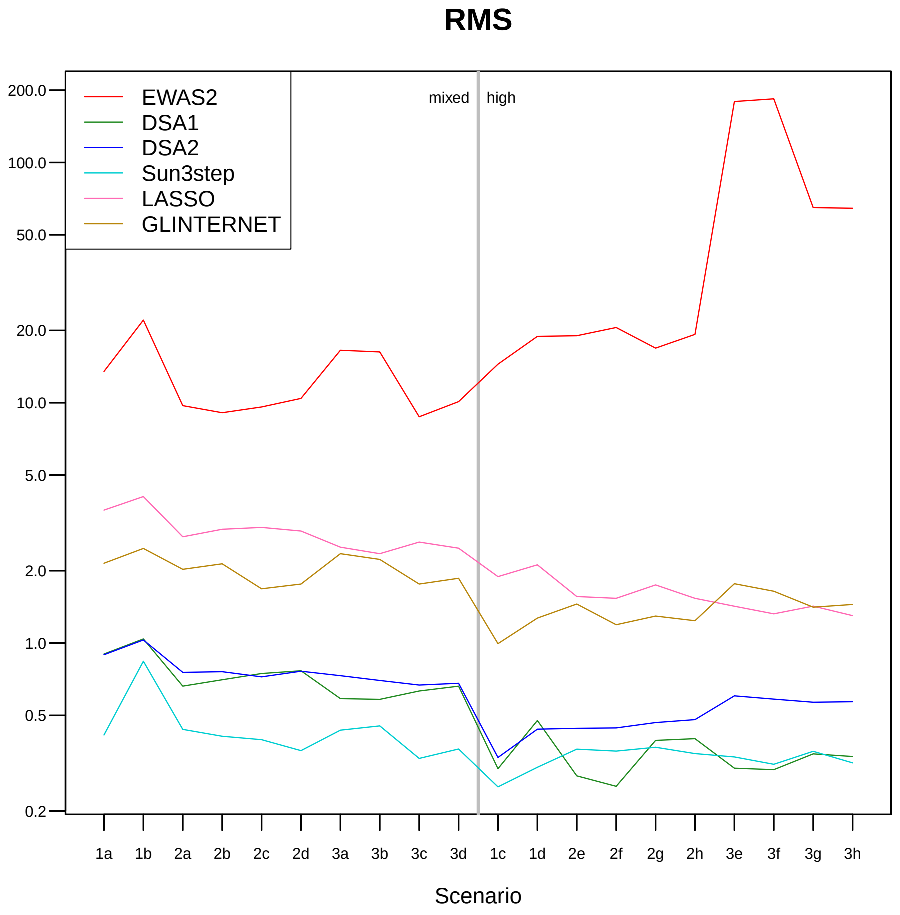

Figure 3: Performance of the compared methods in terms of relative model size (RMS), in log scale. The measure is relative such that the true model corresponds to the value 1. Mean values based on 100 simulations. The vertical line separates scenarios according to the pairwise correlation between the true predictors as "mixed" (any exposure can be selected as a true predictor regardless of correlation), or "high" (exposures are choosen so that all their pairwise correlations are above 0.6). Scenarios 1, 2 and 3 involve no interactions, one two-way interaction, and two two-way interactions, respectively.

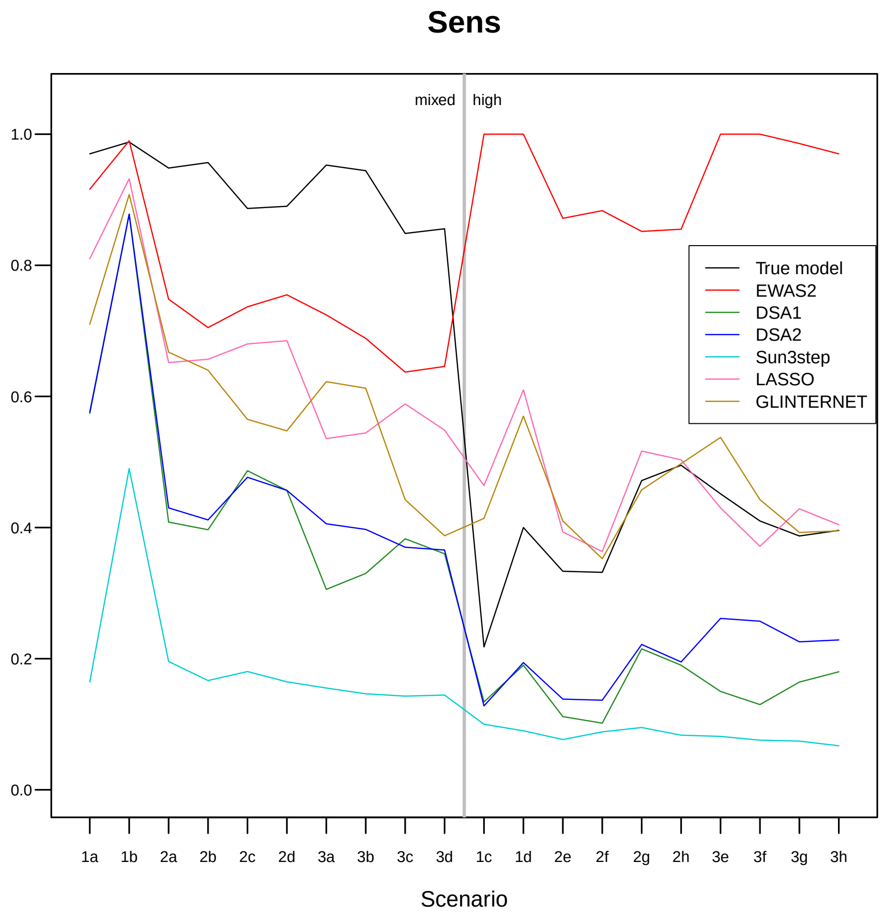

Figure 4: Performance of the compared methods in sensitivity of terms (Sens). Mean values based on 100 simulations. The vertical line separates scenarios according to the pairwise correlation between the true predictors as "mixed" (any exposure can be selected as a true predictor regardless of correlation), or "high" (exposures are choosen so that all their pairwise correlations are above 0.6). Scenarios 1, 2 and 3 involve no interactions, one two-way interaction, and two two-way interactions, respectively.

F Performance in sensitivity for terms (Sens)

See Figure 4.

G Performance in false discevory proportion of terms (FDP)

See Figure 5.

H Numerical results on the performance of the models in all scenarios

See Tables 2 to 21.

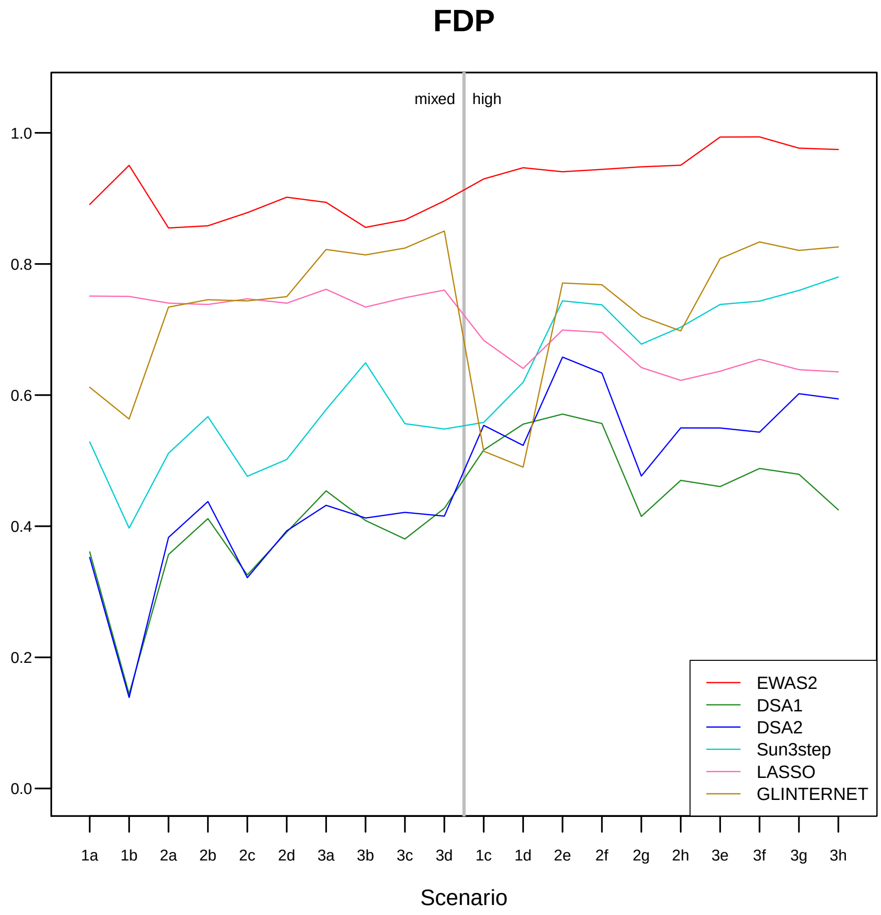

Figure 5: Performance of the compared methods in false discevory proportion of terms (FDP). Mean values based on 100 simulations. The vertical line separates scenarios according to the pairwise correlation between the true predictors as "mixed" (any exposure can be selected as a true predictor regardless of correlation), or "high" (exposures are choosen so that all their pairwise correlations are above 0.6). Scenarios 1, 2 and 3 involve no interactions, one two-way interaction, and two two-way interactions, respectively.

I Performance in AltSens_2

See Figure 26.

J Performance in AltFDP_2

See Figure 27.

K Trade-off between sensitivity and specificity

See Figures 6 to 25.

	True model			$\mathbf{EWAS_2}$			$\mathbf{DSA_1}$			$\mathbf{DSA_2}$			Sun3step			LASSO			GLINTERNET			BRT		
	Mean	2.5%	97.5%	Mean	2.5%	97.5%	Mean	2.5%	97.5%	Mean	2.5%	97.5%	Mean	2.5%	97.5%	Mean	2.5%	97.5%	Mean	2.5%	97.5%	Mean	2.5%	97.5%
RMS				13.50	1.70	23.00	0.90	0.40	1.50	0.89	0.40	1.70	0.41	0.20	1.18	3.57	1.70	5.70	2.15	0.60	6.31			
RNV				13.50	1.70	23.00	0.90	0.40	1.50	0.89	0.40	1.60	0.33	0.20	0.60	3.57	1.70	5.70	1.94	0.60	4.30	1.12	0.40	3.00
R^2_{rel}							0.84	0.57	1.00	0.84	0.55	1.00	0.50	0.22	0.77	0.88	0.74	0.97	0.82	0.58	0.96	0.28	0.11	0.50
Sens	0.97	0.70	1.00	0.92	0.60	1.00	0.57	0.20	1.00	0.58	0.10	1.00	0.16	0.00	0.40	0.81	0.40	1.00	0.71	0.20	1.00			
AltSens				0.94	0.65	1.00	0.84	0.57	1.00	0.84	0.45	1.00	0.44	0.22	0.67	0.97	0.84	1.00	0.91	0.61	1.00	0.72	0.46	1.00
Sensvar	0.97	0.70	1.00	0.92	0.60	1.00	0.57	0.20	1.00	0.58	0.10	1.00	0.16	0.00	0.40	0.81	0.40	1.00	0.71	0.20	1.00	0.49	0.20	0.90
Sens_2																								
AltSens_2				0.00	0.00	0.00																		
FDP				0.89	0.58	0.96	0.36	0.00	0.78	0.35	0.00	0.89	0.53	0.00	1.00	0.75	0.55	0.89	0.61	0.31	0.94			
AltFDP				0.40	0.15	0.54	0.12	0.00	0.40	0.11	0.00	0.36	0.12	0.00	0.46	0.42	0.19	0.62	0.22	0.02	0.50	0.21	0.00	0.57
FDPvar				0.89	0.58	0.96	0.36	0.00	0.78	0.35	0.00	0.88	0.47	0.00	1.00	0.75	0.55	0.89	0.60	0.31	0.92	0.48	0.00	0.90
FDP_2										0.03	0.00	0.52	0.29	0.00	1.00				0.35	0.00	1.00			
AltFDP_2				0.00	0.00	0.00				0.00	0.00	0.00	0.00	0.00	0.00				0.00	0.00	0.00			

Table 2: Performance in scenario 1a.

	True model			$\mathbf{EWAS_2}$			$\mathbf{DSA_1}$			$\mathbf{DSA_2}$			Sun3step			LASSO			GLINTERNET			BRT		
	Mean	2.5%	97.5%	Mean	2.5%	97.5%	Mean	2.5%	97.5%	Mean	2.5%	97.5%	Mean	2.5%	97.5%	Mean	2.5%	97.5%	Mean	2.5%	97.5%	Mean	2.5%	97.5%
RMS				22.09	8.88	29.70	1.04	0.80	1.60	1.03	0.80	1.30	0.84	0.20	1.70	4.07	2.30	6.60	2.48	1.20	4.82			
RNV				22.09	8.88	29.70	1.04	0.80	1.60	1.03	0.80	1.30	0.72	0.20	1.00	4.07	2.30	6.60	2.26	1.20	4.01	1.57	0.80	3.00
R^2_{rel}							0.99	0.94	1.00	0.99	0.94	1.00	0.76	0.38	1.00	0.97	0.94	1.00	0.97	0.92	1.00	0.68	0.47	0.81
Sens	0.99	0.80	1.00	0.99	0.80	1.00	0.88	0.60	1.00	0.88	0.60	1.00	0.49	0.00	1.00	0.93	0.70	1.00	0.91	0.60	1.00			
AltSens				0.99	0.86	1.00	0.99	0.92	1.00	0.99	0.93	1.00	0.73	0.31	1.00	1.00	0.97	1.00	1.00	0.98	1.00	0.96	0.80	1.00
Sensvar	0.99	0.80	1.00	0.99	0.80	1.00	0.88	0.60	1.00	0.88	0.60	1.00	0.49	0.00	1.00	0.93	0.70	1.00	0.91	0.60	1.00	0.83	0.40	1.00
Sens_2																								
AltSens_2				0.00	0.00	0.00																		
FDP				0.95	0.90	0.97	0.14	0.00	0.60	0.14	0.00	0.50	0.40	0.00	1.00	0.75	0.56	0.87	0.56	0.17	0.86			
AltFDP				0.53	0.43	0.64	0.03	0.00	0.27	0.03	0.00	0.20	0.09	0.00	0.33	0.46	0.24	0.60	0.23	0.05	0.54	0.09	0.00	0.25
FDPvar				0.95	0.90	0.97	0.14	0.00	0.60	0.14	0.00	0.50	0.33	0.00	1.00	0.75	0.56	0.87	0.55	0.17	0.82	0.41	0.00	0.73
FDP_2										0.01	0.00	0.00	0.37	0.00	1.00				0.36	0.00	1.00			
AltFDP_2				0.00	0.00	0.00				0.00	0.00	0.00	0.00	0.00	0.00				0.00	0.00	0.00			

Table 3: Performance in scenario 1b.

	**True model**			**$EWAS_2$**			**DSA_1**			**DSA_2**			**Sun3step**			**LASSO**			**GLINTERNET**			**BRT**		
	Mean	2.5%	97.5%	Mean	2.5%	97.5%	Mean	2.5%	97.5%	Mean	2.5%	97.5%	Mean	2.5%	97.5%	Mean	2.5%	97.5%	Mean	2.5%	97.5%	Mean	2.5%	97.5%
RMS				14.47	10.76	17.91	0.30	0.20	1.00	0.33	0.20	0.80	0.25	0.20	0.50	1.89	0.50	4.70	0.99	0.40	2.41			
RNV				14.47	10.76	17.91	0.30	0.20	1.00	0.32	0.20	0.80	0.21	0.20	0.30	1.89	0.50	4.70	0.95	0.40	2.10	0.62	0.20	3.00
R^2_{rel}							0.93	0.81	1.03	0.93	0.80	1.01	0.93	0.82	1.02	0.98	0.91	1.04	1.00	0.95	1.04	0.46	0.30	0.62
Sens	0.22	0.00	0.60	1.00	1.00	1.00	0.13	0.00	0.40	0.13	0.00	0.40	0.10	0.00	0.20	0.46	0.00	0.80	0.41	0.00	0.80			
AltSens				1.00	1.00	1.00	0.87	0.81	0.94	0.87	0.81	0.94	0.86	0.81	0.92	0.96	0.89	1.00	0.95	0.89	1.00	0.88	0.77	0.99
Sensvar	0.22	0.00	0.60	1.00	1.00	1.00	0.13	0.00	0.40	0.13	0.00	0.40	0.10	0.00	0.20	0.46	0.00	0.80	0.41	0.00	0.80	0.24	0.00	0.80
Sens_2																								
AltSens_2				0.00	0.00	0.00																		
FDP				0.93	0.91	0.94	0.52	0.00	1.00	0.55	0.00	1.00	0.56	0.00	1.00	0.68	0.25	1.00	0.51	0.00	1.00			
AltFDP				0.44	0.37	0.50	0.11	0.00	0.62	0.14	0.00	0.66	0.04	0.00	0.14	0.33	0.00	0.68	0.11	0.00	0.45	0.10	0.00	0.45
FDPvar				0.93	0.91	0.94	0.52	0.00	1.00	0.55	0.00	1.00	0.51	0.00	1.00	0.68	0.25	1.00	0.51	0.00	1.00	0.49	0.00	1.00
FDP_2										0.05	0.00	1.00	0.23	0.00	1.00				0.11	0.00	1.00			
AltFDP_2				0.00	0.00	0.00				0.00	0.00	0.00	0.00	0.00	0.00				0.00	0.00	0.00			

Table 4: Performance in scenario 1c.

	True model			EWAS$_2$			DSA$_1$			DSA$_2$			Sun3step			LASSO			GLINTERNET			BRT		
	Mean	2.5%	97.5%	Mean	2.5%	97.5%	Mean	2.5%	97.5%	Mean	2.5%	97.5%	Mean	2.5%	97.5%	Mean	2.5%	97.5%	Mean	2.5%	97.5%	Mean	2.5%	97.5%
RMS				18.89	16.59	22.10	0.48	0.20	1.00	0.44	0.20	1.00	0.30	0.20	1.00	2.12	0.80	5.32	1.27	0.50	2.20			
RNV				18.89	16.59	22.10	0.48	0.20	1.00	0.43	0.20	0.80	0.24	0.20	0.40	2.12	0.80	5.32	1.22	0.50	2.10	0.99	0.20	3.00
R^2_{rel}							0.97	0.92	1.01	0.97	0.91	1.01	0.94	0.86	1.00	0.99	0.97	1.01	1.00	0.98	1.01	0.71	0.62	0.81
Sens	0.40	0.00	0.80	1.00	1.00	1.00	0.19	0.00	0.40	0.19	0.00	0.40	0.09	0.00	0.20	0.61	0.20	1.00	0.57	0.20	1.00			
AltSens				1.00	1.00	1.00	0.91	0.85	0.96	0.91	0.86	0.97	0.88	0.83	0.93	0.98	0.95	1.00	0.98	0.93	1.00	0.93	0.86	1.00
Sensvar	0.40	0.00	0.80	1.00	1.00	1.00	0.19	0.00	0.40	0.19	0.00	0.40	0.09	0.00	0.20	0.61	0.20	1.00	0.57	0.20	1.00	0.40	0.00	1.00
Sens$_2$																								
AltSens$_2$				0.00	0.00	0.00																		
FDP				0.95	0.94	0.95	0.56	0.00	1.00	0.52	0.00	1.00	0.62	0.00	1.00	0.64	0.20	0.92	0.49	0.00	0.90			
AltFDP				0.52	0.45	0.57	0.13	0.00	0.53	0.08	0.00	0.52	0.08	0.00	0.49	0.31	0.00	0.73	0.10	0.00	0.40	0.08	0.00	0.43
FDPvar				0.95	0.94	0.95	0.56	0.00	1.00	0.52	0.00	1.00	0.59	0.00	1.00	0.64	0.20	0.92	0.49	0.00	0.88	0.50	0.00	1.00
FDP$_2$										0.04	0.00	1.00	0.21	0.00	1.00				0.09	0.00	1.00			
AltFDP$_2$				0.00	0.00	0.00				0.00	0.00	0.00	0.00	0.00	0.00				0.00	0.00	0.00			

Table 5: Performance in scenario 1d.

	**True model**			**EWAS$_2$**			**DSA$_1$**			**DSA$_2$**			**Sun3step**			**LASSO**			**GLINTERNET**			**BRT**		
	Mean	2.5%	97.5%	Mean	2.5%	97.5%	Mean	2.5%	97.5%	Mean	2.5%	97.5%	Mean	2.5%	97.5%	Mean	2.5%	97.5%	Mean	2.5%	97.5%	Mean	2.5%	97.5%
RMS				9.73	0.75	23.91	0.66	0.25	1.25	0.76	0.33	1.67	0.44	0.17	1.38	2.77	1.33	4.83	2.03	0.75	4.34			
RNV				10.64	0.90	21.01	0.79	0.30	1.50	0.84	0.40	1.80	0.36	0.20	0.80	3.32	1.60	5.80	1.95	0.80	3.70	0.72	0.40	1.40
R^2_{rel}							0.66	0.38	0.87	0.65	0.35	0.90	0.51	0.22	0.83	0.73	0.54	0.86	0.77	0.45	0.95	0.33	0.07	0.59
Sens	0.95	0.67	1.00	0.75	0.25	1.00	0.41	0.00	0.67	0.43	0.00	0.83	0.20	0.00	0.50	0.65	0.33	0.83	0.67	0.25	1.00			
AltSens				0.88	0.38	1.00	0.76	0.40	1.00	0.77	0.40	1.00	0.48	0.23	0.82	0.96	0.76	1.00	0.91	0.54	1.00	0.61	0.29	0.87
Sensvar	0.96	0.80	1.00	0.85	0.30	1.00	0.49	0.00	0.80	0.50	0.00	1.00	0.22	0.00	0.60	0.78	0.40	1.00	0.74	0.30	1.00	0.38	0.00	0.80
Sens$_2$	0.99	1.00	1.00	0.24	0.00	1.00				0.08	0.00	1.00	0.07	0.00	1.00				0.69	0.00	1.00			
AltSens$_2$				0.25	0.00	1.00				0.71	0.00	1.00	0.59	0.00	1.00				0.95	0.30	1.00			
FDP				0.85	0.39	0.97	0.36	0.00	0.93	0.38	0.00	0.93	0.51	0.00	1.00	0.74	0.52	0.91	0.73	0.46	0.96			
AltFDP				0.35	0.05	0.52	0.12	0.00	0.44	0.13	0.00	0.43	0.10	0.00	0.52	0.43	0.20	0.64	0.22	0.01	0.46	0.12	0.00	0.38
FDPvar				0.85	0.41	0.96	0.36	0.00	0.93	0.37	0.00	0.93	0.38	0.00	1.00	0.74	0.52	0.91	0.56	0.18	0.83	0.42	0.00	1.00
FDP$_2$				0.72	0.00	1.00				0.18	0.00	1.00	0.48	0.00	1.00				0.49	0.00	1.00			
AltFDP$_2$				0.05	0.00	0.32				0.09	0.00	0.97	0.18	0.00	0.91				0.23	0.00	0.84			

Table 6: Performance in scenario 2a.

	True model			$EWAS_2$			DSA_1			DSA_2			Sun3step			LASSO			GLINTERNET			BRT		
	Mean	2.5%	97.5%	Mean	2.5%	97.5%	Mean	2.5%	97.5%	Mean	2.5%	97.5%	Mean	2.5%	97.5%	Mean	2.5%	97.5%	Mean	2.5%	97.5%	Mean	2.5%	97.5%
RMS				9.09	0.41	18.51	0.70	0.17	1.33	0.76	0.17	1.42	0.41	0.17	1.01	2.98	1.17	5.67	2.14	0.67	5.35			
RNV				10.01	0.50	20.10	0.84	0.20	1.60	0.85	0.20	1.60	0.34	0.20	0.60	3.57	1.40	6.80	2.03	0.70	4.40	0.71	0.20	1.40
R^2_{rel}							0.62	0.08	0.85	0.62	0.15	0.85	0.45	0.10	0.82	0.70	0.49	0.87	0.73	0.43	0.95	0.32	0.04	0.63
Sens	0.96	0.75	1.00	0.70	0.17	1.00	0.40	0.00	0.75	0.41	0.00	0.75	0.17	0.00	0.50	0.66	0.33	0.83	0.64	0.00	1.00			
AltSens				0.83	0.20	1.00	0.73	0.19	1.00	0.73	0.22	1.00	0.42	0.11	0.80	0.96	0.81	1.00	0.89	0.55	1.00	0.59	0.20	0.87
Sensvar	0.97	0.80	1.00	0.79	0.20	1.00	0.48	0.00	0.90	0.47	0.00	0.80	0.18	0.00	0.40	0.79	0.40	1.00	0.73	0.20	1.00	0.38	0.00	0.80
$Sens_2$	1.00	1.00	1.00	0.26	0.00	1.00				0.10	0.00	1.00	0.09	0.00	1.00				0.65	0.00	1.00			
$AltSens_2$				0.27	0.00	1.00				0.75	0.00	1.00	0.59	0.04	1.00				0.95	0.14	1.00			
FDP				0.86	0.50	0.96	0.41	0.00	1.00	0.44	0.00	1.00	0.57	0.00	1.00	0.74	0.41	0.90	0.75	0.43	1.00			
AltFDP				0.36	0.03	0.54	0.17	0.00	0.56	0.17	0.00	0.54	0.15	0.00	0.68	0.45	0.20	0.65	0.24	0.01	0.56	0.15	0.00	0.54
FDPvar				0.85	0.37	0.96	0.41	0.00	1.00	0.42	0.00	1.00	0.47	0.00	1.00	0.74	0.41	0.90	0.57	0.17	0.92	0.41	0.00	1.00
FDP_2				0.81	0.34	1.00				0.19	0.00	1.00	0.46	0.00	1.00				0.49	0.00	1.00			
$AltFDP_2$				0.04	0.00	0.35				0.06	0.00	0.89	0.18	0.00	0.93				0.21	0.00	0.84			

Table 7: Performance in scenario 2b.

	True model			$\mathbf{EWAS_2}$			$\mathbf{DSA_1}$			$\mathbf{DSA_2}$			Sun3step			LASSO			GLINTERNET			BRT		
	Mean	2.5%	97.5%	Mean	2.5%	97.5%	Mean	2.5%	97.5%	Mean	2.5%	97.5%	Mean	2.5%	97.5%	Mean	2.5%	97.5%	Mean	2.5%	97.5%	Mean	2.5%	97.5%
RMS				9.61	1.33	18.94	0.75	0.33	1.50	0.72	0.33	1.34	0.40	0.17	0.83	3.03	1.41	5.50	1.68	0.75	3.94			
RNV				11.53	1.59	22.72	0.90	0.40	1.80	0.86	0.40	1.61	0.34	0.20	0.60	3.63	1.70	6.60	1.84	0.80	3.61	0.75	0.40	1.40
R^2_{rel}							0.79	0.37	0.98	0.79	0.37	0.97	0.50	0.16	0.83	0.83	0.67	0.95	0.78	0.53	0.96	0.38	0.10	0.68
Sens	0.89	0.67	1.00	0.74	0.41	0.83	0.49	0.08	0.83	0.48	0.00	0.83	0.18	0.00	0.50	0.68	0.33	0.83	0.56	0.12	0.88			
AltSens				0.91	0.61	1.00	0.82	0.38	1.00	0.81	0.38	1.00	0.45	0.20	0.76	0.97	0.81	1.00	0.90	0.62	1.00	0.63	0.35	0.91
Sensvar	0.96	0.70	1.00	0.88	0.50	1.00	0.58	0.10	1.00	0.57	0.00	1.00	0.21	0.00	0.58	0.82	0.40	1.00	0.72	0.30	1.00	0.45	0.00	0.80
Sens_2	0.55	0.00	1.00	0.00	0.00	0.00				0.00	0.00	0.00	0.04	0.00	0.90				0.32	0.00	1.00			
$\mathrm{AltSens}_2$				0.00	0.00	0.00				0.56	0.39	0.73	0.53	0.00	1.00				0.68	0.10	1.00			
FDP				0.88	0.61	0.96	0.33	0.00	0.95	0.32	0.00	1.00	0.48	0.00	1.00	0.75	0.52	0.91	0.74	0.50	0.97			
AltFDP				0.39	0.21	0.54	0.12	0.00	0.54	0.12	0.00	0.48	0.10	0.00	0.42	0.45	0.20	0.64	0.22	0.02	0.46	0.13	0.00	0.54
FDPvar				0.88	0.61	0.96	0.33	0.00	0.95	0.32	0.00	1.00	0.38	0.00	1.00	0.75	0.52	0.91	0.56	0.20	0.84	0.38	0.00	1.00
FDP_2										0.02	0.00	0.00	0.40	0.00	1.00				0.31	0.00	1.00			
AltFDP_2				0.00	0.00	0.00				0.01	0.00	0.00	0.14	0.00	0.85				0.19	0.00	0.88			

Table 8: Performance in scenario 2c.

	True model			$EWAS_2$			DSA_1			DSA_2			Sun3step			LASSO			GLINTERNET			BRT		
	Mean	2.5%	97.5%	Mean	2.5%	97.5%	Mean	2.5%	97.5%	Mean	2.5%	97.5%	Mean	2.5%	97.5%	Mean	2.5%	97.5%	Mean	2.5%	97.5%	Mean	2.5%	97.5%
RMS				10.43	2.07	17.87	0.77	0.33	1.25	0.76	0.33	1.17	0.36	0.17	0.98	2.92	1.58	4.59	1.76	0.67	4.17			
RNV				12.51	2.49	21.45	0.92	0.40	1.50	0.91	0.40	1.40	0.32	0.20	0.60	3.51	1.90	5.50	1.89	0.80	3.81	0.80	0.40	2.24
R^2_{rel}							0.79	0.46	0.98	0.79	0.47	0.98	0.48	0.16	0.81	0.84	0.69	0.95	0.79	0.47	0.96	0.38	0.16	0.60
Sens	0.89	0.67	1.00	0.76	0.50	0.83	0.46	0.00	0.83	0.46	0.00	0.83	0.16	0.00	0.50	0.68	0.33	0.83	0.55	0.12	1.00			
AltSens				0.93	0.62	1.00	0.82	0.50	1.00	0.82	0.50	1.00	0.42	0.18	0.77	0.97	0.85	1.00	0.90	0.61	1.00	0.64	0.42	0.86
Sensvar	0.97	0.80	1.00	0.91	0.60	1.00	0.55	0.00	1.00	0.55	0.00	1.00	0.19	0.00	0.60	0.82	0.40	1.00	0.70	0.20	1.00	0.42	0.20	0.80
$Sens_2$	0.55	0.00	1.00	0.00	0.00	0.00				0.00	0.00	0.00	0.03	0.00	0.88				0.24	0.00	1.00			
$AltSens_2$				0.00	0.00	0.00				0.42	0.16	0.69	0.46	0.00	1.00				0.65	0.08	1.00			
FDP				0.90	0.74	0.96	0.39	0.00	1.00	0.39	0.00	1.00	0.50	0.00	1.00	0.74	0.50	0.89	0.75	0.40	0.96			
AltFDP				0.40	0.21	0.52	0.15	0.00	0.50	0.14	0.00	0.40	0.12	0.00	0.54	0.43	0.20	0.65	0.22	0.00	0.48	0.12	0.00	0.43
FDPvar				0.90	0.74	0.96	0.39	0.00	1.00	0.39	0.00	1.00	0.42	0.00	1.00	0.74	0.50	0.89	0.57	0.00	0.87	0.39	0.00	0.87
FDP_2				1.00	1.00	1.00				0.05	0.00	1.00	0.39	0.00	1.00				0.37	0.00	1.00			
$AltFDP_2$				0.01	0.00	0.00				0.01	0.00	0.00	0.16	0.00	0.86				0.22	0.00	0.93			

Table 9: Performance in scenario 2d.

	True model			$\mathbf{EWAS_2}$			$\mathbf{DSA_1}$			$\mathbf{DSA_2}$			Sun3step			LASSO			GLINTERNET			BRT		
	Mean	2.5%	97.5%	Mean	2.5%	97.5%	Mean	2.5%	97.5%	Mean	2.5%	97.5%	Mean	2.5%	97.5%	Mean	2.5%	97.5%	Mean	2.5%	97.5%	Mean	2.5%	97.5%
RMS				19.01	9.33	80.73	0.28	0.17	0.67	0.44	0.17	1.09	0.36	0.17	0.67	1.56	0.50	3.25	1.45	0.50	4.36			
RNV				15.39	11.20	18.40	0.34	0.20	0.80	0.43	0.20	1.20	0.23	0.20	0.40	1.87	0.60	3.90	1.38	0.60	3.42	0.40	0.20	1.11
R^2_{rel}							0.87	0.73	0.96	0.90	0.76	1.00	0.92	0.77	1.01	0.92	0.85	0.97	0.95	0.90	1.02	0.67	0.54	0.76
Sens	0.33	0.17	0.67	0.87	0.83	1.00	0.11	0.00	0.33	0.14	0.00	0.33	0.08	0.00	0.17	0.39	0.08	0.67	0.41	0.00	0.75			
AltSens				1.00	1.00	1.00	0.88	0.81	0.95	0.89	0.83	0.96	0.87	0.80	0.92	0.96	0.92	1.00	0.97	0.91	1.00	0.89	0.79	0.96
Sensvar	0.52	0.20	0.80	1.00	1.00	1.00	0.13	0.00	0.40	0.16	0.00	0.40	0.09	0.00	0.20	0.47	0.10	0.80	0.52	0.10	0.80	0.21	0.00	0.60
Sens_2	0.93	0.00	1.00	0.23	0.00	1.00				0.01	0.00	0.00	0.00	0.00	0.00				0.24	0.00	1.00			
$\mathrm{AltSens}_2$				0.25	0.00	1.00				0.85	0.30	1.00	0.87	0.70	0.96				0.93	0.62	1.00			
FDP				0.94	0.91	0.99	0.57	0.00	1.00	0.66	0.00	1.00	0.74	0.00	1.00	0.70	0.33	0.97	0.77	0.50	1.00			
AltFDP				0.45	0.38	0.53	0.11	0.00	0.57	0.15	0.00	0.60	0.06	0.00	0.45	0.34	0.01	0.67	0.15	0.00	0.52	0.03	0.00	0.16
FDPvar				0.93	0.91	0.95	0.57	0.00	1.00	0.59	0.00	1.00	0.57	0.00	1.00	0.70	0.33	0.97	0.57	0.25	0.96	0.45	0.00	1.00
FDP_2				0.96	0.80	1.00				0.50	0.00	1.00	0.88	0.00	1.00				0.60	0.00	1.00			
AltFDP_2				0.07	0.00	0.40				0.05	0.00	0.47	0.09	0.00	0.45				0.12	0.00	0.64			

Table 10: Performance in scenario 2e.

	True model			EWAS$_2$			DSA$_1$			DSA$_2$			Sun3step			LASSO			GLINTERNET			BRT		
	Mean	2.5%	97.5%	Mean	2.5%	97.5%	Mean	2.5%	97.5%	Mean	2.5%	97.5%	Mean	2.5%	97.5%	Mean	2.5%	97.5%	Mean	2.5%	97.5%	Mean	2.5%	97.5%
RMS				20.57	10.41	64.53	0.25	0.17	0.59	0.44	0.17	1.00	0.36	0.25	0.67	1.53	0.50	3.92	1.19	0.33	2.51			
RNV				15.01	11.99	17.60	0.30	0.20	0.70	0.41	0.20	0.90	0.22	0.20	0.40	1.84	0.60	4.70	1.13	0.40	2.00	0.39	0.20	1.11
R^2_{rel}							0.89	0.81	0.96	0.90	0.77	0.99	0.93	0.76	1.01	0.92	0.86	0.97	0.95	0.90	1.02	0.69	0.53	0.81
Sens	0.33	0.17	0.67	0.88	0.83	1.00	0.10	0.00	0.33	0.14	0.00	0.33	0.09	0.00	0.17	0.36	0.00	0.67	0.35	0.00	0.75			
AltSens				1.00	1.00	1.00	0.89	0.83	0.96	0.90	0.83	0.96	0.87	0.79	0.92	0.96	0.90	1.00	0.96	0.90	1.00	0.89	0.80	0.98
Sensvar	0.51	0.20	0.80	1.00	1.00	1.00	0.12	0.00	0.40	0.16	0.00	0.40	0.11	0.00	0.20	0.44	0.00	0.80	0.47	0.00	0.90	0.19	0.00	0.60
Sens$_2$	0.96	0.00	1.00	0.30	0.00	1.00				0.01	0.00	0.00	0.00	0.00	0.00				0.12	0.00	1.00			
AltSens$_2$				0.36	0.00	1.00				0.87	0.30	1.00	0.87	0.75	0.99				0.94	0.77	1.00			
FDP				0.94	0.92	0.98	0.56	0.00	1.00	0.63	0.00	1.00	0.74	0.24	1.00	0.70	0.33	1.00	0.77	0.45	1.00			
AltFDP				0.44	0.37	0.51	0.09	0.00	0.45	0.13	0.00	0.53	0.04	0.00	0.45	0.33	0.01	0.72	0.11	0.00	0.37	0.04	0.00	0.17
FDPvar				0.93	0.92	0.94	0.56	0.00	1.00	0.57	0.00	1.00	0.51	0.00	1.00	0.70	0.33	1.00	0.55	0.00	1.00	0.48	0.00	1.00
FDP$_2$				0.99	0.96	1.00				0.54	0.00	1.00	0.97	0.48	1.00				0.66	0.00	1.00			
AltFDP$_2$				0.11	0.00	0.42				0.06	0.00	0.55	0.09	0.00	0.40				0.08	0.00	0.33			

Table 11: Performance in scenario 2f.

	True model			EWAS$_2$			DSA$_1$			DSA$_2$			Sun3step			LASSO			GLINTERNET			BRT		
	Mean	2.5%	97.5%	Mean	2.5%	97.5%	Mean	2.5%	97.5%	Mean	2.5%	97.5%	Mean	2.5%	97.5%	Mean	2.5%	97.5%	Mean	2.5%	97.5%	Mean	2.5%	97.5%
RMS				16.88	13.50	30.01	0.39	0.17	0.83	0.47	0.17	1.00	0.37	0.17	1.17	1.74	0.58	3.75	1.29	0.50	3.45			
RNV				18.92	16.20	22.02	0.47	0.20	1.00	0.49	0.20	1.00	0.24	0.20	0.60	2.09	0.70	4.50	1.32	0.60	3.02	0.43	0.20	0.80
R^2_{rel}							0.96	0.90	0.99	0.96	0.92	1.00	0.93	0.85	0.99	0.98	0.95	0.99	0.98	0.96	1.00	0.85	0.79	0.91
Sens	0.47	0.17	0.83	0.85	0.83	1.00	0.22	0.00	0.50	0.22	0.00	0.50	0.10	0.00	0.17	0.52	0.17	0.83	0.46	0.25	0.75			
AltSens				1.00	1.00	1.00	0.92	0.84	0.99	0.92	0.85	0.98	0.87	0.81	0.93	0.98	0.94	1.00	0.98	0.94	1.00	0.92	0.86	0.97
Sensvar	0.61	0.20	1.00	1.00	1.00	1.00	0.26	0.00	0.60	0.27	0.00	0.60	0.11	0.00	0.20	0.62	0.20	1.00	0.58	0.20	1.00	0.27	0.00	0.60
Sens$_2$	0.84	0.00	1.00	0.11	0.00	1.00				0.00	0.00	0.00	0.00	0.00	0.00				0.06	0.00	1.00			
AltSens$_2$				0.14	0.00	1.00				0.88	0.55	1.00	0.88	0.76	1.00				0.90	0.35	1.00			
FDP				0.95	0.94	0.97	0.41	0.00	1.00	0.48	0.00	1.00	0.68	0.00	1.00	0.64	0.31	0.89	0.72	0.50	0.95			
AltFDP				0.52	0.47	0.58	0.10	0.00	0.51	0.09	0.00	0.50	0.06	0.00	0.49	0.31	0.01	0.68	0.13	0.00	0.42	0.02	0.00	0.08
FDPvar				0.95	0.94	0.95	0.41	0.00	1.00	0.42	0.00	1.00	0.46	0.00	1.00	0.64	0.31	0.89	0.51	0.10	0.83	0.36	0.00	1.00
FDP$_2$				0.97	0.90	1.00				0.34	0.00	1.00	0.84	0.00	1.00				0.50	0.00	1.00			
AltFDP$_2$				0.03	0.00	0.35				0.04	0.00	0.45	0.09	0.00	0.58				0.11	0.00	0.74			

Table 12: Performance in scenario 2g.

	True model			$EWAS_2$			DSA_1			DSA_2			Sun3step			LASSO			GLINTERNET			BRT		
	Mean	2.5%	97.5%	Mean	2.5%	97.5%	Mean	2.5%	97.5%	Mean	2.5%	97.5%	Mean	2.5%	97.5%	Mean	2.5%	97.5%	Mean	2.5%	97.5%	Mean	2.5%	97.5%
RMS				19.25	14.33	58.94	0.40	0.17	0.83	0.48	0.17	1.00	0.35	0.17	0.75	1.53	0.67	3.50	1.24	0.50	3.10			
RNV				19.00	17.20	21.51	0.48	0.20	1.00	0.50	0.20	1.00	0.23	0.20	0.60	1.84	0.80	4.20	1.27	0.60	2.61	0.42	0.20	0.80
R^2_{rel}							0.96	0.91	0.99	0.96	0.91	1.00	0.93	0.84	0.99	0.98	0.96	0.99	0.98	0.96	1.00	0.86	0.80	0.90
Sens	0.50	0.00	0.83	0.86	0.83	1.00	0.19	0.00	0.33	0.19	0.00	0.50	0.08	0.00	0.17	0.50	0.17	0.83	0.50	0.00	1.00			
AltSens				1.00	1.00	1.00	0.92	0.85	0.98	0.92	0.85	0.98	0.87	0.81	0.93	0.98	0.94	1.00	0.98	0.94	1.00	0.92	0.86	0.98
Sensvar	0.63	0.00	1.00	1.00	1.00	1.00	0.23	0.00	0.40	0.23	0.00	0.60	0.10	0.00	0.20	0.60	0.20	1.00	0.62	0.20	1.00	0.24	0.00	0.70
$Sens_2$	0.86	0.00	1.00	0.13	0.00	1.00				0.00	0.00	0.00	0.00	0.00	0.00				0.20	0.00	1.00			
$AltSens_2$				0.20	0.00	1.00				0.85	0.36	1.00	0.87	0.68	1.00				0.92	0.67	1.00			
FDP				0.95	0.94	0.98	0.47	0.00	1.00	0.55	0.00	1.00	0.70	0.00	1.00	0.62	0.20	0.86	0.70	0.37	1.00			
AltFDP				0.52	0.46	0.59	0.09	0.00	0.47	0.11	0.00	0.54	0.06	0.00	0.46	0.28	0.01	0.62	0.10	0.00	0.38	0.02	0.00	0.08
FDPvar				0.95	0.94	0.95	0.47	0.00	1.00	0.50	0.00	1.00	0.53	0.00	1.00	0.62	0.20	0.86	0.47	0.08	0.80	0.45	0.00	1.00
FDP_2				0.99	0.92	1.00				0.36	0.00	1.00	0.79	0.00	1.00				0.43	0.00	1.00			
$AltFDP_2$				0.05	0.00	0.39				0.04	0.00	0.28	0.10	0.00	0.50				0.09	0.00	0.44			

Table 13: Performance in scenario 2h.

	True model			EWAS$_2$			DSA$_1$			DSA$_2$			Sun3step			LASSO			GLINTERNET			BRT		
	Mean	2.5%	97.5%	Mean	2.5%	97.5%	Mean	2.5%	97.5%	Mean	2.5%	97.5%	Mean	2.5%	97.5%	Mean	2.5%	97.5%	Mean	2.5%	97.5%	Mean	2.5%	97.5%
RMS				16.54	1.07	110.29	0.59	0.14	1.43	0.73	0.29	1.43	0.43	0.14	1.29	2.51	1.14	4.22	2.35	0.93	5.60			
RNV				11.16	1.19	21.00	0.82	0.20	2.00	0.87	0.40	1.60	0.35	0.20	0.60	3.51	1.60	5.90	2.40	1.00	5.21	0.79	0.40	1.40
R^2_{rel}							0.56	0.24	0.75	0.60	0.31	0.86	0.45	0.13	0.78	0.62	0.35	0.76	0.76	0.43	0.94	0.35	0.08	0.64
Sens	0.95	0.71	1.00	0.72	0.14	1.00	0.31	0.00	0.57	0.41	0.14	0.93	0.16	0.00	0.43	0.54	0.29	0.71	0.62	0.25	1.00			
AltSens				0.88	0.36	1.00	0.75	0.39	1.00	0.78	0.46	1.00	0.47	0.21	0.79	0.95	0.69	1.00	0.94	0.69	1.00	0.65	0.37	0.90
Sensvar	0.98	0.80	1.00	0.84	0.20	1.00	0.43	0.00	0.80	0.50	0.20	1.00	0.19	0.00	0.40	0.75	0.40	1.00	0.77	0.40	1.00	0.40	0.00	0.80
Sens$_2$	1.00	1.00	1.00	0.42	0.00	1.00				0.17	0.00	1.00	0.06	0.00	0.50				0.58	0.00	1.00			
AltSens$_2$				0.53	0.00	1.00				0.76	0.30	1.00	0.55	0.05	0.93				0.93	0.66	1.00			
FDP				0.89	0.63	0.99	0.45	0.00	1.00	0.43	0.00	0.80	0.58	0.00	1.00	0.76	0.53	0.89	0.82	0.60	0.96			
AltFDP				0.37	0.16	0.54	0.16	0.00	0.48	0.12	0.00	0.39	0.12	0.00	0.49	0.44	0.19	0.67	0.26	0.03	0.53	0.13	0.00	0.33
FDPvar				0.88	0.62	0.96	0.45	0.00	1.00	0.42	0.00	0.75	0.43	0.00	1.00	0.76	0.53	0.89	0.63	0.24	0.87	0.45	0.00	1.00
FDP$_2$				0.79	0.00	1.00				0.25	0.00	1.00	0.64	0.00	1.00				0.62	0.00	1.00			
AltFDP$_2$				0.12	0.00	0.46				0.06	0.00	0.44	0.17	0.00	0.78				0.19	0.00	0.56			

Table 14: Performance in scenario 3a.

	True model			$EWAS_2$			DSA_1			DSA_2			Sun3step			LASSO			GLINTERNET			BRT		
	Mean	2.5%	97.5%	Mean	2.5%	97.5%	Mean	2.5%	97.5%	Mean	2.5%	97.5%	Mean	2.5%	97.5%	Mean	2.5%	97.5%	Mean	2.5%	97.5%	Mean	2.5%	97.5%
RMS				16.27	0.28	89.72	0.58	0.14	1.07	0.70	0.14	1.43	0.45	0.14	1.01	2.35	1.00	4.07	2.23	0.78	4.58			
RNV				10.57	0.39	21.22	0.82	0.20	1.50	0.84	0.20	1.60	0.37	0.20	0.80	3.30	1.40	5.70	2.28	1.00	4.30	0.70	0.30	1.40
R^2_{rel}							0.56	0.20	0.75	0.58	0.20	0.85	0.44	0.16	0.76	0.62	0.43	0.75	0.75	0.42	0.95	0.31	0.06	0.65
Sens	0.94	0.71	1.00	0.69	0.14	1.00	0.33	0.00	0.71	0.40	0.07	0.71	0.15	0.00	0.57	0.54	0.29	0.71	0.61	0.12	1.00			
AltSens				0.85	0.24	1.00	0.75	0.29	1.00	0.75	0.25	1.00	0.45	0.22	0.76	0.95	0.76	1.00	0.92	0.63	1.00	0.59	0.28	0.88
Sensvar	0.96	0.80	1.00	0.81	0.20	1.00	0.46	0.00	1.00	0.50	0.10	1.00	0.18	0.00	0.60	0.76	0.40	1.00	0.76	0.40	1.00	0.38	0.00	0.80
Sens_2	0.97	0.50	1.00	0.38	0.00	1.00				0.15	0.00	1.00	0.07	0.00	0.50				0.61	0.00	1.00			
AltSens_2				0.46	0.00	1.00				0.74	0.37	1.00	0.55	0.00	0.98				0.91	0.62	1.00			
FDP				0.86	0.24	0.99	0.41	0.00	1.00	0.41	0.00	0.91	0.65	0.00	1.00	0.73	0.38	0.91	0.81	0.61	0.98			
AltFDP				0.37	0.00	0.52	0.14	0.00	0.46	0.12	0.00	0.37	0.14	0.00	0.45	0.44	0.12	0.63	0.27	0.02	0.50	0.13	0.00	0.49
FDPvar				0.84	0.00	0.96	0.41	0.00	1.00	0.39	0.00	0.90	0.53	0.00	1.00	0.73	0.38	0.91	0.61	0.29	0.87	0.42	0.00	1.00
FDP_2				0.76	0.00	1.00				0.25	0.00	1.00	0.69	0.00	1.00				0.58	0.00	1.00			
AltFDP_2				0.12	0.00	0.45				0.04	0.00	0.39	0.20	0.00	1.00				0.20	0.00	0.59			

Table 15: Performance in scenario 3b.

	True model			$EWAS_2$			DSA_1			DSA_2			Sun3step			LASSO			GLINTERNET			BRT		
	Mean	2.5%	97.5%	Mean	2.5%	97.5%	Mean	2.5%	97.5%	Mean	2.5%	97.5%	Mean	2.5%	97.5%	Mean	2.5%	97.5%	Mean	2.5%	97.5%	Mean	2.5%	97.5%
RMS				8.74	0.93	25.71	0.63	0.14	1.29	0.67	0.21	1.29	0.33	0.14	1.00	2.63	1.21	4.44	1.76	0.57	4.07			
RNV				11.01	1.30	21.20	0.88	0.20	1.80	0.91	0.30	1.70	0.32	0.20	0.80	3.68	1.70	6.21	2.05	0.80	4.21	0.78	0.40	1.40
R^2_{rel}							0.74	0.48	0.94	0.71	0.39	0.93	0.46	0.16	0.80	0.79	0.62	0.90	0.76	0.52	0.93	0.37	0.12	0.63
Sens	0.85	0.57	1.00	0.64	0.35	0.86	0.38	0.00	0.71	0.37	0.00	0.71	0.14	0.00	0.43	0.59	0.29	0.71	0.44	0.25	0.75			
AltSens				0.90	0.62	1.00	0.80	0.43	1.00	0.79	0.41	1.00	0.41	0.20	0.73	0.97	0.80	1.00	0.91	0.62	1.00	0.64	0.39	0.90
Sensvar	0.97	0.80	1.00	0.87	0.50	1.00	0.54	0.00	1.00	0.52	0.00	1.00	0.18	0.00	0.40	0.82	0.40	1.00	0.73	0.20	1.00	0.42	0.00	0.80
Sens_2	0.61	0.00	1.00	0.06	0.00	1.00				0.00	0.00	0.00	0.04	0.00	0.50				0.22	0.00	0.50			
AltSens_2				0.07	0.00	1.00				0.44	0.00	0.75	0.52	0.11	0.94				0.74	0.22	1.00			
FDP				0.87	0.45	0.97	0.38	0.00	1.00	0.42	0.00	1.00	0.56	0.00	1.00	0.75	0.52	0.89	0.82	0.60	0.96			
AltFDP				0.38	0.06	0.50	0.13	0.00	0.44	0.16	0.00	0.48	0.11	0.00	0.51	0.46	0.21	0.64	0.25	0.02	0.50	0.12	0.00	0.42
FDPvar				0.87	0.45	0.96	0.38	0.00	1.00	0.41	0.00	1.00	0.42	0.00	1.00	0.75	0.52	0.89	0.58	0.18	0.85	0.43	0.00	1.00
FDP_2				0.88	0.43	0.99				0.10	0.00	1.00	0.42	0.00	1.00				0.47	0.00	1.00			
AltFDP_2				0.02	0.00	0.31				0.04	0.00	0.61	0.10	0.00	0.71				0.18	0.00	0.70			

Table 16: Performance in scenario 3c.

	True model			$EWAS_2$			DSA_1			DSA_2			Sun3step			LASSO			GLINTERNET			BRT		
	Mean	2.5%	97.5%	Mean	2.5%	97.5%	Mean	2.5%	97.5%	Mean	2.5%	97.5%	Mean	2.5%	97.5%	Mean	2.5%	97.5%	Mean	2.5%	97.5%	Mean	2.5%	97.5%
RMS				10.11	1.78	30.69	0.66	0.29	1.29	0.68	0.29	1.29	0.36	0.14	1.13	2.48	1.43	4.22	1.86	0.50	4.59			
RNV				12.40	2.49	21.72	0.93	0.40	1.80	0.91	0.40	1.70	0.34	0.20	0.78	3.47	2.00	5.90	2.12	0.70	4.63	0.78	0.40	1.40
R^2_{rel}							0.73	0.45	0.91	0.73	0.46	0.90	0.49	0.18	0.77	0.79	0.62	0.90	0.76	0.49	0.96	0.37	0.11	0.59
Sens	0.86	0.57	1.00	0.65	0.35	0.86	0.36	0.07	0.71	0.37	0.07	0.57	0.14	0.00	0.43	0.55	0.29	0.71	0.39	0.00	0.75			
AltSens				0.92	0.61	1.00	0.81	0.49	1.00	0.81	0.50	0.99	0.44	0.23	0.74	0.96	0.83	1.00	0.90	0.63	1.00	0.63	0.34	0.86
Sensvar	0.98	0.80	1.00	0.89	0.50	1.00	0.50	0.10	1.00	0.51	0.10	0.80	0.20	0.00	0.60	0.77	0.40	1.00	0.68	0.40	1.00	0.38	0.00	0.80
$Sens_2$	0.59	0.00	1.00	0.04	0.00	0.50				0.00	0.00	0.00	0.02	0.00	0.46				0.14	0.00	0.50			
$AltSens_2$				0.09	0.00	0.86				0.57	0.10	0.87	0.50	0.00	0.85				0.71	0.18	1.00			
FDP				0.90	0.71	0.97	0.43	0.00	0.94	0.42	0.00	0.95	0.55	0.00	1.00	0.76	0.57	0.92	0.85	0.67	1.00			
AltFDP				0.40	0.17	0.52	0.15	0.00	0.41	0.14	0.00	0.44	0.12	0.00	0.43	0.43	0.19	0.63	0.25	0.07	0.47	0.15	0.00	0.43
FDPvar				0.89	0.71	0.96	0.43	0.00	0.94	0.40	0.00	0.95	0.42	0.00	1.00	0.76	0.57	0.92	0.62	0.31	0.87	0.47	0.00	1.00
FDP_2				0.88	0.20	1.00				0.15	0.00	1.00	0.51	0.00	1.00				0.63	0.00	1.00			
$AltFDP_2$				0.04	0.00	0.39				0.03	0.00	0.46	0.14	0.00	0.84				0.23	0.00	0.76			

Table 17: Performance in scenario 3d.

	True model			EWAS$_2$			DSA$_1$			DSA$_2$			Sun3step			LASSO			GLINTERNET			BRT		
	Mean	2.5%	97.5%	Mean	2.5%	97.5%	Mean	2.5%	97.5%	Mean	2.5%	97.5%	Mean	2.5%	97.5%	Mean	2.5%	97.5%	Mean	2.5%	97.5%	Mean	2.5%	97.5%
RMS				179.33	76.50	331.60	0.30	0.14	0.57	0.60	0.29	1.29	0.34	0.29	0.71	1.42	0.43	3.29	1.77	0.86	3.72			
RNV				17.90	14.69	20.70	0.42	0.20	0.80	0.61	0.20	1.40	0.24	0.20	0.50	1.99	0.60	4.60	1.59	0.80	3.41	0.48	0.20	1.40
R^2_{rel}							0.75	0.68	0.79	0.95	0.87	0.99	0.90	0.82	0.99	0.77	0.72	0.81	0.98	0.95	1.00	0.83	0.75	0.89
Sens	0.45	0.14	0.79	1.00	1.00	1.00	0.15	0.00	0.29	0.26	0.00	0.57	0.08	0.00	0.14	0.43	0.14	0.71	0.54	0.25	1.00			
AltSens				1.00	1.00	1.00	0.91	0.84	0.97	0.93	0.87	0.99	0.88	0.82	0.93	0.98	0.94	1.00	0.99	0.96	1.00	0.92	0.86	1.00
Sensvar	0.65	0.30	1.00	1.00	1.00	1.00	0.21	0.00	0.40	0.32	0.00	0.60	0.11	0.00	0.20	0.60	0.20	1.00	0.72	0.40	1.00	0.29	0.00	0.80
Sens$_2$	0.76	0.00	1.00	1.00	1.00	1.00				0.10	0.00	0.50	0.00	0.00	0.00				0.42	0.00	1.00			
AltSens$_2$				1.00	1.00	1.00				0.93	0.83	1.00	0.87	0.79	0.95				0.99	0.95	1.00			
FDP				0.99	0.99	1.00	0.46	0.00	1.00	0.55	0.00	1.00	0.74	0.50	1.00	0.64	0.25	0.89	0.81	0.58	0.96			
AltFDP				0.50	0.43	0.57	0.11	0.00	0.51	0.08	0.00	0.45	0.07	0.00	0.47	0.32	0.02	0.62	0.15	0.00	0.45	0.02	0.00	0.07
FDPvar				0.94	0.93	0.95	0.46	0.00	1.00	0.42	0.00	1.00	0.48	0.00	1.00	0.64	0.25	0.89	0.50	0.08	0.80	0.39	0.00	1.00
FDP$_2$				1.00	1.00	1.00				0.80	0.00	1.00	1.00	1.00	1.00				0.78	0.33	1.00			
AltFDP$_2$				0.48	0.39	0.54				0.04	0.00	0.46	0.08	0.00	0.49				0.09	0.00	0.37			

Table 18: Performance in scenario 3e.

	True model			EWAS$_2$			DSA$_1$			DSA$_2$			Sun3step			LASSO			GLINTERNET			BRT		
	Mean	2.5%	97.5%	Mean	2.5%	97.5%	Mean	2.5%	97.5%	Mean	2.5%	97.5%	Mean	2.5%	97.5%	Mean	2.5%	97.5%	Mean	2.5%	97.5%	Mean	2.5%	97.5%
RMS				184.06	87.25	318.00	0.30	0.14	0.86	0.58	0.29	1.22	0.31	0.29	0.57	1.32	0.50	3.08	1.64	0.86	3.01			
RNV				17.73	15.20	20.30	0.42	0.20	1.20	0.58	0.20	1.30	0.22	0.20	0.40	1.85	0.70	4.31	1.47	0.80	2.50	0.41	0.20	0.80
R^2_{rel}							0.75	0.68	0.80	0.95	0.88	1.00	0.92	0.80	0.99	0.77	0.73	0.81	0.98	0.95	1.00	0.84	0.76	0.90
Sens	0.41	0.14	0.86	1.00	1.00	1.00	0.13	0.00	0.29	0.26	0.00	0.57	0.08	0.00	0.14	0.37	0.14	0.71	0.44	0.00	0.75			
AltSens				1.00	1.00	1.00	0.91	0.84	0.96	0.93	0.87	0.99	0.88	0.83	0.93	0.97	0.94	1.00	0.99	0.95	1.00	0.91	0.86	0.98
Sensvar	0.62	0.20	1.00	1.00	1.00	1.00	0.18	0.00	0.40	0.33	0.00	0.60	0.11	0.00	0.20	0.52	0.20	1.00	0.67	0.20	1.00	0.23	0.00	0.60
Sens$_2$	0.69	0.00	1.00	1.00	1.00	1.00				0.08	0.00	0.50	0.00	0.00	0.00				0.29	0.00	1.00			
AltSens$_2$				1.00	1.00	1.00				0.92	0.85	0.99	0.88	0.81	0.94				0.99	0.93	1.00			
FDP				0.99	0.99	1.00	0.49	0.00	1.00	0.54	0.00	1.00	0.74	0.50	1.00	0.65	0.21	0.92	0.83	0.60	1.00			
AltFDP				0.50	0.43	0.56	0.10	0.00	0.52	0.09	0.00	0.48	0.05	0.00	0.47	0.31	0.00	0.70	0.12	0.00	0.41	0.02	0.00	0.07
FDPvar				0.94	0.93	0.95	0.49	0.00	1.00	0.40	0.00	1.00	0.49	0.00	1.00	0.65	0.21	0.92	0.51	0.00	0.79	0.45	0.00	1.00
FDP$_2$				1.00	1.00	1.00				0.84	0.00	1.00	1.00	1.00	1.00				0.84	0.24	1.00			
AltFDP$_2$				0.48	0.41	0.54				0.05	0.00	0.45	0.06	0.00	0.39				0.07	0.00	0.28			

Table 19: Performance in scenario 3f.

	True model			$EWAS_2$			DSA_1			DSA_2			Sun3step			LASSO			GLINTERNET			BRT		
	Mean	2.5%	97.5%	Mean	2.5%	97.5%	Mean	2.5%	97.5%	Mean	2.5%	97.5%	Mean	2.5%	97.5%	Mean	2.5%	97.5%	Mean	2.5%	97.5%	Mean	2.5%	97.5%
RMS				64.89	14.48	178.58	0.35	0.14	0.93	0.57	0.29	1.07	0.35	0.29	1.00	1.42	0.57	3.07	1.41	0.64	2.93			
RNV				18.87	16.09	21.80	0.48	0.20	1.30	0.59	0.20	1.20	0.25	0.20	0.60	1.99	0.80	4.30	1.42	0.70	2.60	0.40	0.20	0.80
R^2_{rel}							0.91	0.87	0.95	0.96	0.90	1.00	0.93	0.85	0.99	0.93	0.90	0.95	0.98	0.95	1.01	0.86	0.80	0.91
Sens	0.39	0.07	0.86	0.99	0.86	1.00	0.16	0.00	0.43	0.23	0.00	0.57	0.07	0.00	0.14	0.43	0.14	0.71	0.39	0.00	0.75			
AltSens				1.00	1.00	1.00	0.92	0.85	0.98	0.93	0.87	0.99	0.88	0.83	0.93	0.99	0.95	1.00	0.99	0.96	1.00	0.92	0.87	0.98
Sensvar	0.55	0.10	1.00	1.00	1.00	1.00	0.23	0.00	0.60	0.30	0.00	0.60	0.10	0.00	0.20	0.60	0.20	1.00	0.63	0.30	1.00	0.24	0.00	0.60
$Sens_2$	0.36	0.00	1.00	0.95	0.50	1.00				0.04	0.00	0.50	0.00	0.00	0.00				0.17	0.00	0.76			
$AltSens_2$				0.99	0.93	1.00				0.90	0.79	1.00	0.88	0.80	0.96				0.97	0.91	1.00			
FDP				0.98	0.94	0.99	0.48	0.00	1.00	0.60	0.22	1.00	0.76	0.50	1.00	0.64	0.24	0.91	0.82	0.55	1.00			
AltFDP				0.52	0.46	0.57	0.11	0.00	0.51	0.09	0.00	0.43	0.07	0.00	0.54	0.30	0.01	0.65	0.10	0.00	0.41	0.02	0.00	0.08
FDPvar				0.95	0.94	0.95	0.48	0.00	1.00	0.46	0.00	1.00	0.52	0.00	1.00	0.64	0.24	0.91	0.52	0.20	0.77	0.38	0.00	1.00
FDP_2				0.98	0.85	1.00				0.92	0.00	1.00	1.00	1.00	1.00				0.83	0.00	1.00			
$AltFDP_2$				0.35	0.02	0.52				0.05	0.00	0.40	0.09	0.00	0.56				0.08	0.00	0.42			

Table 20: Performance in scenario 3g.

	True model			$EWAS_2$			DSA_1			DSA_2			Sun3step			LASSO			GLINTERNET			BRT		
	Mean	2.5%	97.5%	Mean	2.5%	97.5%	Mean	2.5%	97.5%	Mean	2.5%	97.5%	Mean	2.5%	97.5%	Mean	2.5%	97.5%	Mean	2.5%	97.5%	Mean	2.5%	97.5%
RMS				64.50	12.86	184.57	0.34	0.14	0.65	0.57	0.29	1.14	0.32	0.29	0.57	1.30	0.57	2.50	1.45	0.71	2.65			
RNV				19.13	16.40	21.91	0.47	0.20	0.90	0.59	0.20	1.20	0.22	0.20	0.40	1.82	0.80	3.50	1.42	0.60	2.70	0.48	0.20	0.80
R^2_{rel}							0.91	0.86	0.95	0.96	0.91	1.00	0.93	0.85	1.00	0.93	0.91	0.95	0.98	0.95	1.01	0.86	0.80	0.91
Sens	0.40	0.14	0.71	0.97	0.71	1.00	0.18	0.00	0.36	0.23	0.00	0.57	0.07	0.00	0.14	0.40	0.14	0.65	0.40	0.00	0.75			
AltSens				1.00	1.00	1.00	0.92	0.86	0.98	0.93	0.87	0.99	0.88	0.83	0.93	0.98	0.94	1.00	0.98	0.95	1.00	0.93	0.87	0.98
Sensvar	0.57	0.20	1.00	1.00	1.00	1.00	0.25	0.00	0.50	0.31	0.00	0.60	0.09	0.00	0.20	0.57	0.20	0.90	0.65	0.40	1.00	0.27	0.00	0.80
$Sens_2$	0.32	0.00	1.00	0.90	0.00	1.00				0.02	0.00	0.50	0.00	0.00	0.00				0.24	0.00	1.00			
$AltSens_2$				0.91	0.00	1.00				0.89	0.79	0.99	0.88	0.79	0.97				0.97	0.89	1.00			
FDP				0.97	0.94	0.99	0.42	0.00	1.00	0.59	0.20	1.00	0.78	0.50	1.00	0.64	0.22	0.91	0.83	0.60	1.00			
AltFDP				0.52	0.45	0.58	0.09	0.00	0.48	0.10	0.00	0.48	0.06	0.00	0.46	0.30	0.00	0.66	0.11	0.00	0.37	0.02	0.00	0.09
FDPvar				0.95	0.94	0.95	0.42	0.00	1.00	0.45	0.00	1.00	0.56	0.00	1.00	0.64	0.22	0.91	0.49	0.00	0.81	0.42	0.00	1.00
FDP_2				0.98	0.93	1.00				0.94	0.00	1.00	0.99	1.00	1.00				0.81	0.00	1.00			
$AltFDP_2$				0.32	0.00	0.51				0.05	0.00	0.34	0.08	0.00	0.46				0.07	0.00	0.33			

Table 21: Performance in scenario 3h.

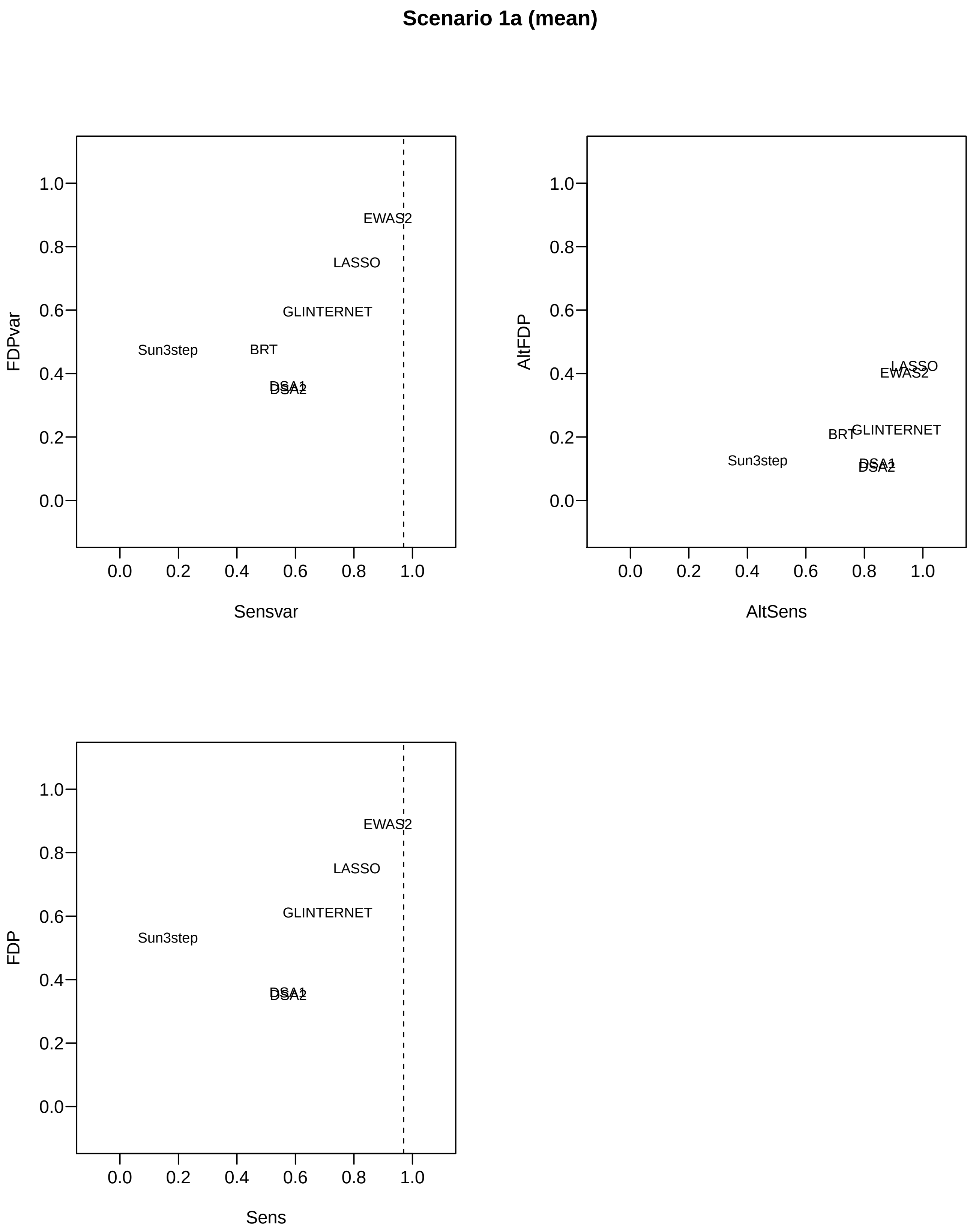

Figure 6: Measures of sensitivity vs FDP in scenario 1a.

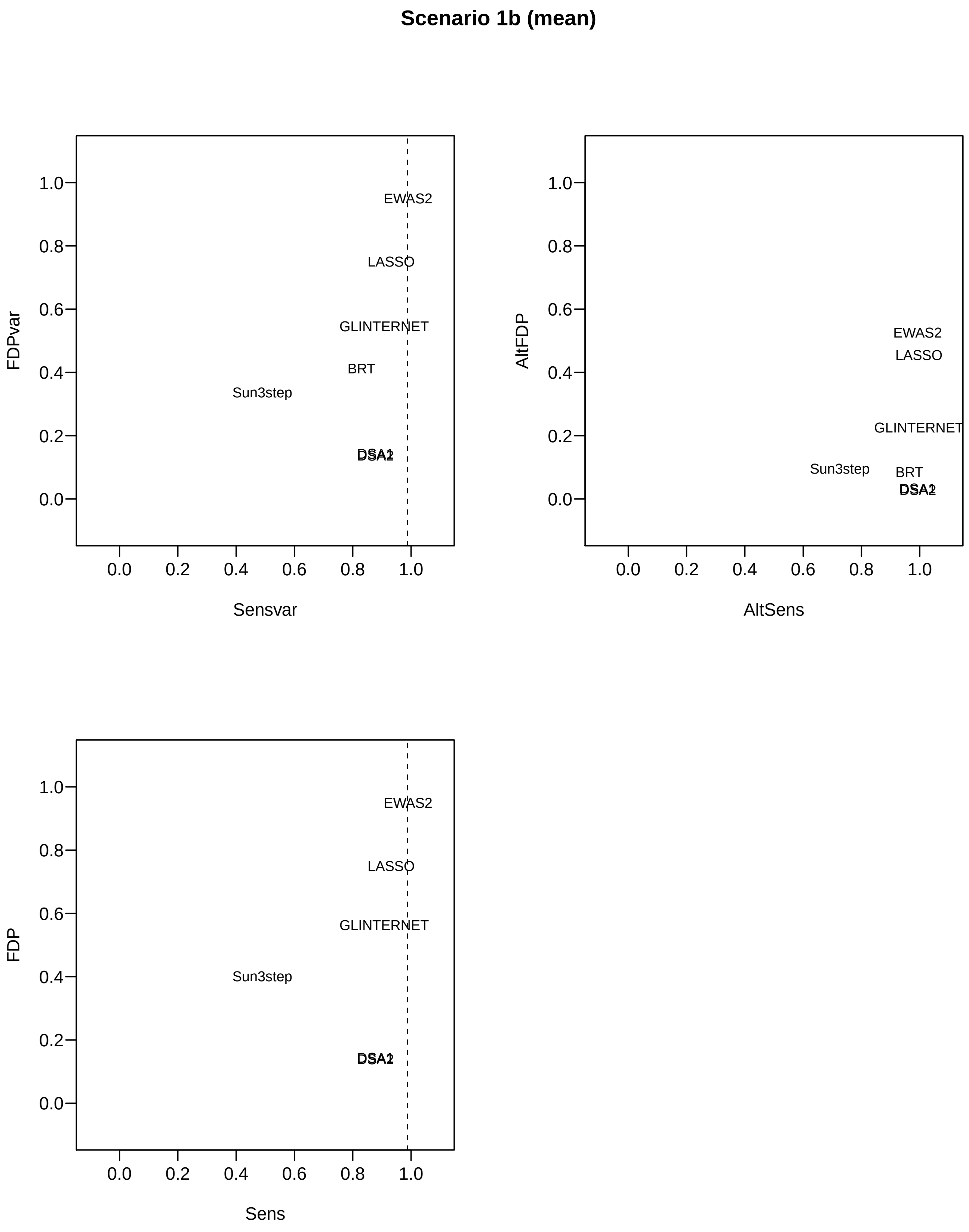

Figure 7: Measures of sensitivity vs FDP in scenario 1b.

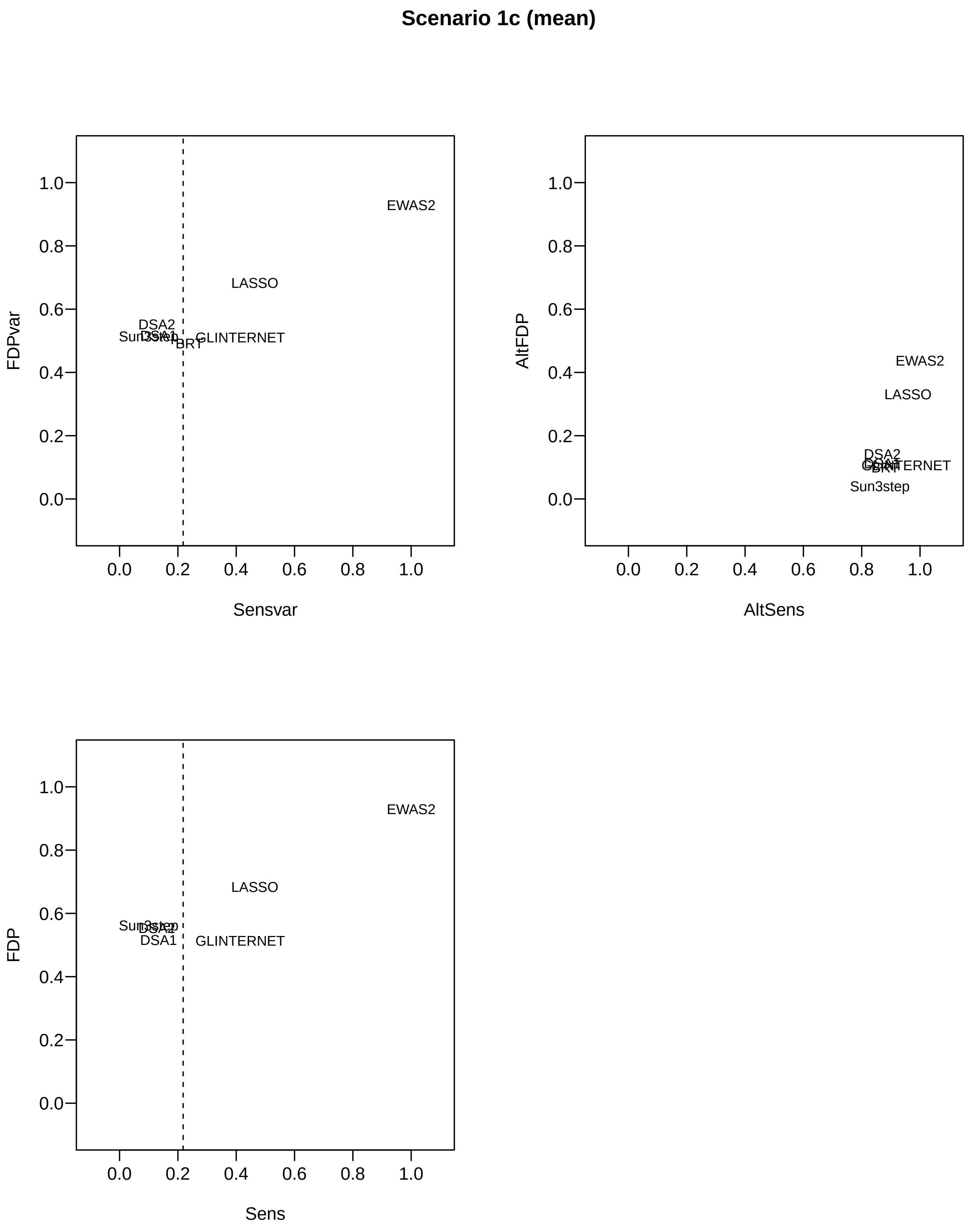

Figure 8: Measures of sensitivity vs FDP in scenario 1c.

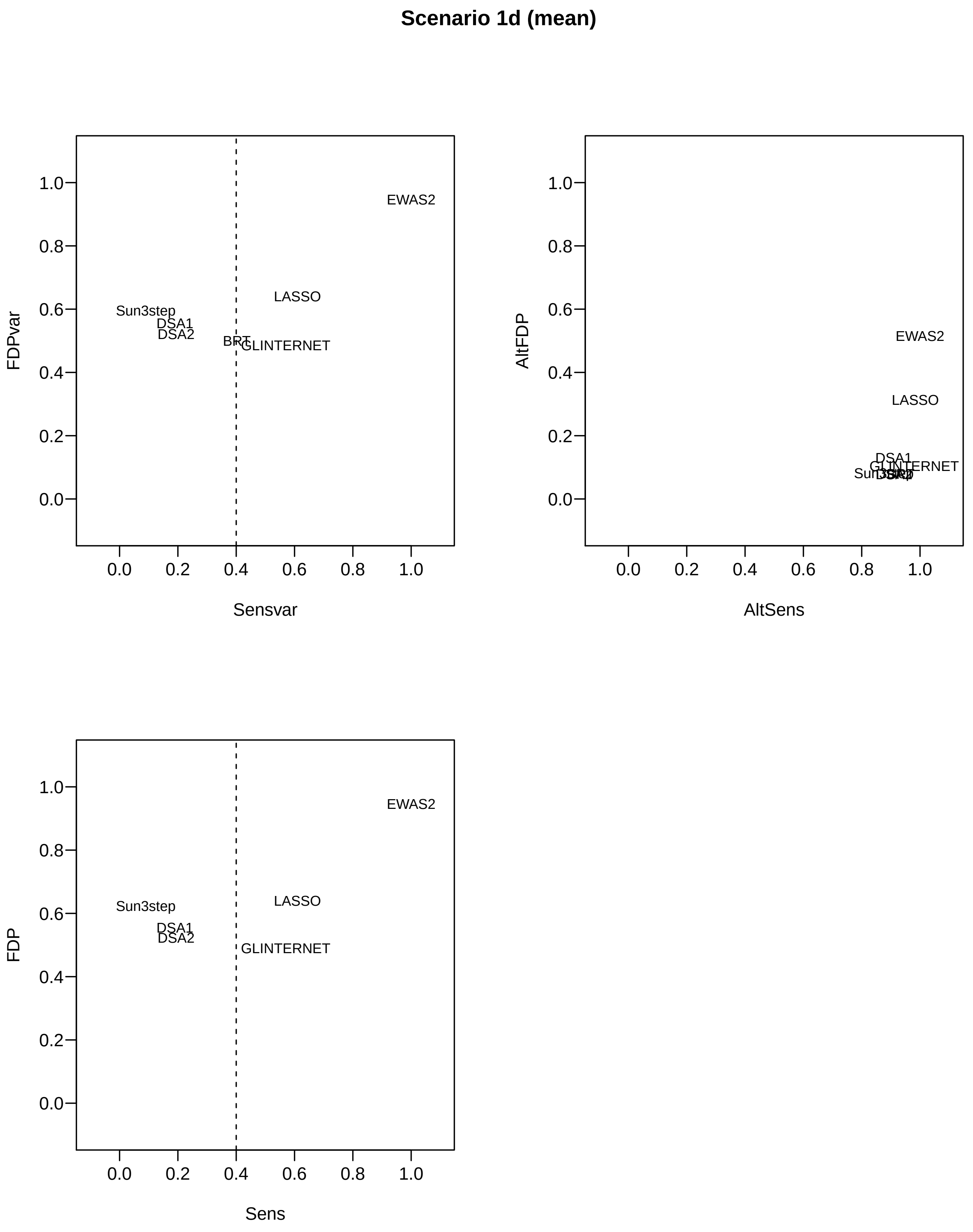

Figure 9: Measures of sensitivity vs FDP in scenario 1d.

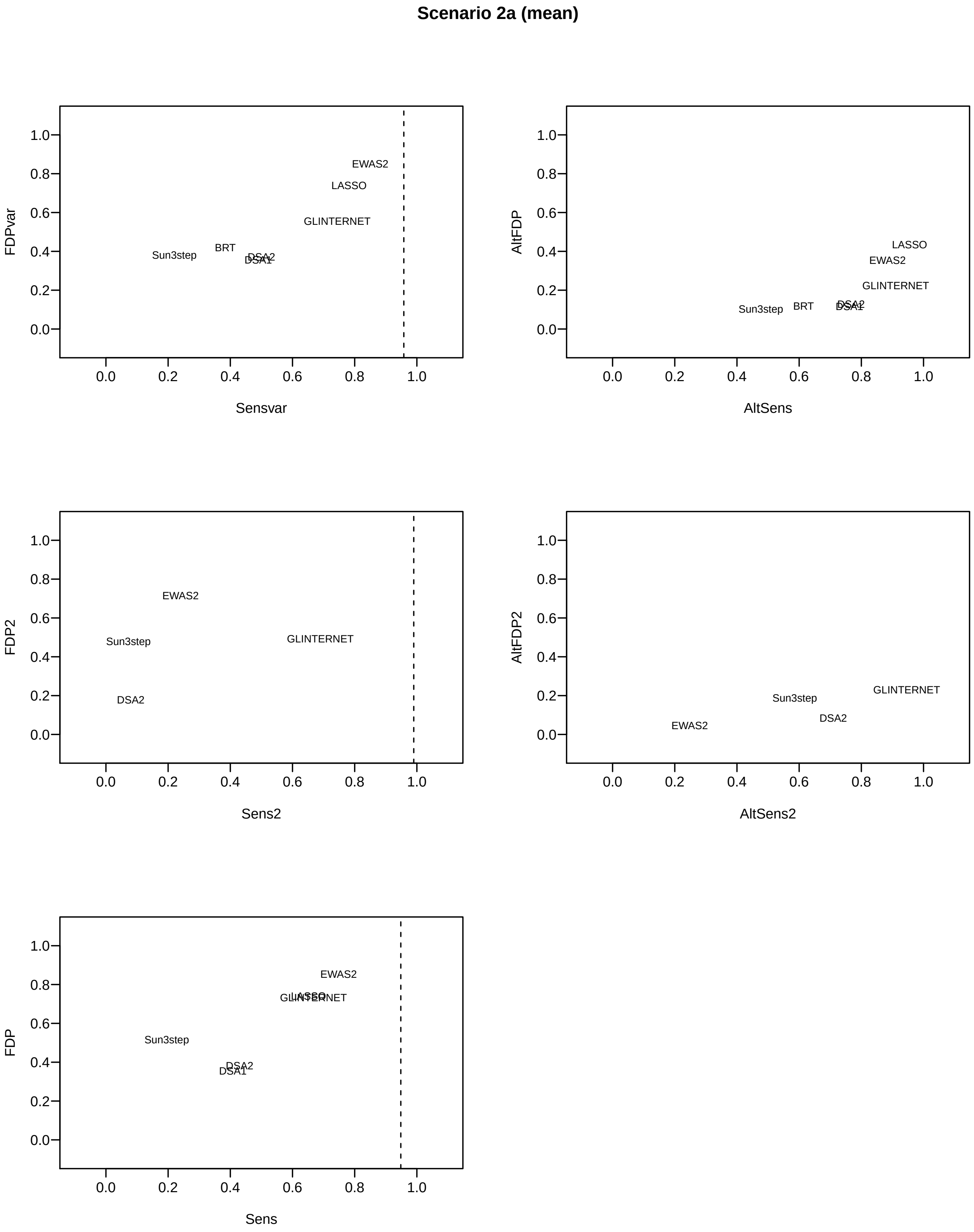

Figure 10: Measures of sensitivity vs FDP in scenario 2a.

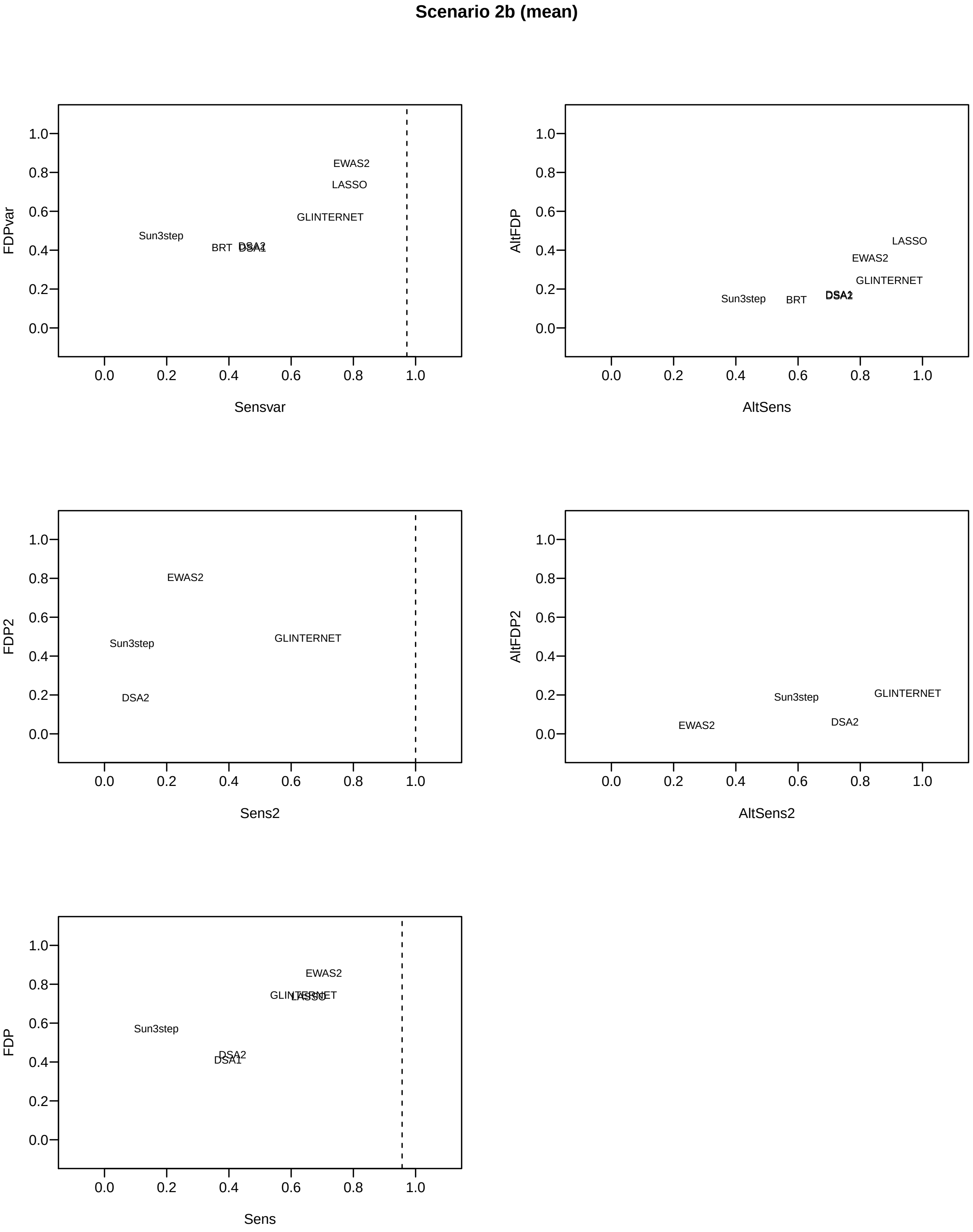

Figure 11: Measures of sensitivity vs FDP in scenario 2b.

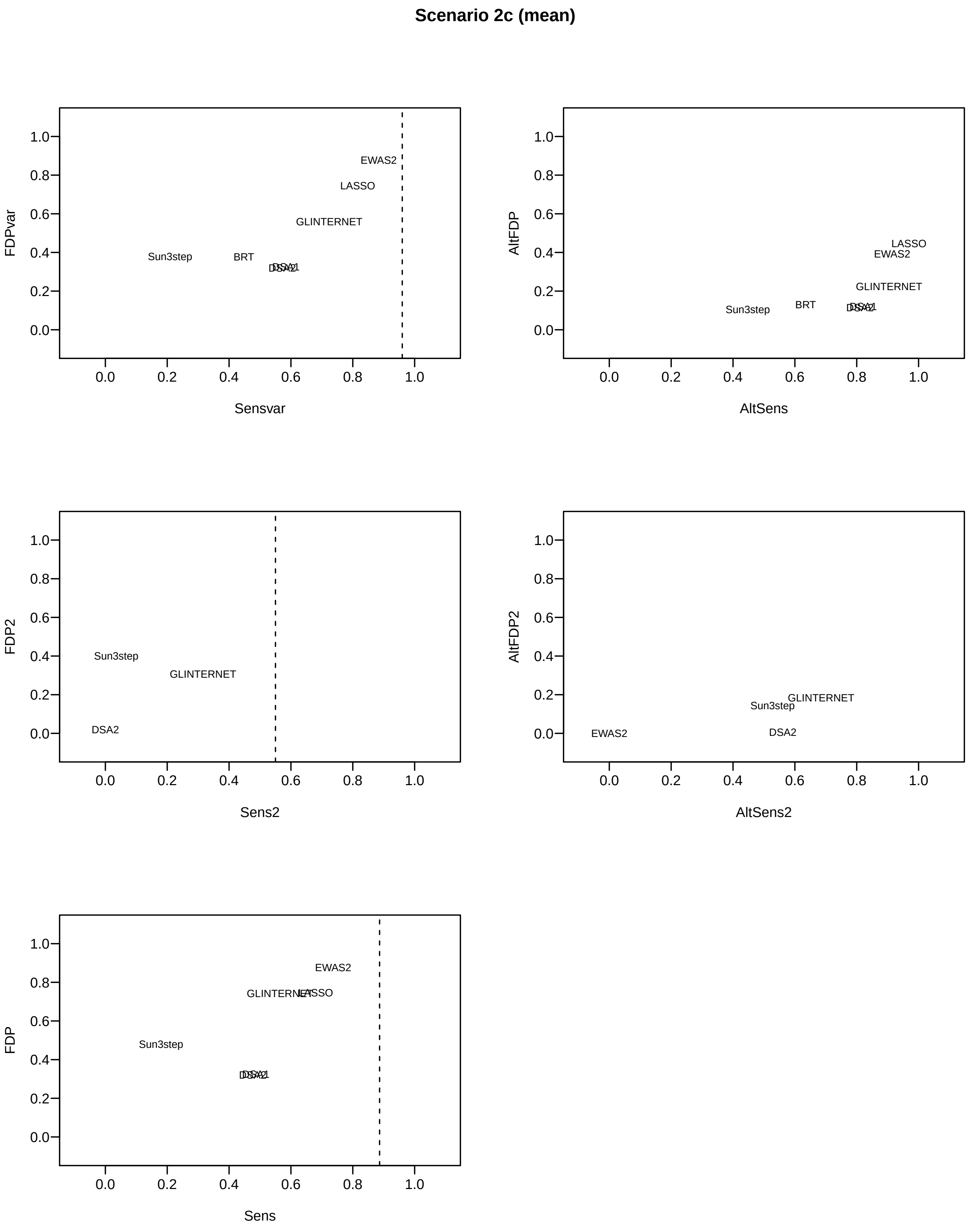

Figure 12: Measures of sensitivity vs FDP in scenario 2c.

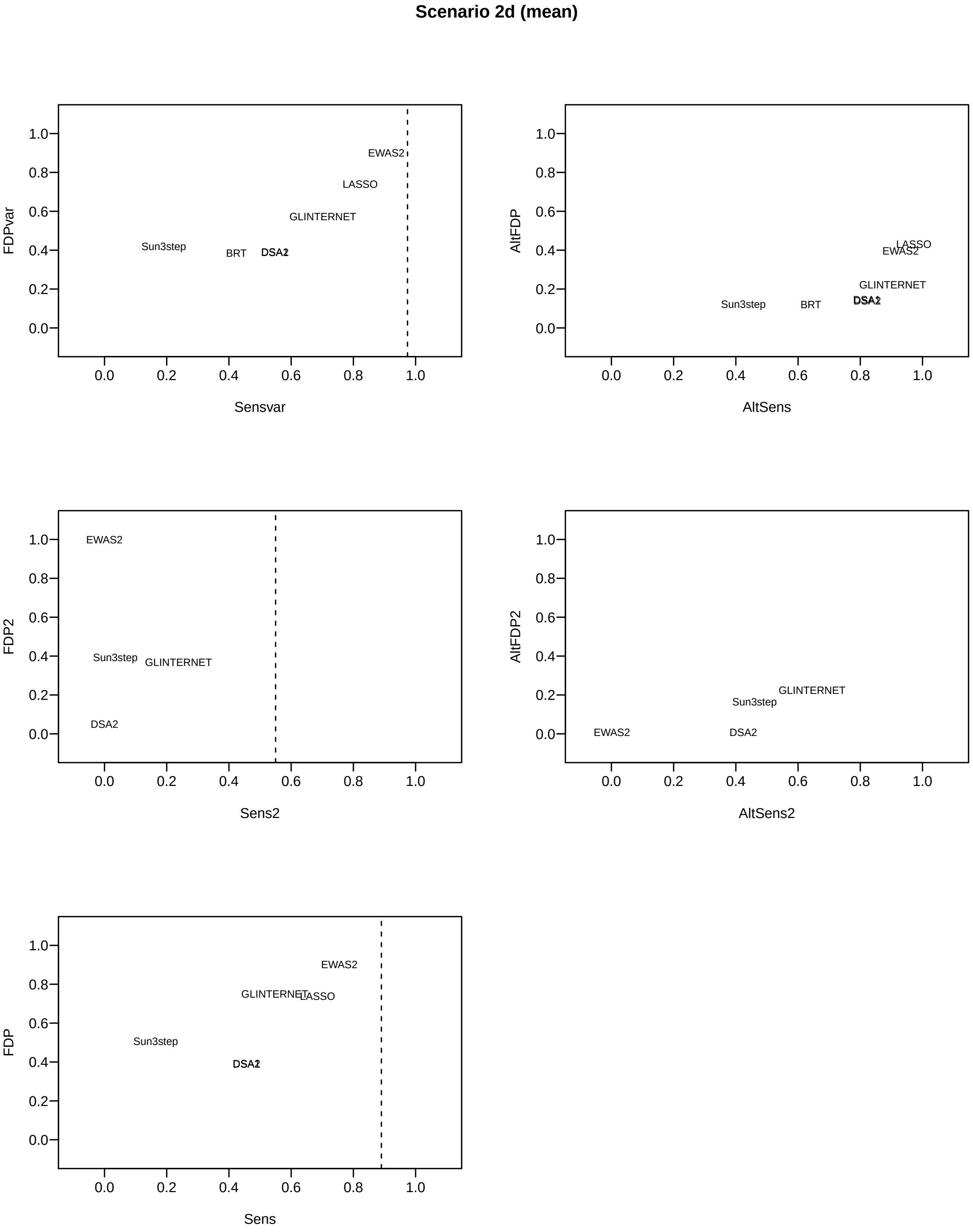

Figure 13: Measures of sensitivity vs FDP in scenario 2d.

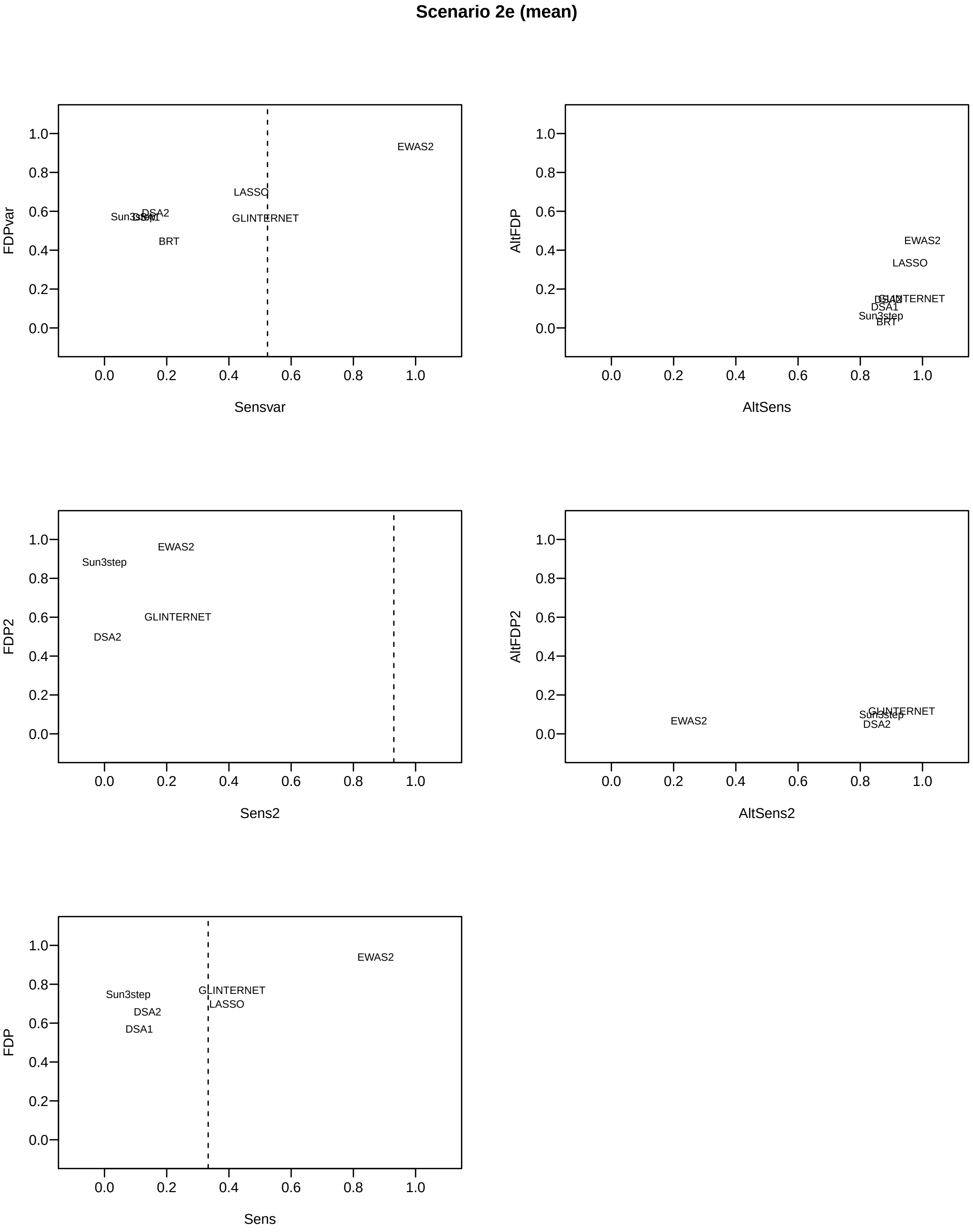

Figure 14: Measures of sensitivity vs FDP in scenario 2e.

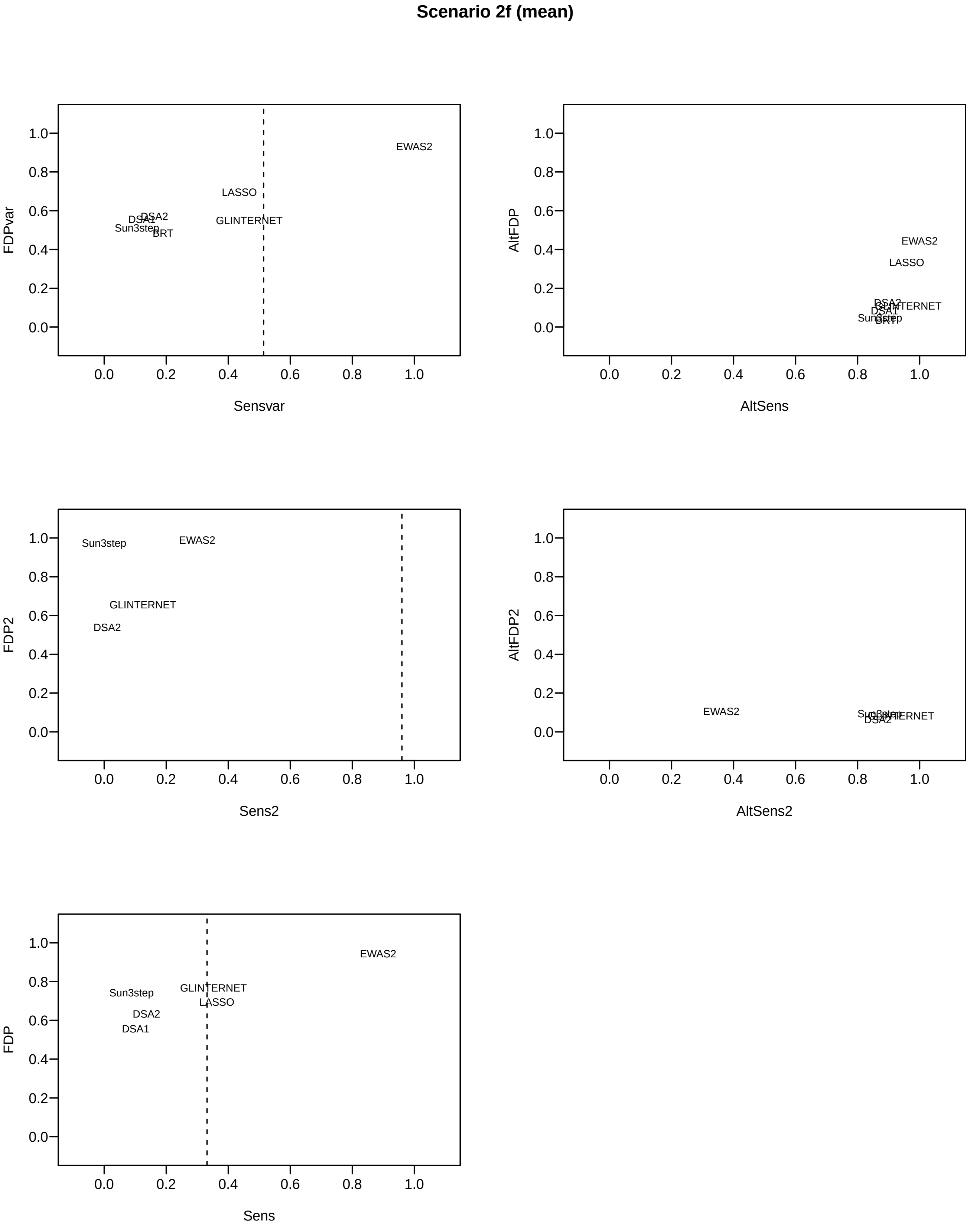

Figure 15: Measures of sensitivity vs FDP in scenario 2f.

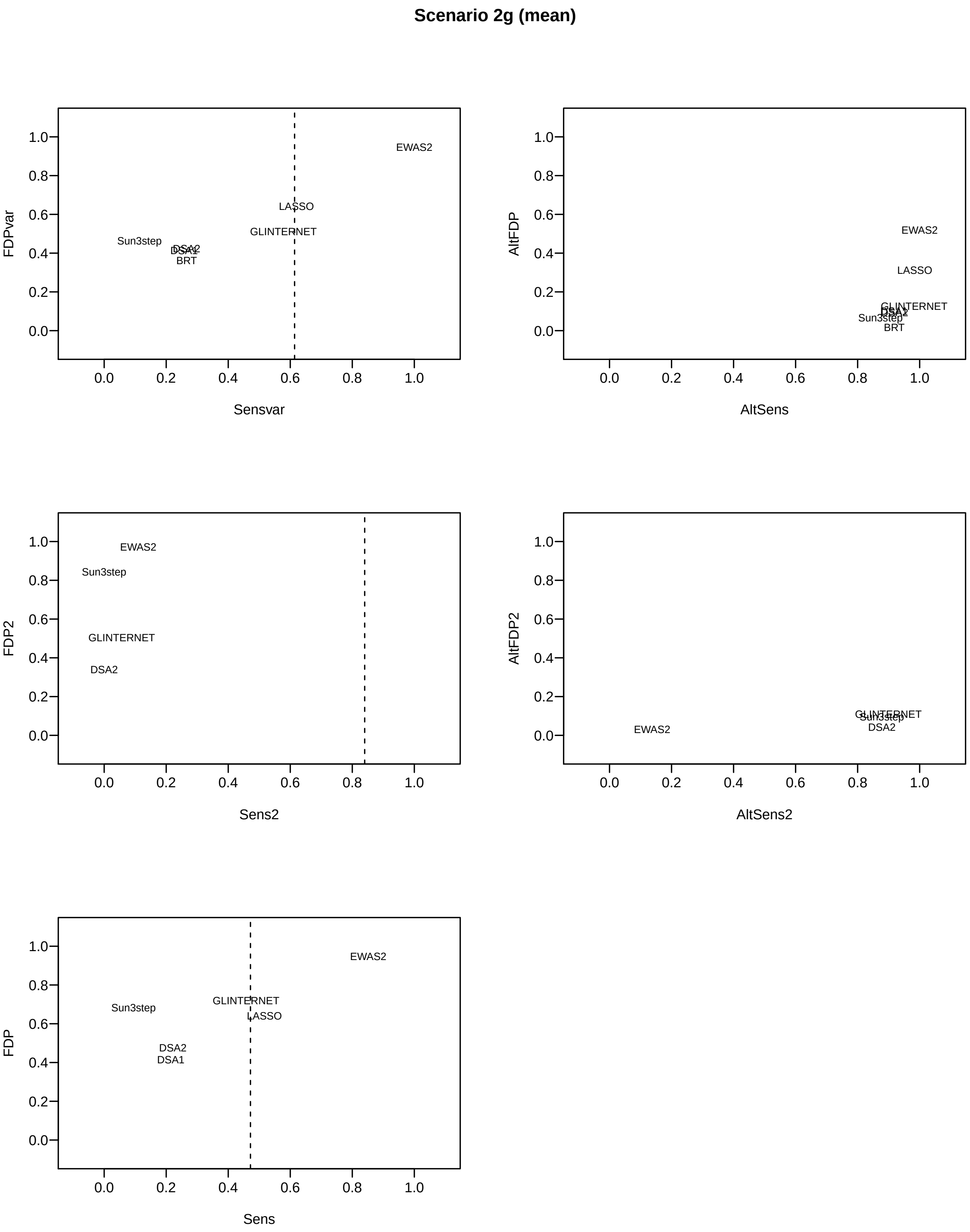

Figure 16: Measures of sensitivity vs FDP in scenario 2g.

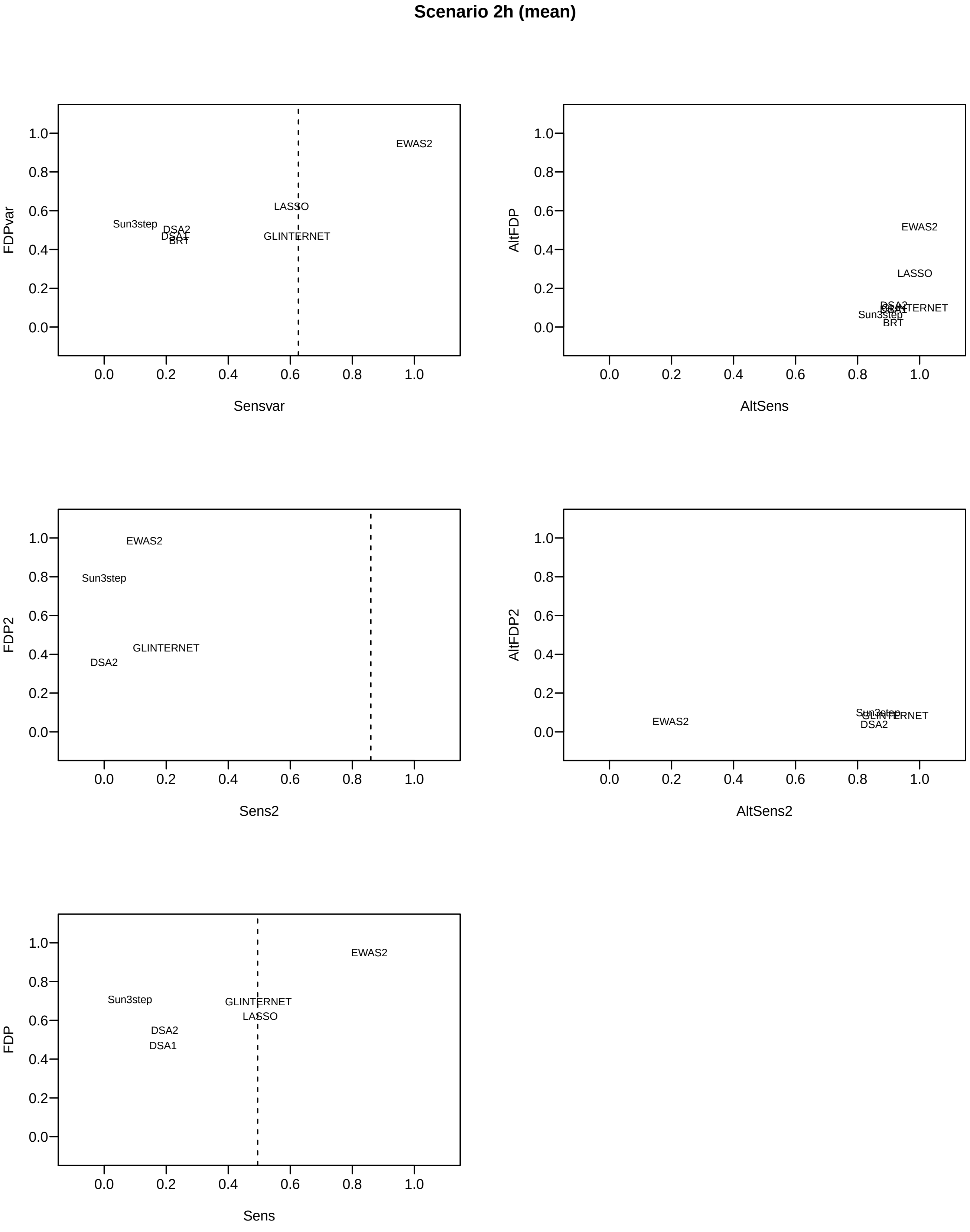

Figure 17: Measures of sensitivity vs FDP in scenario 2h.

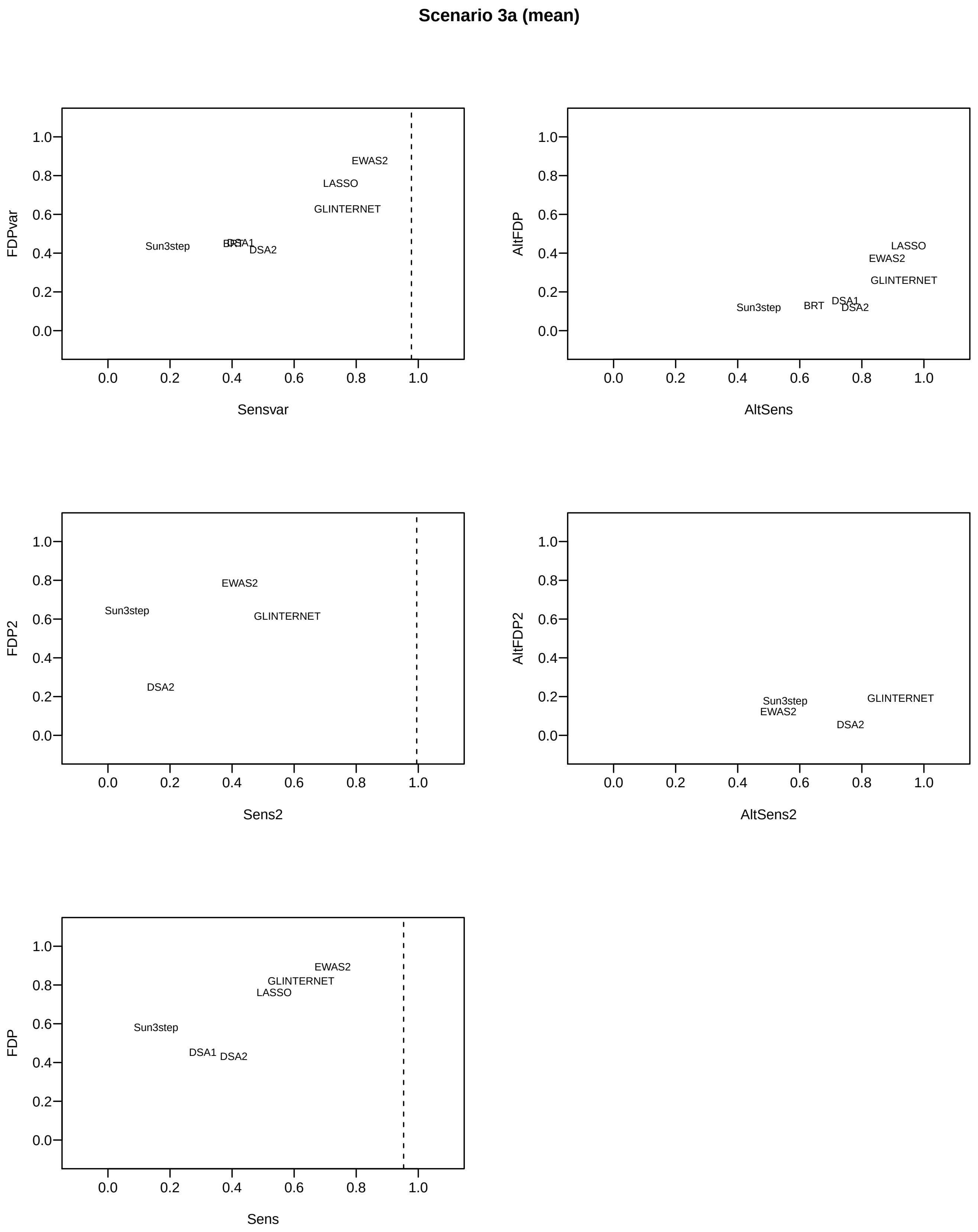

Figure 18: Measures of sensitivity vs FDP in scenario 3a.

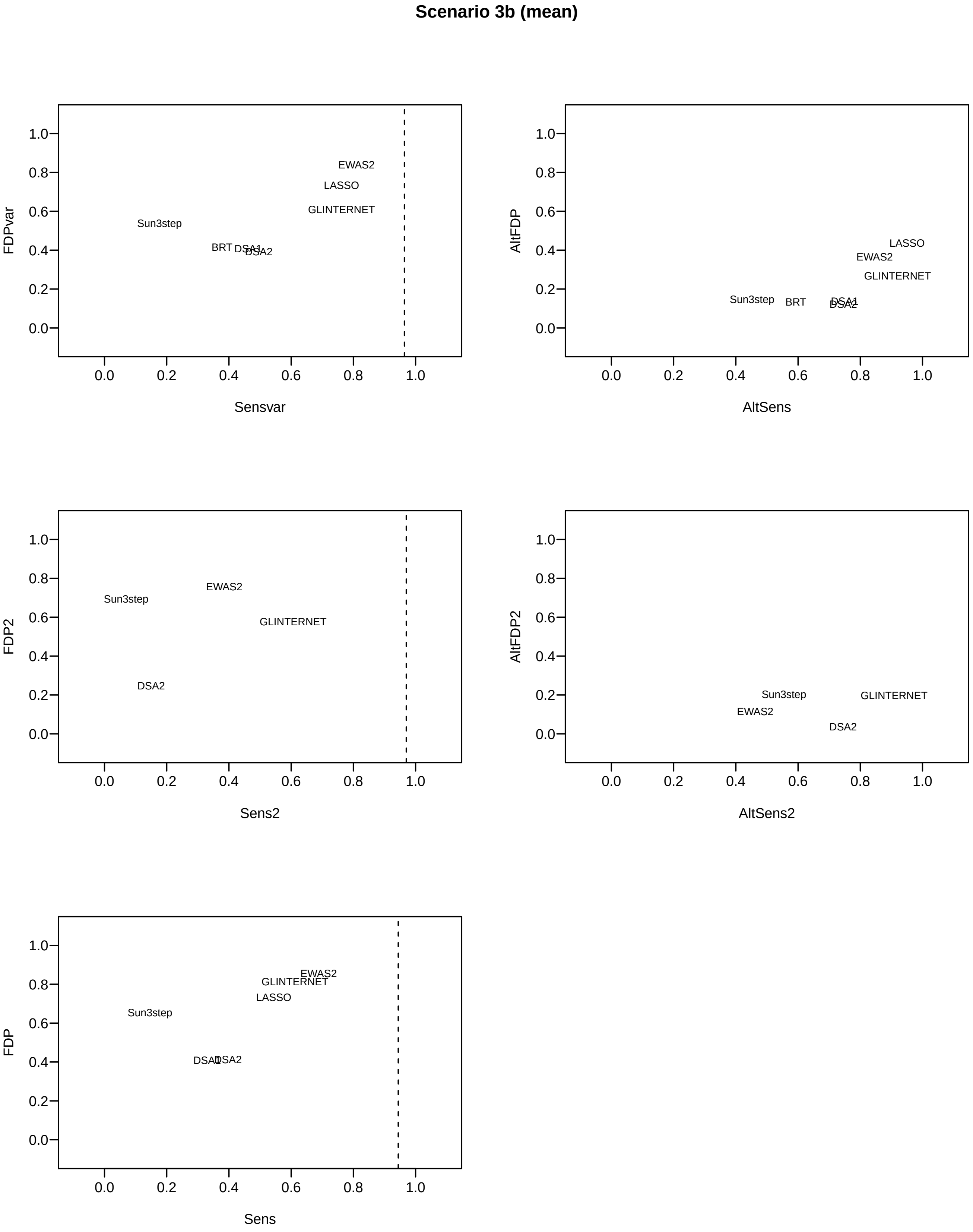

Figure 19: Measures of sensitivity vs FDP in scenario 3b.

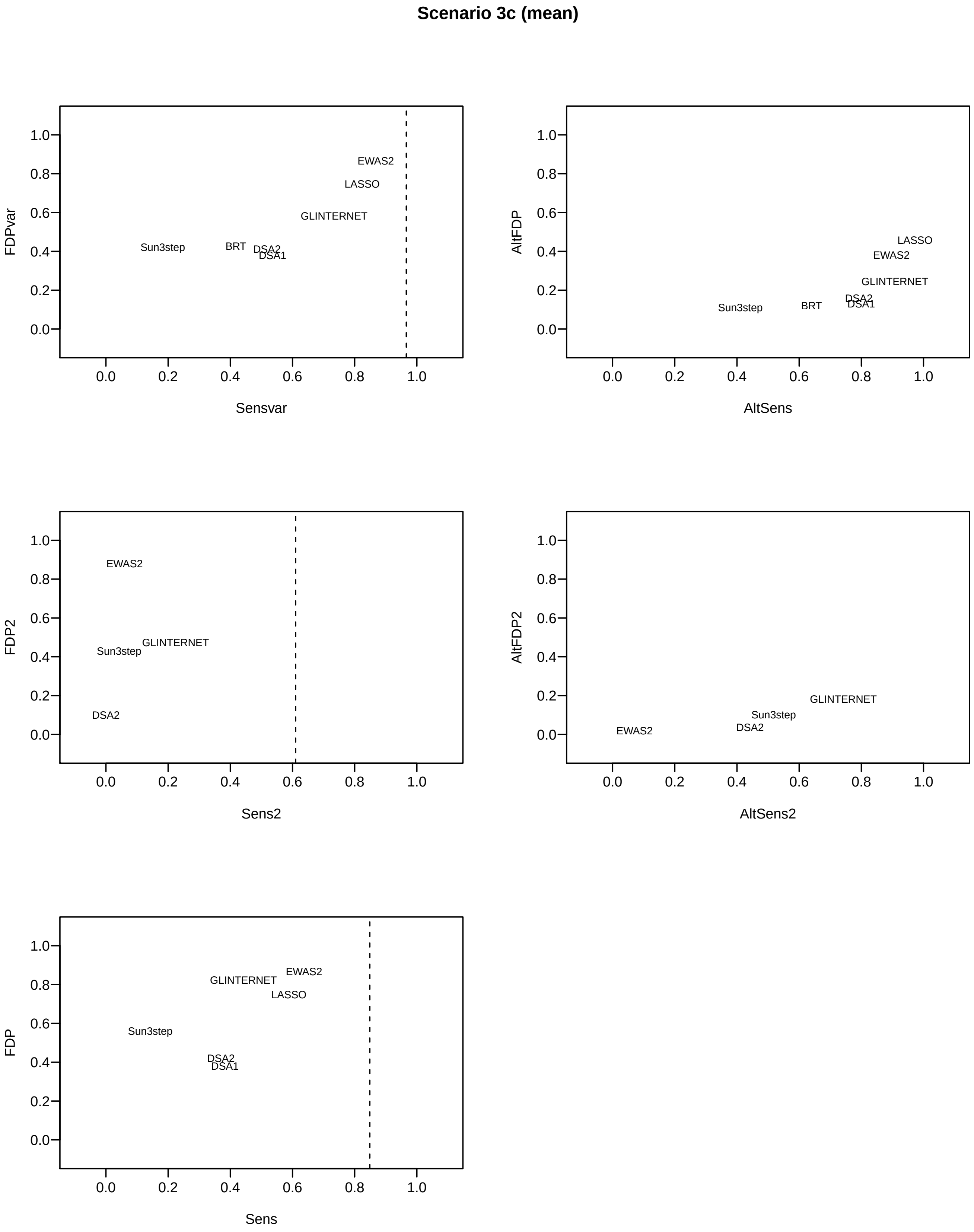

Figure 20: Measures of sensitivity vs FDP in scenario 3c.

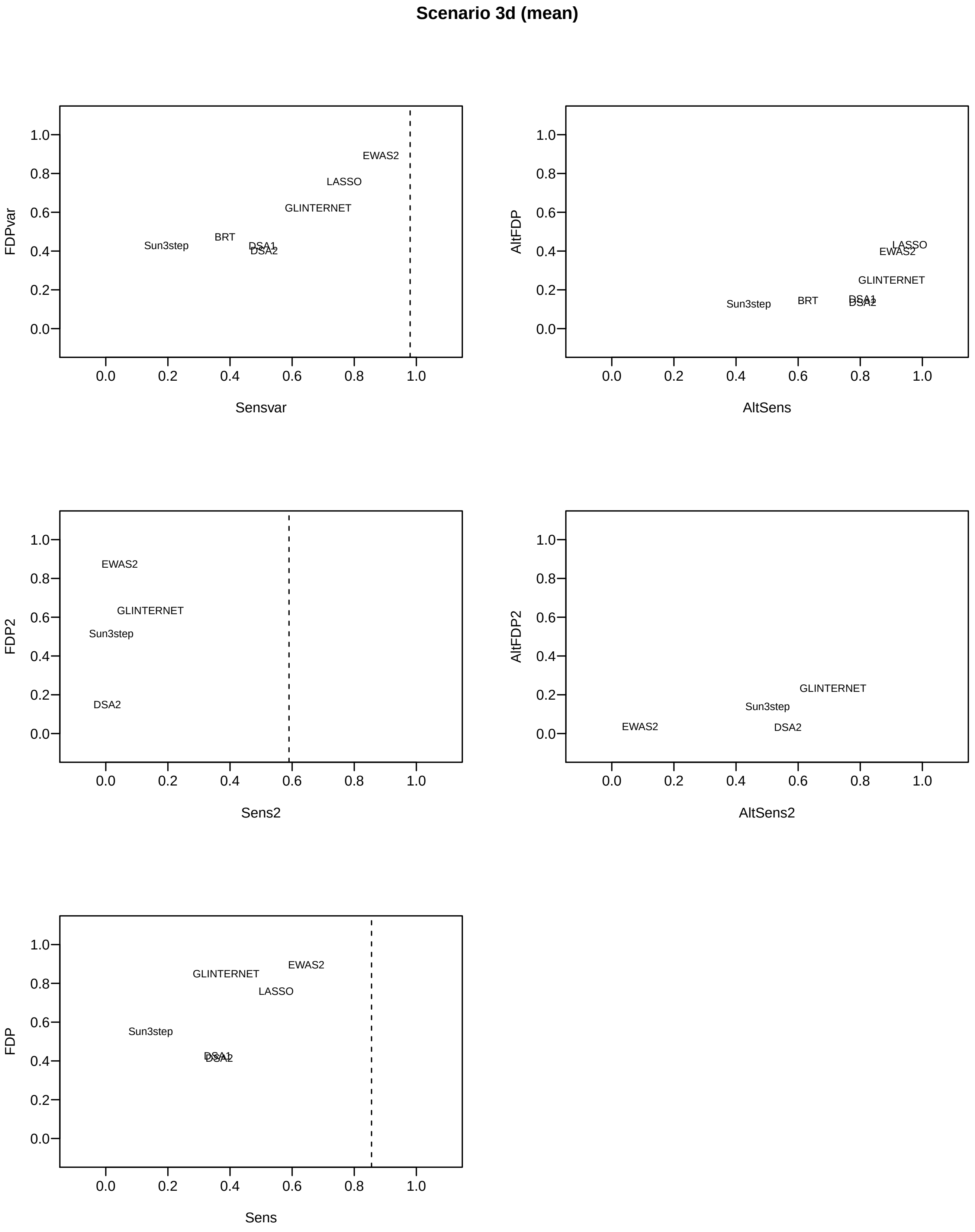

Figure 21: Measures of sensitivity vs FDP in scenario 3d.

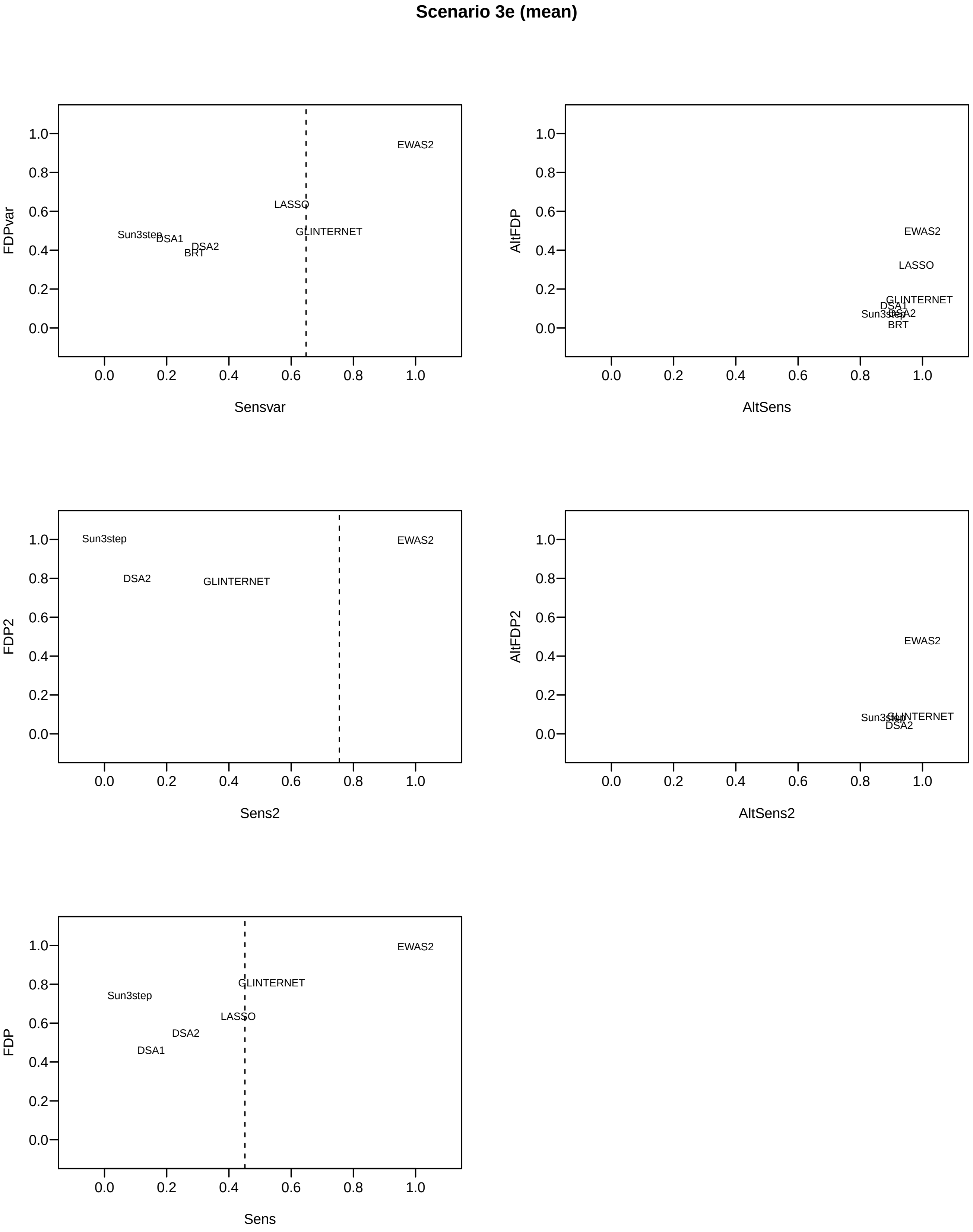

Figure 22: Measures of sensitivity vs FDP in scenario 3e.

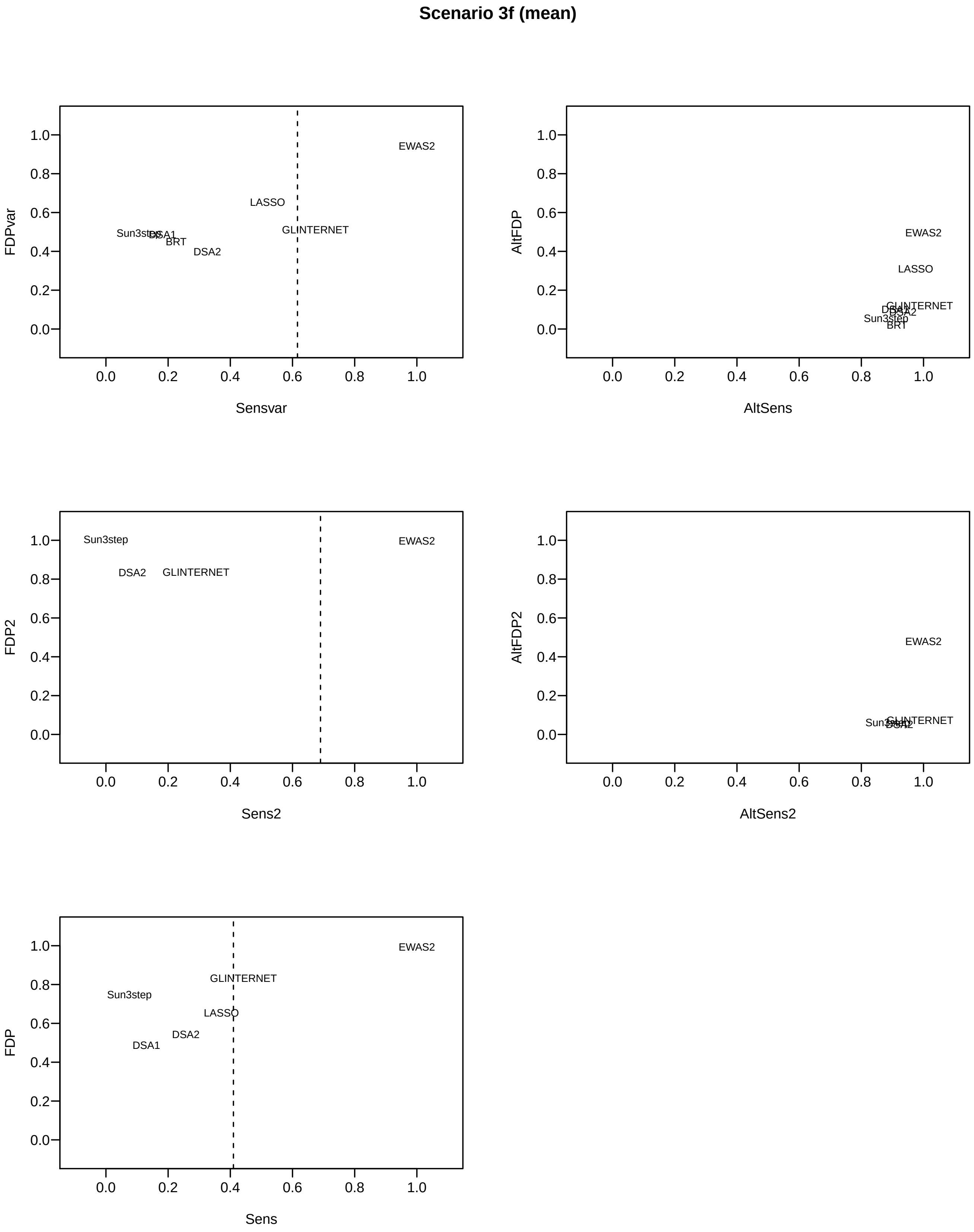

Figure 23: Measures of sensitivity vs FDP in scenario 3f.

Scenario 3g (mean)

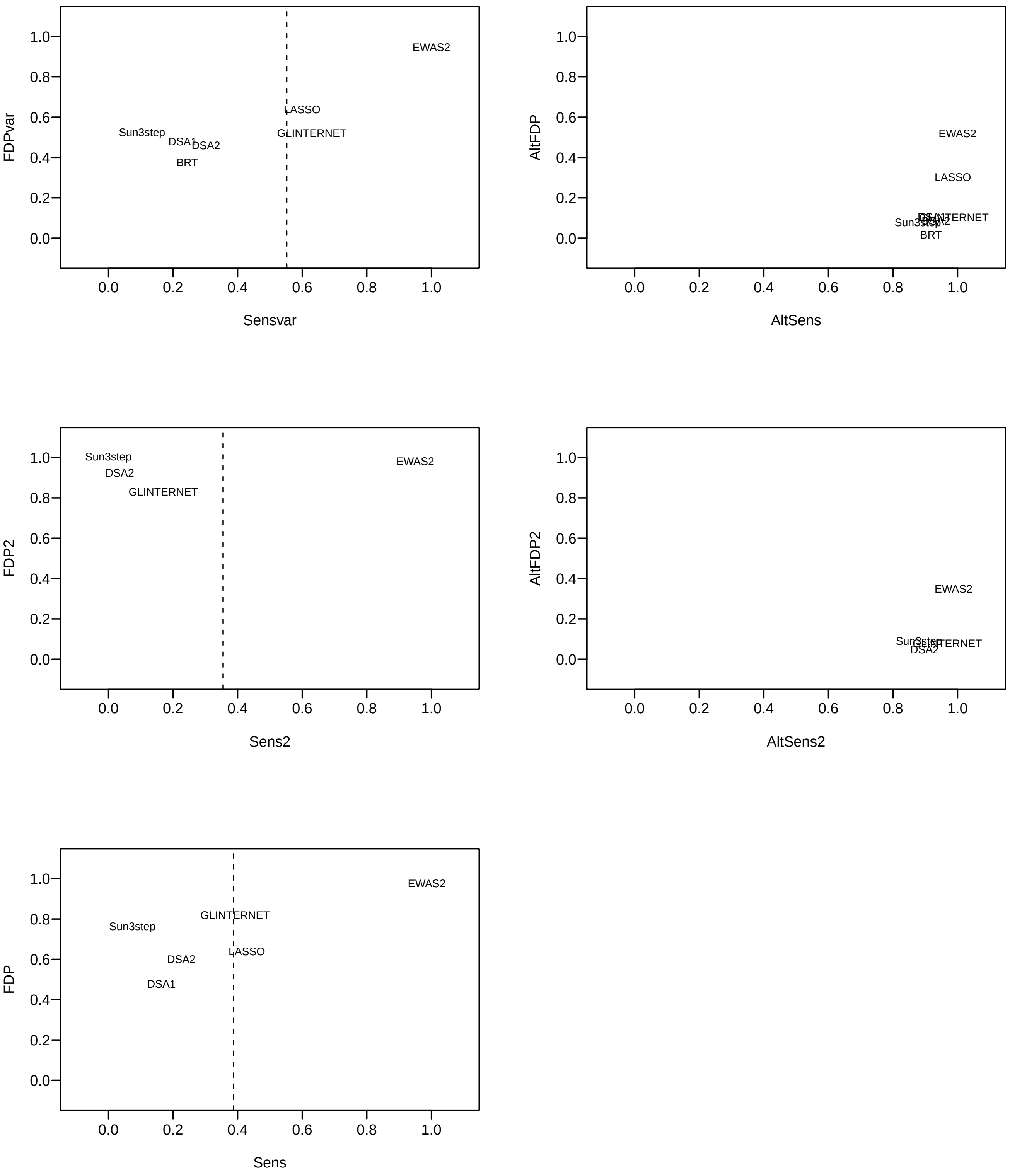

Figure 24: Measures of sensitivity vs FDP in scenario 3g.

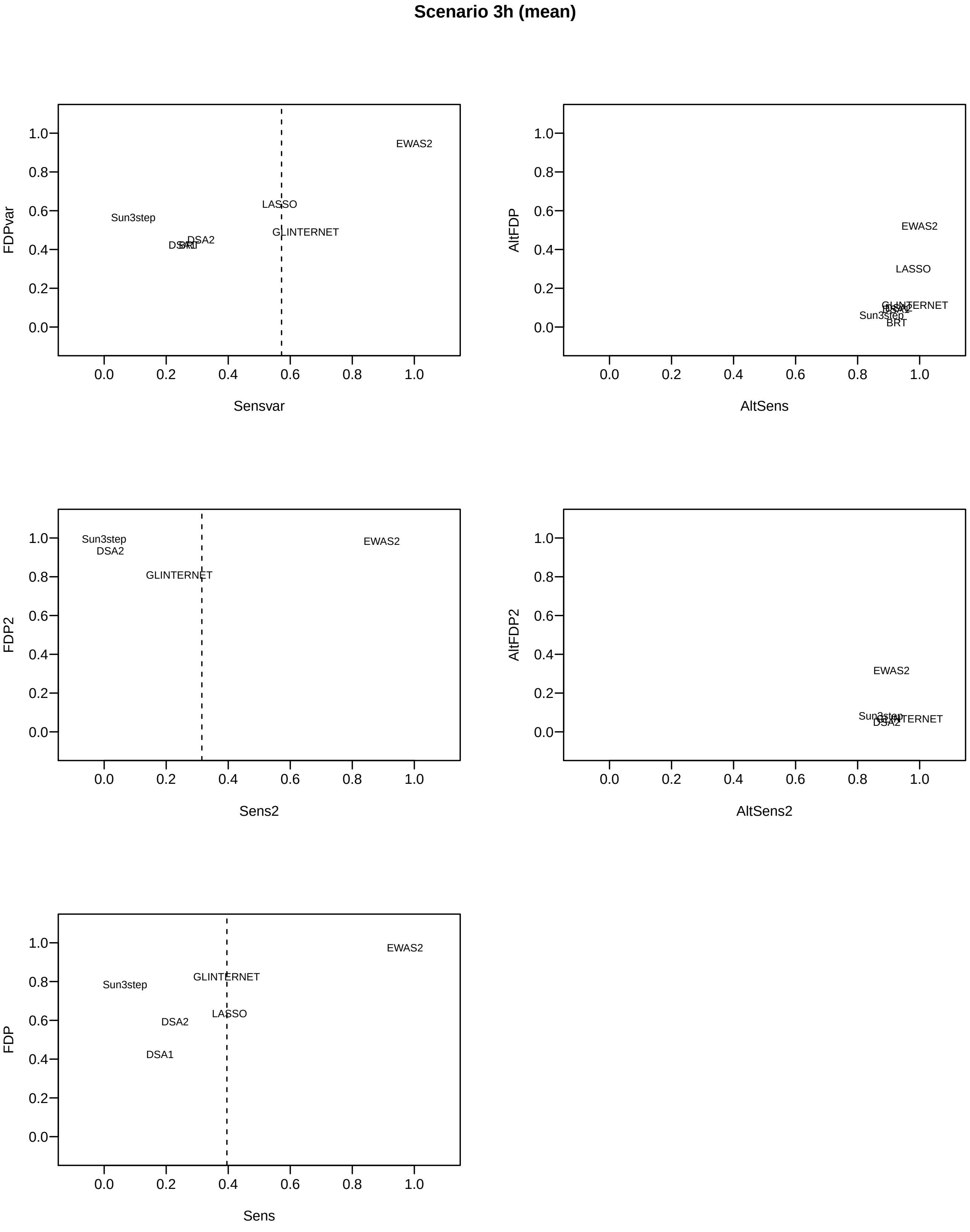

Figure 25: Measures of sensitivity vs FDP in scenario 3h.

Figure 26: Performance of the compared methods in terms of AltSens$_2$. Mean values based on 100 simulations. The vertical line separates scenarios according to the pairwise correlation between the true predictors as "mixed" (any exposure can be selected as a true predictor regardless of correlation), or "high" (exposures are choosen so that all their pairwise correlations are above 0.6). Scenarios 1, 2 and 3 involve no interactions, one two-way inetraction, and two two-way interactions, respectively.

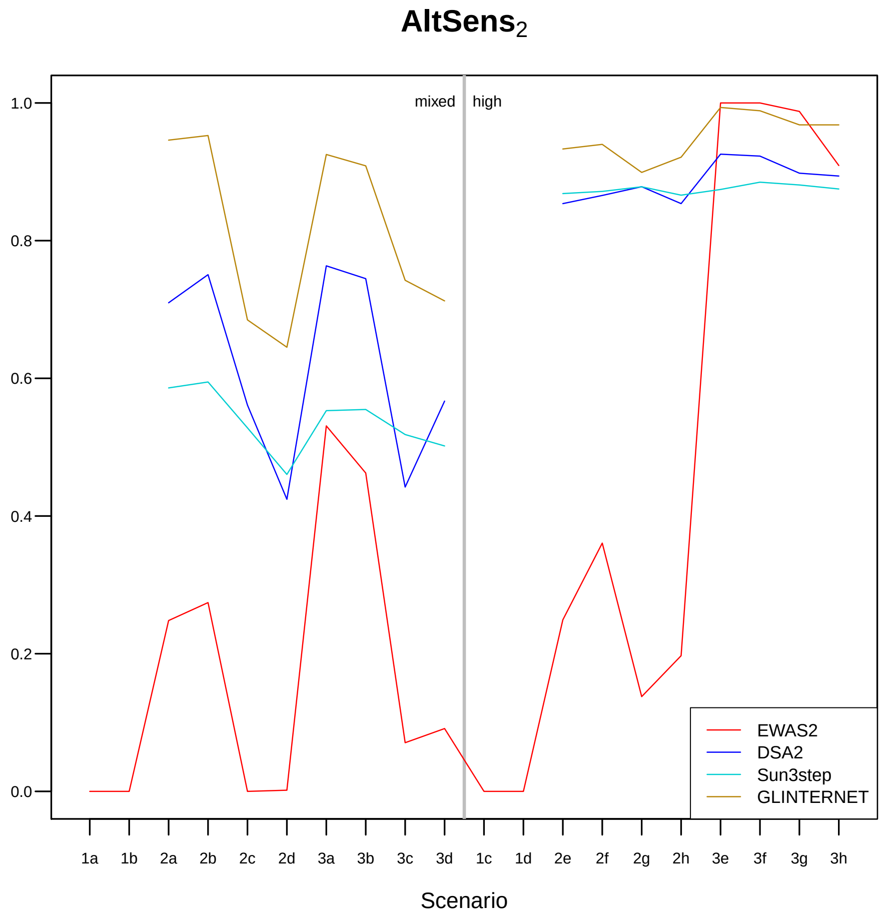

Figure 27: Performance of the compared methods in terms of AltFDP$_2$. Mean values based on 100 simulations. The vertical line separates scenarios according to the pairwise correlation between the true predictors as “mixed” (any exposure can be selected as a true predictor regardless of correlation), or “high” (exposures are choosen so that all their pairwise correlations are above 0.6). Scenarios 1, 2 and 3 involve no interactions, one two-way inetraction, and two two-way interactions, respectively.

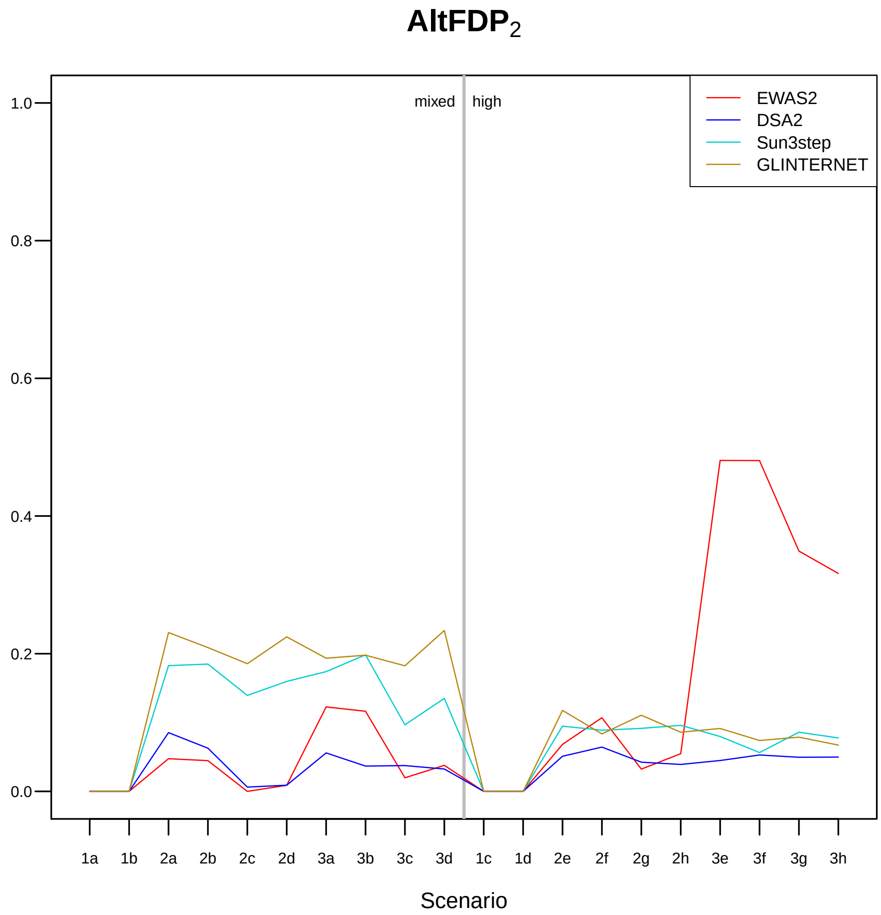

L Sensitivity analyses for the impact of low pairwise correlation among the true predictors

The new scenario 2i was created to assess the impact low pairwise correlation among the true predictors on the performance of the methods analysed. Scenario 2i is comparable to scenarios 2a and 2e (Table 22) regarding the remaining tuning parameters. Results are shown in Figure 28 (RMS, RNV and R^2_{rel}), Figure 29 (sensitivity measures) and Figure 30 (specificity measures).

Figure 28: Impact of the pairwise correlation level among the true predictors in scenario 2, in terms of model size (i.e. number of terms in the fitted model) and predictive ability. Scenario 2i corresponds to "low" pairwise correlation (exposures are choosen so that all their pairwise correlations are below 0.1), scenario "2a" corresponds to "mixed" (any exposure can be selected as a true predictor regardless of correlation), and scenario "2e" corresponds to "high" (exposures are choosen so that all their pairwise correlations are above 0.6). a) Relative model size (RMS) in log scale, b) Relative number of variables (RNV) in log scale, and c) Relative out-of-sample R^2 (R^2_{rel}). Mean values based on 100 simulations.

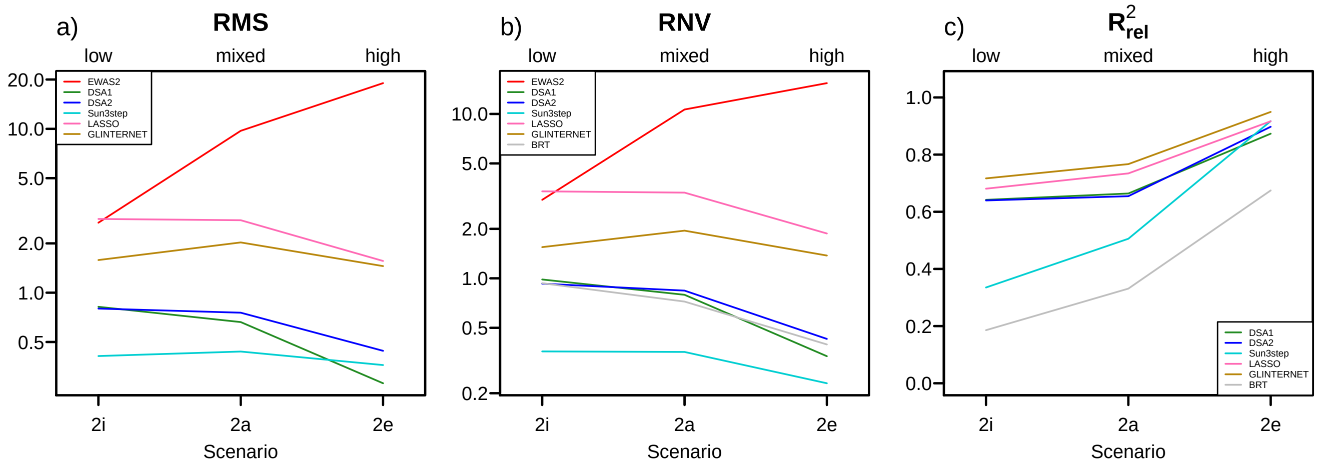

Table 22: New scenario 2i to assess the impact of low pairwise correlation among the true predictors on the performance of the methods analysed. Scenarios 2a and 2e are also shown for comparison purposes. Pairwise correlation of the predictors: “mixed”, when selecting the predictors among the whole exposome, in which case the absolute pairwise correlation ranged from 0.0000 to 1.0000; “high”, when selecting the predictors among the subset of the 13 variables in the exposome for which all absolute pairwise correlations were 0.62 or higher; or “low” (scenario 2i), when selecting the predictors among the subset of the 13 variables in the exposome for which all absolute pairwise correlations were 0.10 or lower. Values for the adjusted R^2 and sensitivities correspond to the mean and percentiles 2.5 and 97.5 as a result of fitting the model to 100 simulated datasets. See further details in Table 1.

Subscenario	Adjusted R^2	Pairwise corr.	Interaction size (and sign)	Parameters			Sensitivity[a]	Sensitivity$_2^b$
Scenario 2. True model: $F(E) = \beta_0 + \beta_1 X_1 + \beta_2 X_2 + \beta_3 X_3 + \beta_4 X_4 + \beta_5 X_5 + \gamma_{12} X_1 X_2$ (Size = 6; Only one 2-way interaction)								
2a	0.09 (0.07, 0.14)	mixed	strong (+)	$\beta_0 = \beta_1 = \cdots = \beta_5 = 1$	$\gamma_{12} = 1$	$\sigma = 8.3$	0.96 (0.83, 1.00)	0.99 (1.00, 1.00)
2e	0.13 (0.11, 0.14)	high	strong (+)	$\beta_0 = \beta_1 = \cdots = \beta_5 = 1$	$\gamma_{12} = 1$	$\sigma = 12$	0.59 (0.17, 0.83)	0.93 (0.00, 1.00)
2i	0.08 (0.07, 0.10)	low	strong (+)	$\beta_0 = \beta_1 = \cdots = \beta_5 = 1$	$\gamma_{12} = 1$	$\sigma = 8.3$	0.99 (0.91, 1.00)	1.00 (1.00, 1.00)

a: Proportion of true terms detected by the fitted model. In models with interaction terms, the main effects of variables involved in true interactions detected are assumed to be also detected.
b: Proportion of true interaction terms detected by the fitted model.

Figure 29: Impact of the pairwise correlation level among the true predictors in scenario 2, in terms of sensitivity. Scenario 2i corresponds to "low" pairwise correlation (exposures are choosen so that all their pairwise correlations are below 0.1), scenario "2a" corresponds to "mixed" (any exposure can be selected as a true predictor regardless of correlation), and scenario "2e" corresponds to "high" (exposures are choosen so that all their pairwise correlations are above 0.6). a) Relative model size (RMS), b) Relative number of variables (RNV), and c) Relative out-of-sample R^2 (R^2_{rel}). Mean values based on 100 simulations.

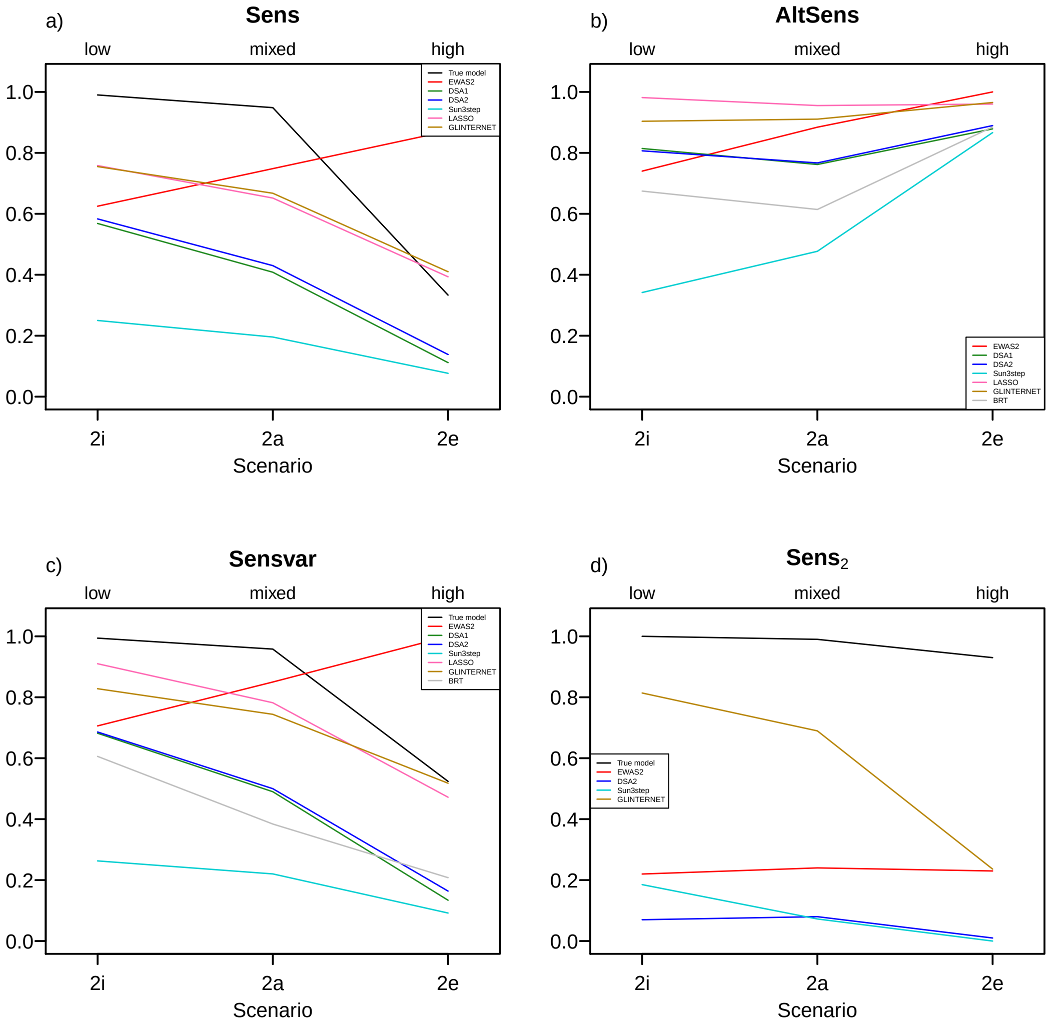

Figure 30: Impact of the pairwise correlation level among the true predictors in scenario 2, in terms of sensitivity. Scenario 2i corresponds to “low” pairwise correlation (exposures are choosen so that all their pairwise correlations are below 0.1), scenario “2a” corresponds to “mixed” (any exposure can be selected as a true predictor regardless of correlation), and scenario “2e” corresponds to “high” (exposures are choosen so that all their pairwise correlations are above 0.6). a) Relative model size (RMS), b) Relative number of variables (RNV), and c) Relative out-of-sample R^2 (R^2_{rel}). Mean values based on 100 simulations.

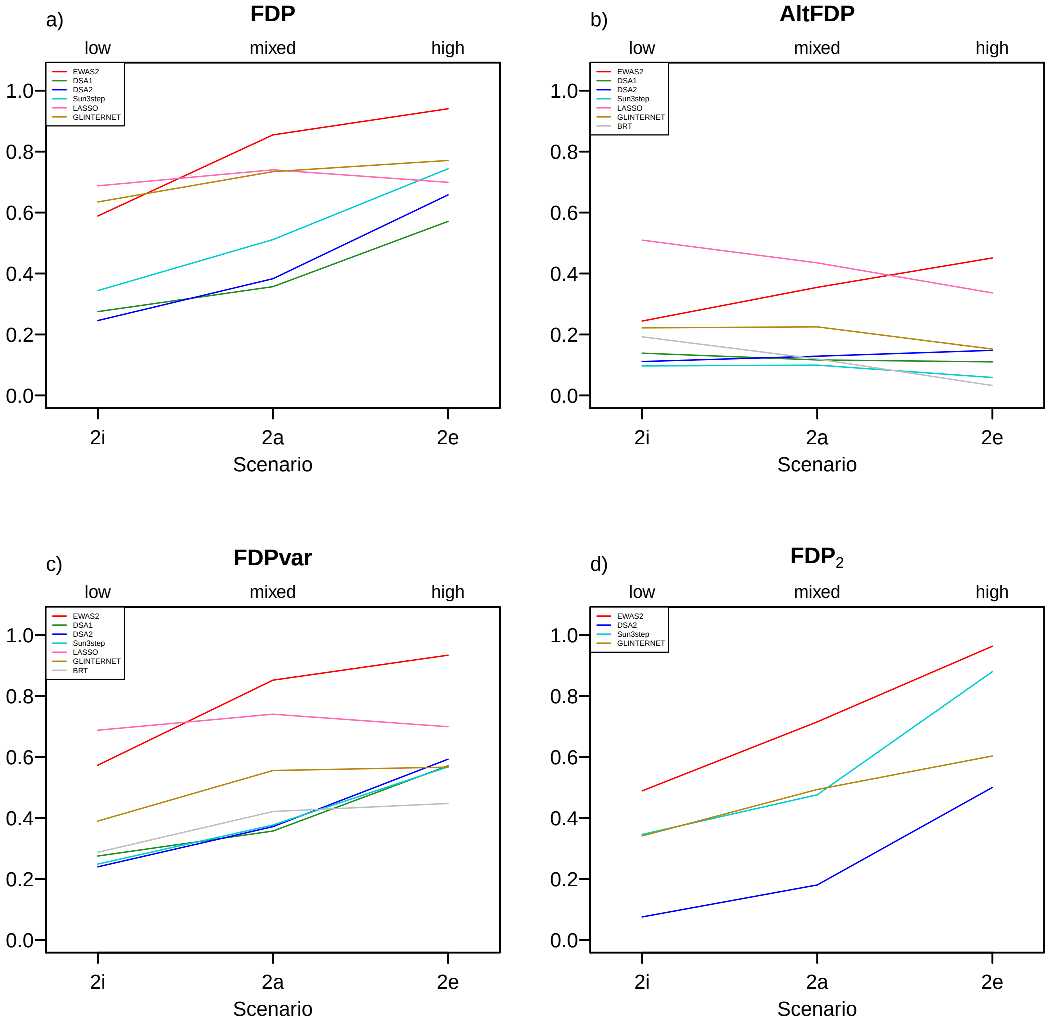

M Sensitivity analyses on the main tuning parameters

All analyses were performed in scenario 2e using 100 simulations unless otherwise stated.

M.1 DSA$_2$

We made the following setting changes:

- Setting in our simulations:
  - Maximum model size: 10
  - Number of folds for cross-validation: 5.
- New setting for sensitivity analysis:
  - Maximum model size: 20
  - Number of folds for cross-validation: 10.

Results showed no impact of the tuning changes except a slightly, non signifficant increase in the mean model size (specifically, from 2.6 to 3.1 while the true model size was 6). Details are shown in Figure 31.

M.2 LASSO

In all scenarios, we changed the number of folds for cross-validation as follows:

- Setting in our simulations: 3-folds.
- New setting for sensitivity anslysis: 10-folds.

Results showed no impact in terms of R^2_{rel} or measures of sensitivity. In terms of model size, the 10-folds cross-validation gave slightly higher sizes. In terms of specificity, the 10-folds setting provided a tiny increase in the measures. Hence, increasing the folds for cross-validation seems to tend to select a few more wrong predictors. This impact was practically independent of the scenario. Details are shown in Figure 32.

M.3 GLINTERNET

We made the following setting changes:

- Setting in our simulations:
  - Number of lambda values: 20.
- New setting for sensitivity analysis:
  - Number of lambda values: 50.

Results showed no impact of increasing the number on lambda values except a slightly decrease in the mean model size (specifically, from 8.7 to 7.0 while the true model size was 6). Details are shown in Figure 33.

Figure 31: Sensitivity analysis on the tuning parameters for DSA_2. Points correspond to the mean value while segments correspond to percentiles 2.5% and 97.5%. Black corresponds to the setting in our simulations (maximum model size = 10; number of folds for cross-validation = 5). Red corresponds to the new setting (maximum model size = 20; number of folds for cross-validation = 10). Results based on 100 simulations.

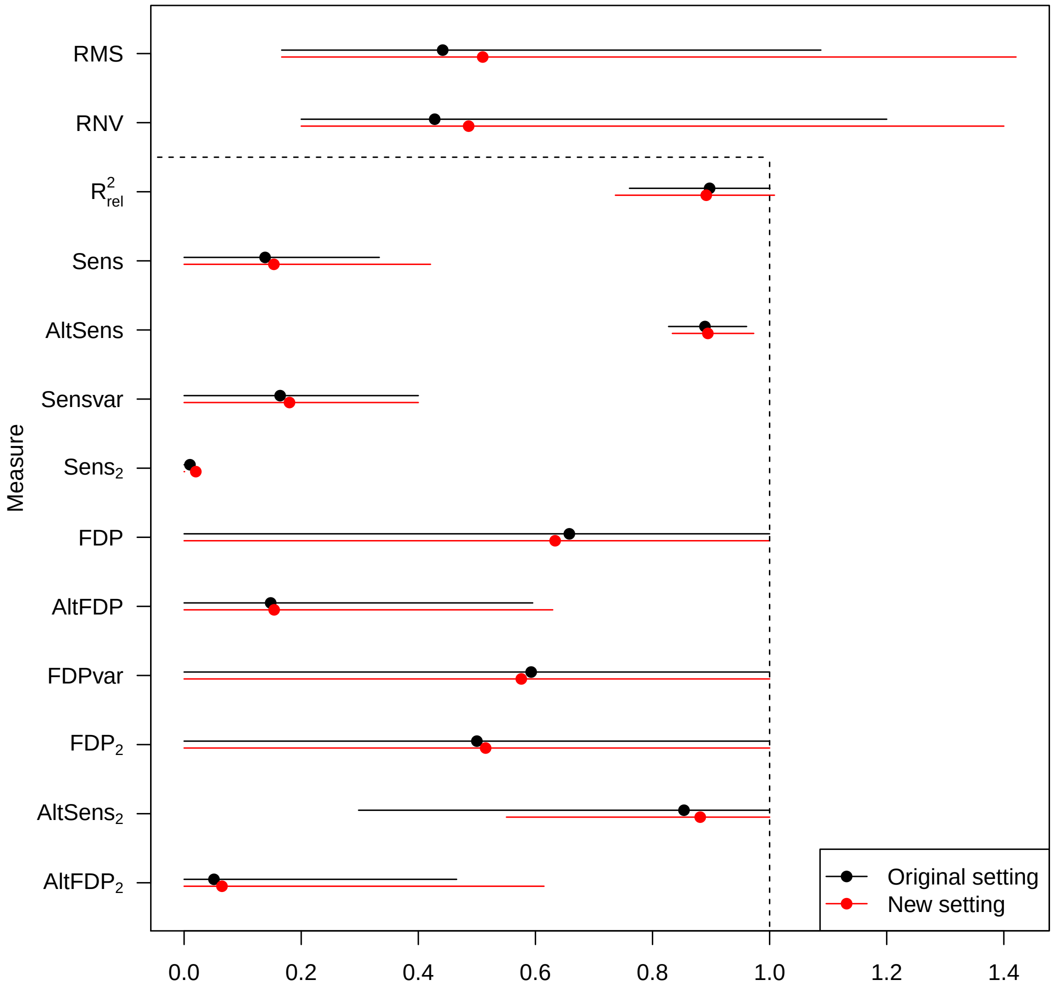

M.4 BRT

We made the following setting changes:

- Setting in our simulations:
  - Proportion of observations used in selecting variables: 0.5.
  - Number of folds for cross-validation: 10.
- New setting for sensitivity analysis:
  - Proportion of observations used in selecting variables: 0.75.
  - Number of folds for cross-validation: 5.

Figure 32: Sensitivity analysis on number of folds for cross-validation in LASSO. Black corresponds to the setting in our simulations (i.e. 3-folds). Red corresponds to the new setting (i.e. 10-folds). Results based on 100 simulations.

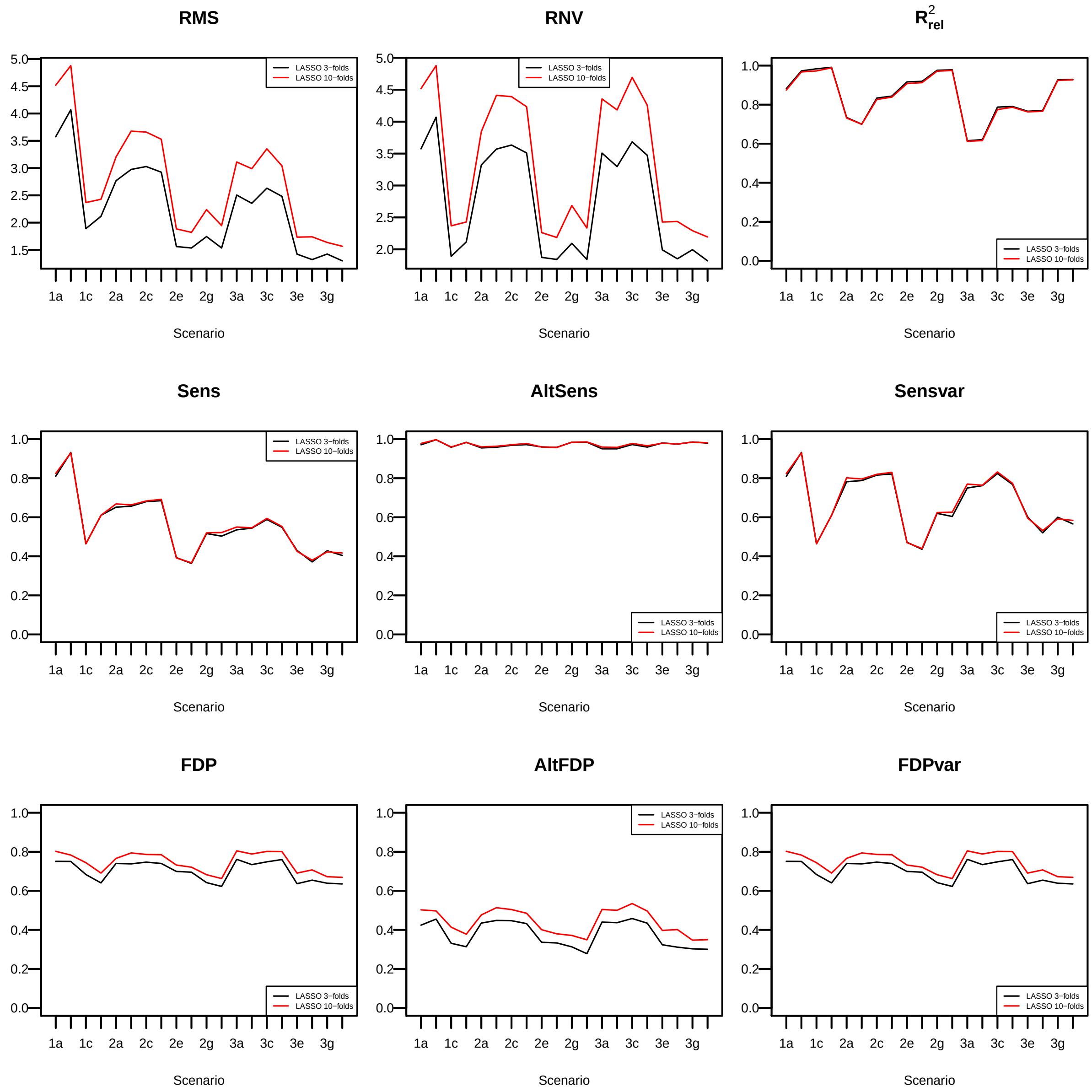

Results showed no impact of the changes in the tuning parameters except slightly variations in both the mean number of variables in the model (from 2.0 to 2.5 while it was 5 in the true model) and R^2_{rel} (from 0.67 to 0.49). Details are shown in Figure 34.

M.5 Sun3step

M.5.1 Step 1: correlation analysis

We performed a sensitivity analysis (in all scenarios) consisting in excluding the first step (i.e. correlation analysis). Results showed practically no impact in terms of $Rrel2$, sensitivity or specificity. In terms model size, some impact was observed only in some scenarios with high pairwise correlation among the true predictors. Specifically, when excluding this first step in Sun3step, the relative model

Figure 33: Sensitivity analysis on the tuning parameters for GLINTERNET. Points correspond to the mean value while segments correspond to percentiles 2.5% and 97.5%. Black corresponds to the setting in our simulations (number of lambda values = 20). Red corresponds to the new setting (number of lambda values = 50). Results based on 100 simulations.

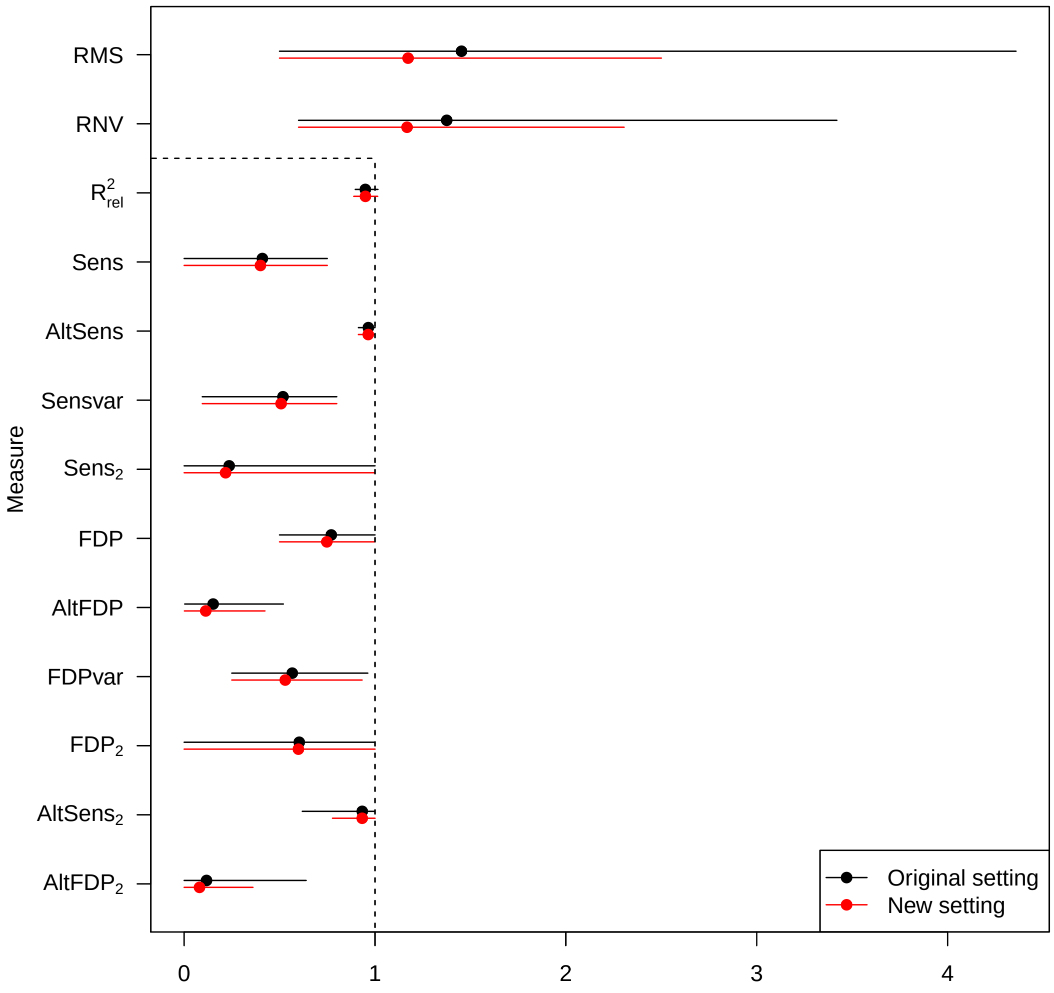

size approximately doubled in such scenarios. Details are shown in Figure 35.

M.5.2 Step 2: CART screening

We made the following setting changes:

- Setting in our simulations:
  - Number of folds for cross-validation: 10.
  - Maximum depth of the tree: 30.
- New setting for sensitivity analysis:
  - Number of folds for cross-validation: 5.
  - Maximum depth of the tree: 10.

Figure 34: Sensitivity analysis on the tuning parameters for BRT. Points correspond to the mean value while segments correspond to percentiles 2.5% and 97.5%. Black corresponds to the setting in our simulations (proportion of observations used in selecting variables = 0.5; number of folds for cross-validation = 10). Red corresponds to the new setting (proportion of observations used in selecting variables = 0.75; number of folds for cross-validation = 5). Results based on 100 simulations.

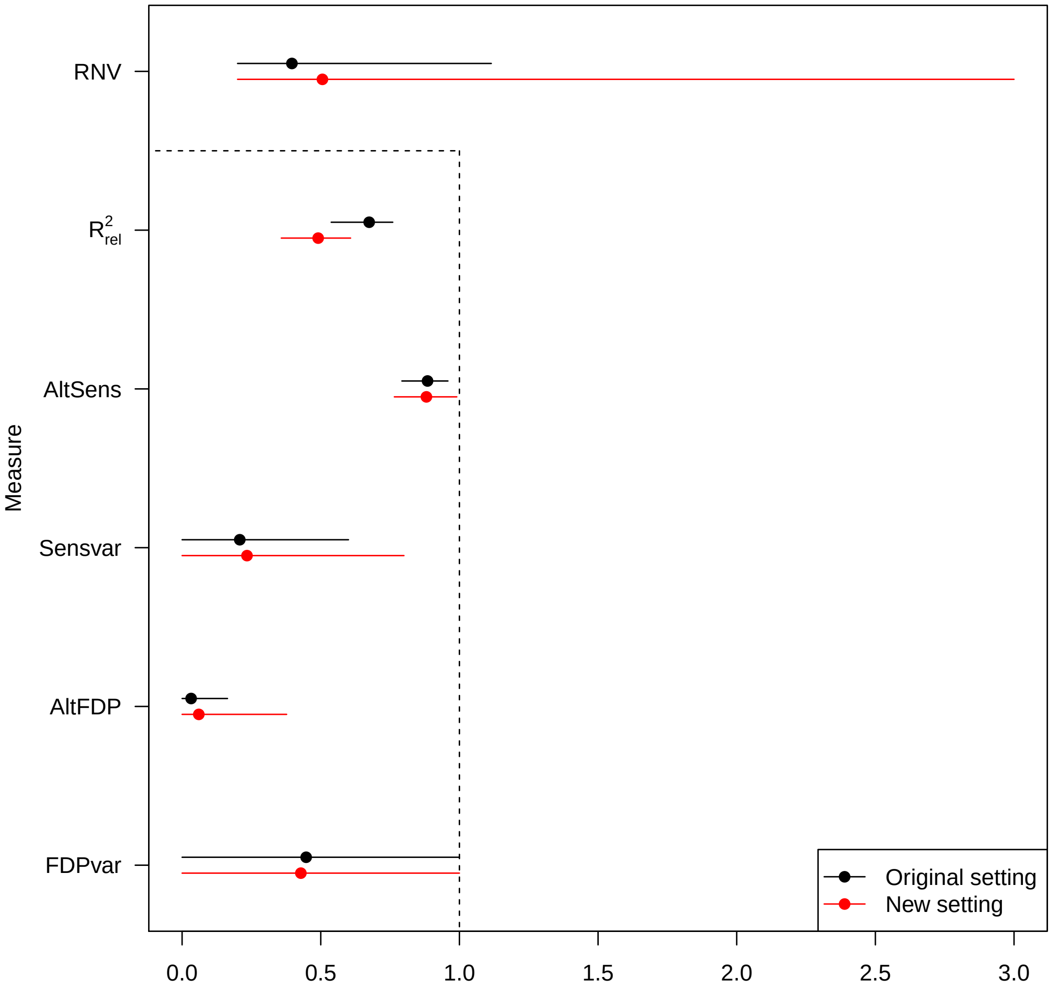

Results showed no impact of the changes in the tuning parameters (see Figure 36).

M.5.3 Step 3: DSA_2 fitting

We made the following setting changes:

- Setting in our simulations:
  - Maximum model size: 10
  - Number of folds for cross-validation: 5.
- New setting for sensitivity analysis:
  - Maximum model size: 20

Figure 35: Sensitivity analysis on correlation analysis for Sun3step. Black corresponds to the setting in our simulations. Red corresponds to the same setting but excluding the correlation analysis in the first step. Results based on 100 simulations.

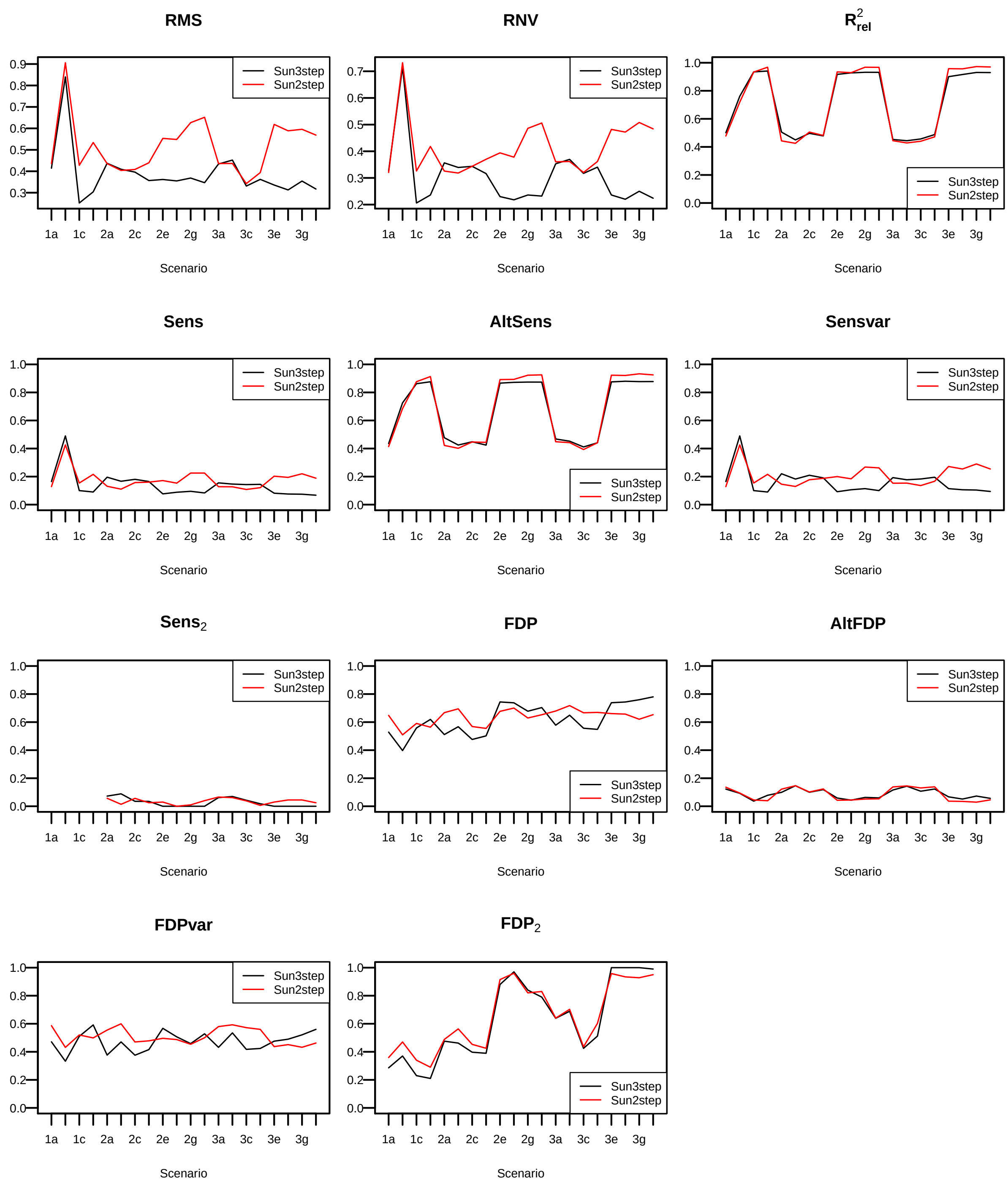

– Number of folds for cross-validation: 10.

Results showed no impact of the changes in the tuning parameters (see Figure 37).

Figure 36: Sensitivity analysis on the tuning parameters in step 2 (CART screening) of Sun3step. Points correspond to the mean value while segments correspond to percentiles 2.5% and 97.5%. Black corresponds to the setting in our simulations (number of folds for cross-validation = 10; maximum depth of the tree = 30). Red corresponds to the new setting (number of folds for cross-validation = 5; maximum depth of the tree = 10). Results based on 100 simulations.

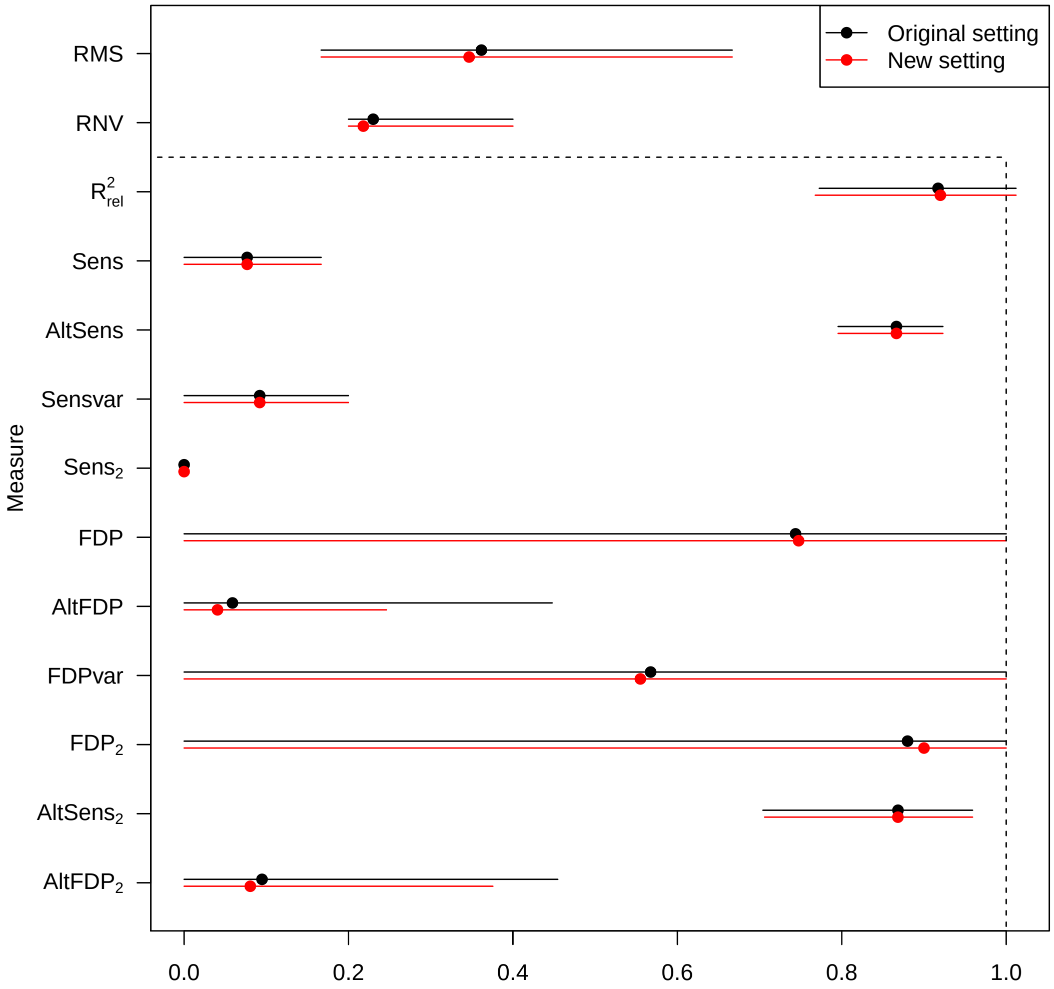

M.6 Summary of results

For DSA_2, increasing both the maximum model size and the number of folds for cross-validation resulted only in slightly, non significant increases in the mean model size. For LASSO, increasing the number of folds for cross-validation resulted in selecting a few more wrong predictors while R^2_{rel} and measures of sensitivity remained invariant. For GLINTERNET, increasing the number on lambda values implied only a slightly, non significant decrease in the mean model size. For BRT, increasing the proportion ob observations used in selecting variables and reducing the number of folds for cross-validation resulted in a slightly reduction of R^2_{rel}. For Sun3step, removing the first step (correlation analysis) showed an impact only in some scenarios with high pairwise correlation among the true predictors. Specifically, the relative model size approximately doubled in such scenarios. No impact on changing the main tuning parameters in steps 2 and 3 were observed.

Figure 37: Sensitivity analysis on the tuning parameters in step 3 (DSA_2 fitting) of Sun3step. Points correspond to the mean value while segments correspond to percentiles 2.5% and 97.5%. Black corresponds to the setting in our simulations (maximum model size = 10; number of folds for cross-validation = 5). Red corresponds to the new setting (maximum model size = 20; number of folds for cross-validation = 10). Results based on 100 simulations.

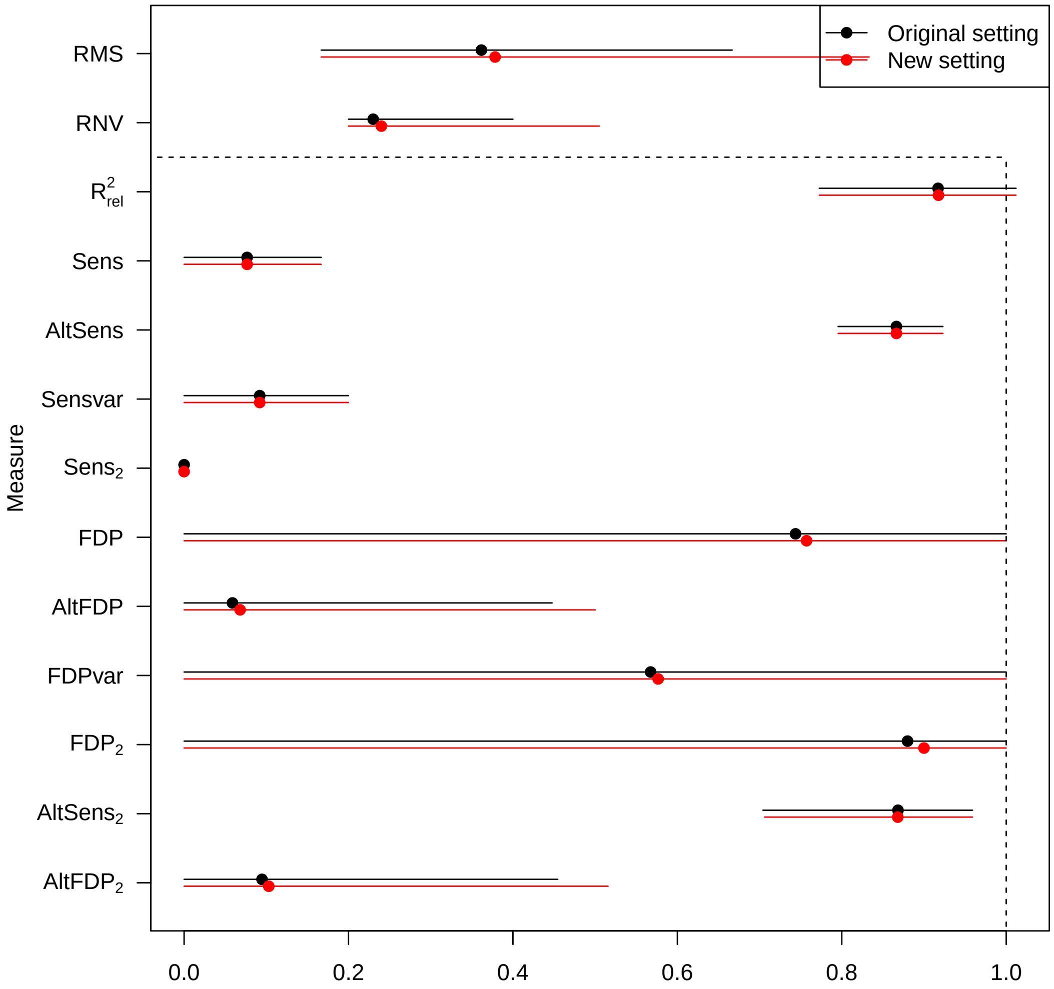

References

[1] Yorifuji T, Debes F, Weihe P, Grandjean P. Prenatal exposure to lead and cognitive deficit in 7- and 14-year-old children in the presence of concomitant exposure to similar molar concentration of methylmercury. Neurotoxicol Teratol. 2011;33(2):205-211.

[2] Sinisi SE, van der Laan MJ. Deletion/substitution/addition algorithm in learning with applications in genomics. Stat Appl Genet Mol Biol. 2004;3:Article18.
